# Supplementary material for: Incidence of maternal peripartum infection: A systematic review and meta-analysis
Source: PLoS Med. 2019 Dec 10;16(12):e1002984. doi: 10.1371/journal.pmed.1002984 (PMC6903710; doi:10.1371/journal.pmed.1002984)
Supplement: S1 Checklist — PRISMA, Preferred Reporting Items for Systematic Reviews and Meta-Analyses. (DOC) [file pmed.1002984.s001.doc]

| **Section/topic** | **#** | **Checklist item** | **Reported on page #** |
| --- | --- | --- | --- |
| **TITLE** | | |  |
| Title | 1 | Identify the report as a systematic review, meta-analysis, or both. | Title: A systematic review and meta-analysis |
| **ABSTRACT** | | |  |
| Structured summary | 2 | Provide a structured summary including, as applicable: background; objectives; data sources; study eligibility criteria, participants, and interventions; study appraisal and synthesis methods; results; limitations; conclusions and implications of key findings; systematic review registration number. | Abstract |
| **INTRODUCTION** | | |  |
| Rationale | 3 | Describe the rationale for the review in the context of what is already known. | Introduction paragraph 1. “Infection is an important preventable cause of maternal morbidity and mortality… However, the frequency of infection in pregnancy is poorly understood; a review of maternal morbidity identified no published systematic literature review of infection incidence, making it the one major direct cause of maternal morbidity without such a review” |
| Objectives | 4 | Provide an explicit statement of questions being addressed with reference to participants, interventions, comparisons, outcomes, and study design (PICOS). | Introduction paragraph 3  “This review focusses on recent epidemiological evidence for the incidence of ‘maternal peripartum infection’, defined by the World Health Organization (WHO) in 2015 to encompass infections of the genital tract and surrounding tissues from onset of labour or rupture of membranes until 42 days postpartum” |
| **METHODS** | | |  |
| Protocol and registration | 5 | Indicate if a review protocol exists, if and where it can be accessed (e.g., Web address), and, if available, provide registration information including registration number. | Methods paragraph 1 PROPSERO CRD42017074591 |
| Eligibility criteria | 6 | Specify study characteristics (e.g., PICOS, length of follow-up) and report characteristics (e.g., years considered, language, publication status) used as criteria for eligibility, giving rationale. | Methods/*Exclusion criteria*  “All identified studies were systematically assessed, irrespective of language or study design”  Exclusions included studies with:  “Only a subgroup of women at higher risk of infection than the general population of peripartum women (e.g. only caesarean section deliveries or only women with diabetes)  Data collected before 1990. If a study spanned 1990 but disaggregated by year, data from 1990 onwards were used  Conference and poster abstracts”  Methods/*Outcome definitions*  The WHO definition of Maternal Peripartum Infection  “We considered this to encompass specific constituent infections, namely chorioamnionitis in labour, puerperal endometritis, and wound infection following caesarean section, perineal tear or episiotomy. We included sepsis occurring within the defined time-period, restricted to sepsis of genital tract or wound origin where possible.” |
| Information sources | 7 | Describe all information sources (e.g., databases with dates of coverage, contact with study authors to identify additional studies) in the search and date last searched. | Methods/*Search strategy*  “We searched Medline, EMBASE, Global Health, Popline, CINAHL, the Latin American and Caribbean Health Science Information (LILACS), Africa-Wide Information and regional WHO on-line databases using Global Index Medicus from January 2005 to June 2016.”  Methods/Screening and data extraction |
| Search | 8 | Present full electronic search strategy for at least one database, including any limits used, such that it could be repeated. | S1 Appendix  Search strategies for all databases included |
| Study selection | 9 | State the process for selecting studies (i.e., screening, eligibility, included in systematic review, and, if applicable, included in the meta-analysis). | Methods/*Exclusion criteria*  Studies were excluded if their titles or abstracts met the listed exclusion criteria  “We sought the full-text for all remaining studies, including those where the abstract had insufficient information to make a decision. The same exclusion criteria applied to full texts.”  Methods/*Screening and data extraction*  “SW and AM double-screened 300 (~1%) title and abstracts to ensure consistency; the rest were single-screened. Full-text screening and extraction was conducted by SW, AM and MB, with approximately 8% of articles double-screened and extracted to ensure consistency. Queries were resolved through discussion and when necessary with input from a third reviewer (OC).” |
| Data collection process | 10 | Describe method of data extraction from reports (e.g., piloted forms, independently, in duplicate) and any processes for obtaining and confirming data from investigators. | Methods/*Screening and data extraction*  As above – 8% of articles were extracted in duplicate.  “Nine authors were contacted to clarify study eligibility.” |
| Data items | 11 | List and define all variables for which data were sought (e.g., PICOS, funding sources) and any assumptions and simplifications made. | Methods/*Screening and data extraction*  “Data extracted included language, location and dates of study, study population, study design, sampling, outcome definition, denominator, time-period for observing infection, data source, diagnosis, and incidence of infection”  Full details in S2 Appendix |
| Risk of bias in individual studies | 12 | Describe methods used for assessing risk of bias of individual studies (including specification of whether this was done at the study or outcome level), and how this information is to be used in any data synthesis. | Methods/*Critical appraisal of studies*  “We appraised the quality of each study outcome according to criteria in Table 1, adapted from Joanna Briggs Institute criteria for assessing incidence/prevalence studies”  Table 1. – Quality Assessment Criteria  Assess for selection bias, attrition bias and measurement bias.  Table 2. Standard definitions for infection outcomes  Used to assess measurement bias  Methods/*Data management and analysis* paragraph 3  Subgroup analysis of studies meeting all quality criteria  “to obtain a weighted pooled estimate of incidence of each infection outcome, for 1) all studies, 2) high quality studies” |
| Summary measures | 13 | State the principal summary measures (e.g., risk ratio, difference in means). | Methods/*Data management and analysis* paragraph 3  “a weighted pooled estimate of incidence of each infection outcome” |
| Synthesis of results | 14 | Describe the methods of handling data and combining results of studies, if done, including measures of consistency (e.g., I^2^) for each meta-analysis. | Methods/*Data management and analysis* paragraph 4  “Infection incidence risk (as a proportion) was transformed using the Freeman-Tukey transformation to approximate a normal distribution and stabilise the variance. Because study designs and outcome definitions varied, we used random effects to combine study estimates. The tau^2^ measure of between-study heterogeneity was estimated using restricted maximum likelihood. The pooled estimates were back-transformed and results presented as proportions.” |

Page 1 of 2

| **Section/topic** | **#** | **Checklist item** | **Reported on page #** |
| --- | --- | --- | --- |
| Risk of bias across studies | 15 | Specify any assessment of risk of bias that may affect the cumulative evidence (e.g., publication bias, selective reporting within studies). | Methods/*Data management and analysis* paragraph 3  Subgroup analysis of studies meeting all quality criteria – at low-risk of bias  “to obtain a weighted pooled estimate of incidence of each infection outcome, for 1) all studies, 2) high quality studies” |
| Additional analyses | 16 | Describe methods of additional analyses (e.g., sensitivity or subgroup analyses, meta-regression), if done, indicating which were pre-specified. | Methods/*Data management and analysis* paragraph 3  Pre-specified subgroups – high quality and world regions  “weighted pooled estimate of incidence of each infection outcome, for 1) all studies, 2) high quality studies, and 3) stratified by world region”  Methods/*Data management and analysis* paragraph 4  Sensitivity analysis  “As sensitivity analyses we calculated standardised residuals and removed outliers with p>0.05 (based on the t distribution). We compared heterogeneity and precision intervals before and after the removal of outliers.”  Methods/*Data management and analysis* paragraph 5  Pre-specified meta-regression of world region and study characteristics  “We used meta-regression and reported odds ratios (OR) to explore whether world region or study characteristics influenced infection incidence. Infection risk was log-transformed and univariate random effects models used to explore associations between each variable and odds of infection.” |
| **RESULTS** | | |  |
| Study selection | 17 | Give numbers of studies screened, assessed for eligibility, and included in the review, with reasons for exclusions at each stage, ideally with a flow diagram. | Results/Paragraph 1  “Figure 1 shows the 31,528 potentially relevant articles identified, of which 1543 were eligible for full-text review after title and abstract screening. We could not find two full-texts. Of the remaining 1541 full-texts screened, 111 were included”  Reasons for exclusion indicated in the Flow Diagram, Figure 1 |
| Study characteristics | 18 | For each study, present characteristics for which data were extracted (e.g., study size, PICOS, follow-up period) and provide the citations. | S4 Tables 1-5 include extracted study characteristics |
| Risk of bias within studies | 19 | Present data on risk of bias of each study and, if available, any outcome level assessment (see item 12). | S5 Table of quality score (risk of bias) at study level for each study.  S4 tables 1-5 indicates the score at outcome level |
| Results of individual studies | 20 | For all outcomes considered (benefits or harms), present, for each study: (a) simple summary data for each intervention group (b) effect estimates and confidence intervals, ideally with a forest plot. | Summary data S4 Tables 1-5.  Forest plots for each outcome Fig. 2-6. |
| Synthesis of results | 21 | Present results of each meta-analysis done, including confidence intervals and measures of consistency. | Table 4 |
| Risk of bias across studies | 22 | Present results of any assessment of risk of bias across studies (see Item 15). | Table 4  Subgroup meta-analysis of high-quality studies only, |
| Additional analysis | 23 | Give results of additional analyses, if done (e.g., sensitivity or subgroup analyses, meta-regression [see Item 16]). | Sensitivity analysis of potential outliers “We identified six outlier estimates, all with high infection incidence… Removal of these outliers did not change I^2^ but led to important reductions in both tau^2^ and prediction intervals -- therefore meta-analyses results are presented after removing these outliers.”    Subgroup analysis by world region for each outcome: forest plots Fig. 2-6.  Meta-regression Tables 5-9 |
| **DISCUSSION** | | |  |
| Summary of evidence | 24 | Summarize the main findings including the strength of evidence for each main outcome; consider their relevance to key groups (e.g., healthcare providers, users, and policy makers). | Discussion paragraph 1 – Main findings.  “Pooled infection incidence in high-quality studies was 3.9% for chorioamnionitis, 1.6% for endometritis, 1.2% for wound infection and 1.1% for maternal peripartum infection. Pooled incidence of sepsis was 0.05%.”  Relevance is presented in the conclusion  “To our knowledge this is the first global systematic review of maternal peripartum infection incidence. It demonstrates that infection is an important complication of childbirth…. At a time of growing concern about AMR, these findings highlight the importance for clinicians and policy-makers to focus efforts on improved infection prevention practices to reduce this preventable cause of maternal morbidity.” |
| Limitations | 25 | Discuss limitations at study and outcome level (e.g., risk of bias), and at review-level (e.g., incomplete retrieval of identified research, reporting bias). | Discussion paragraph 3 – risk of bias at study and outcome level  “The quality of many studies was poor, with potential for bias. Measurement bias was possible in 63% of studies, primarily because the infection was not defined or the definition used was too broad and risked over-estimating incidence.”  Discussion paragraph 5 – limitations from ‘study extent’  “For chorioamnionitis, wound infection and maternal peripartum infection there was evidence that study extent was associated with infection risk. Pooled incidence was up to five times higher in single-facility studies compared to estimates using nationally-representative databases”  Discussion paragraph 6 – limitations from study follow-up period  “Longer follow-up (risk) period was unsurprisingly associated with higher sepsis incidence, and a similar trend was observed with wound infection but lacked statistical evidence.”  Discussion/Strengths and weaknesses, paragraph 2 – limitations at review level. For example:  “we did not limit our review to population-level studies, potentially over-estimating infection incidence as discussed above. Conversely, we did include groups of low-risk women and so our pooled estimates may be an underestimate. “ |
| Conclusions | 26 | Provide a general interpretation of the results in the context of other evidence, and implications for future research. | Discussion/Conclusion  “To our knowledge this is the first global systematic review of maternal peripartum infection incidence. It demonstrates that infection is an important complication of childbirth. Moreover, we found that a large proportion of these infections occurred in labour with implications for the baby and the mother. Postpartum infection incidence appears lower than modelled global estimates, although the difference in definition limits comparability and the proportion of women affected is still considerable…. we also highlight the paucity of data from LMICs and the marked heterogeneity in study designs and infection definitions. Better quality research, using standard definitions and follow-up after hospital discharge, is required to improve comparability between different study settings and to demonstrate the influence of risk factors and protective interventions.” |
| **FUNDING** | | |  |
| Funding | 27 | Describe sources of funding for the systematic review and other support (e.g., supply of data); role of funders for the systematic review. | Uploaded separately |

*From:*  Moher D, Liberati A, Tetzlaff J, Altman DG, The PRISMA Group (2009). Preferred Reporting Items for Systematic Reviews and Meta-Analyses: The PRISMA Statement. PLoS Med 6(6): e1000097. doi:10.1371/journal.pmed1000097

For more information, visit: **www.prisma-statement.org**.

Page 2 of 2
