# Supplementary material for: Incidence of maternal peripartum infection: A systematic review and meta-analysis
Source: PLoS Med. 2019 Dec 10;16(12):e1002984. doi: 10.1371/journal.pmed.1002984 (PMC6903710; doi:10.1371/journal.pmed.1002984)
Supplement: S1 Text — (DOCX) [file pmed.1002984.s002.docx]

# S1 Text: Search Strategy

**Medline/EMBASE/Global Health**

1. Maternal text adj5 sepsis text
2. Maternal infection (text OR Mesh)
3. ((Maternal text adj2 complication text) OR maternal complications Mesh) AND infection
4. 1 or 2 or 3
5. Prevalence text
6. Prevalence Mesh
7. 5 or 6
8. 4 and 7
9. Restricted to Human/2005 Current

**Maternal/pregnancy terms**

| **Texts** |  |
| --- | --- |
| matern* OR pregnan* OR childbirth OR intrapartum OR intra-partum OR postpartum OR post-partum OR postnatal OR puerperal OR puerperium OR parturition OR obstetric OR labo*r OR partum OR deliver* OR perineal OR perineum OR caesarean |  |

**Sepsis**

| **Texts** |  |
| --- | --- |
| sepsis OR septic OR septic?em* OR endometritis OR metritis OR endomyometritis OR endoparametritis OR amnionitis OR placentitis OR membranitis OR infect* OR cervicitis OR vaginitis OR organ failure |  |

**Maternal Infection**

| **Texts** | **[MeSH]** |
| --- | --- |
| Chorioamnionitis OR ((puerperal or childbed or postpartum or post-partum) adj (fever or pyrexia)) OR puerperal peritonitis | Chorioamnionitis/ OR pregnancy complications, infectious/ OR puerperal infection/ |

**Maternal Complications with infection**

| **Texts** | **[MeSH]** |
| --- | --- |
| (Pregnan* or obstetric or postpartum or post-partum or maternal) adj2 (complication* or morbidit* or outcomes or near-miss) | Pregnancy complications/ OR obstetric labor complications/ or puerperal disorders/ |
| AND | |
| (sepsis or septic or fever or infection* or pyrexi*) |  |

**Prevalence/incidence/study**

| **Texts** | **[MeSH]** |
| --- | --- |
| prevalence OR proportion OR percent* OR frequency OR incidence OR rate* OR cohort OR longitudinal study OR follow-up study OR prospective study OR retrospective study OR cross-sectional OR intervention study OR trial OR community-based study OR population-based study OR observational study OR evaluat* OR audit OR epidemiology | prevalence/ OR incidence/ OR epidemiology/ OR epidemiologic methods/ OR clinical studies as topic/ OR epidemiologic studies/ |
| NOT | |
| case report* or comment or practice guideline* or editorial or consensus development conference or guideline* or legal case* or legislation or newspaper article or patient education handout or retracted publication |  |

**RESULTS**

- **Medline 10,934**
- **EMBASE 17732**
- **Global Health 6196**

**CINAHL plus - Results 4790**

**Using the terms above:**

1. Maternal text N5 sepsis text
2. Maternal infection (text OR Mesh)
3. ((Maternal text N2 complication text) OR maternal complications Mesh) AND infection
4. 1 or 2 or 3
5. Prevalence text
6. Prevalence Mesh
7. 5 or 6
8. 4 and 7
9. Restricted to 2005-Current & Excluded MEDLINE records

**Global Index Medicus – Results 1539.**

**Restricted to Western Pacific (WPRIM), Eastern Mediterranean (IMEMR), South-East Asian (IMSEAR) and Africa (AIM) Regions and the WHO library (WHOLIS) and 2005-2016.**

Search in title, abstract, subject

1. Maternal text
2. Sepsis text
3. Prevalence text
4. 1 and 2 and 3

**Maternal Text**

| matern* OR pregnan* OR childbirth OR intrapartum OR intra-partum OR postpartum OR post-partum OR postnatal OR puerperal OR puerperium OR parturition OR obstetric OR labo*r OR partum OR deliver* OR perineal OR perineum OR caesarean |
| --- |

**Sepsis Text**

| sepsis OR septic OR septicem* OR septicaem* OR endometritis OR metritis OR endomyometritis OR endoparametritis OR amnionitis OR placentitis OR membranitis OR infect* OR cervicitis OR vaginitis OR "organ failure" |
| --- |

**Prevalence Text**

| prevalence OR proportion OR percent* OR frequency OR incidence OR rate* OR cohort OR "longitudinal study" OR "follow-up study" OR "prospective study" OR "retrospective study" OR cross-sectional OR "intervention study" OR trial OR "community-based study" OR "population-based study" OR "observational study" OR evaluat* OR audit OR epidemiology |
| --- |

**POPLINE – Results 539**

**Restricted to 2005-2016**

| "matern* sepsis" ~5 OR "pregnancy sepsis" ~5 OR "childbirth sepsis" ~5 OR "intrapartum sepsis" ~5 OR "intra-partum sepsis" ~5 OR "puerperal sepsis" ~5 OR "postpartum sepsis" ~5 OR "post-partum sepsis" ~5 OR "postnatal sepsis" ~5 OR "puerperium sepsis" ~5 OR "parturition sepsis" ~5 OR "obstetric sepsis" ~5 OR "labor sepsis" ~5 OR "labour sepsis" ~5 OR "deliver* sepsis" ~5 OR "matern* infection*" ~5 OR "pregnancy infection*" ~5 OR "childbirth infection*" ~5 OR "intrapartum infection*" ~5 OR "intra-partum infection*" ~5 OR "puerperal infection*" ~5 OR "postpartum infection*" ~5 OR "post-partum infection*" ~5 OR "postnatal infection*" ~5 OR "puerperium infection*" ~5 OR "parturition infection*" ~5 OR "obstetric infection*" ~5 OR "labor infection*" ~5 OR "labour infection*" ~5 OR "deliver* infection*" ~5 OR "perineal infection*" ~5 OR "perineum infection*" ~5 OR "caesarean infection*" ~5 OR "puerperal fever" OR "childbed fever" OR "postpartum fever" OR "post-partum fever" OR "puerperal pyrexia" OR "postpartum pyrexia" OR "post-partum pyrexia" OR "puerperal peritonitis" OR chorioamnionitis OR endometritis |
| --- |

**Africa Wide Information – Results 3067**

**Restricted to 2005-Current**

| (matern* or pregnan* or childbirth or intrapartum or intra-partum or postpartum or post-partum or postnatal or puerperal or puerperium or parturition or obstetric or labo*r or partum or deliver* or perineal or perineum or caesarean) N5 (sepsis or septic or septic?em* or endometritis or metritis or endomyometritis or endoparametritis or amnionitis or placentitis or membranitis or infect* or pyrexi* or cervicitis or vaginitis or organ failure or chorioamnionitis or puerperal fever or childbed or puerperal peritonitis or Chorioamnionitis+ or puerperal infection+ ) |
| --- |

**LILACS – Results 1955**

| Matern? Or Embaraz? Or parto or alumbramiento or nacimiento or intraparto or postparto or postnatal or puerperal or puerperio or trabajo de parto or perineo or perineum or cesárea  AND  Sepsis or séptico or septicemia or endometritis or parametritis or amnionitis or infección or fiebre or cervicitis or vaginitis or falla sistémica or corioanmionitis or fiebre puerperal |
| --- |
