# Supplementary material for: Incidence of maternal peripartum infection: A systematic review and meta-analysis
Source: PLoS Med. 2019 Dec 10;16(12):e1002984. doi: 10.1371/journal.pmed.1002984 (PMC6903710; doi:10.1371/journal.pmed.1002984)
Supplement: S2 Text — (DOCX) [file pmed.1002984.s003.docx]

# S1 Text: Search Strategy

**Medline/EMBASE/Global Health**

1. Maternal text adj5 sepsis text
2. Maternal infection (text OR Mesh)
3. ((Maternal text adj2 complication text) OR maternal complications Mesh) AND infection
4. 1 or 2 or 3
5. Prevalence text
6. Prevalence Mesh
7. 5 or 6
8. 4 and 7
9. Restricted to Human/2005 Current

**Maternal/pregnancy terms**

| **Texts** |  |
| --- | --- |
| matern* OR pregnan* OR childbirth OR intrapartum OR intra-partum OR postpartum OR post-partum OR postnatal OR puerperal OR puerperium OR parturition OR obstetric OR labo*r OR partum OR deliver* OR perineal OR perineum OR caesarean |  |

**Sepsis**

| **Texts** |  |
| --- | --- |
| sepsis OR septic OR septic?em* OR endometritis OR metritis OR endomyometritis OR endoparametritis OR amnionitis OR placentitis OR membranitis OR infect* OR cervicitis OR vaginitis OR organ failure |  |

**Maternal Infection**

| **Texts** | **[MeSH]** |
| --- | --- |
| Chorioamnionitis OR ((puerperal or childbed or postpartum or post-partum) adj (fever or pyrexia)) OR puerperal peritonitis | Chorioamnionitis/ OR pregnancy complications, infectious/ OR puerperal infection/ |

**Maternal Complications with infection**

| **Texts** | **[MeSH]** |
| --- | --- |
| (Pregnan* or obstetric or postpartum or post-partum or maternal) adj2 (complication* or morbidit* or outcomes or near-miss) | Pregnancy complications/ OR obstetric labor complications/ or puerperal disorders/ |
| AND | |
| (sepsis or septic or fever or infection* or pyrexi*) |  |

**Prevalence/incidence/study**

| **Texts** | **[MeSH]** |
| --- | --- |
| prevalence OR proportion OR percent* OR frequency OR incidence OR rate* OR cohort OR longitudinal study OR follow-up study OR prospective study OR retrospective study OR cross-sectional OR intervention study OR trial OR community-based study OR population-based study OR observational study OR evaluat* OR audit OR epidemiology | prevalence/ OR incidence/ OR epidemiology/ OR epidemiologic methods/ OR clinical studies as topic/ OR epidemiologic studies/ |
| NOT | |
| case report* or comment or practice guideline* or editorial or consensus development conference or guideline* or legal case* or legislation or newspaper article or patient education handout or retracted publication |  |

**RESULTS**

- **Medline 10,934**
- **EMBASE 17732**
- **Global Health 6196**

**CINAHL plus - Results 4790**

**Using the terms above:**

1. Maternal text N5 sepsis text
2. Maternal infection (text OR Mesh)
3. ((Maternal text N2 complication text) OR maternal complications Mesh) AND infection
4. 1 or 2 or 3
5. Prevalence text
6. Prevalence Mesh
7. 5 or 6
8. 4 and 7
9. Restricted to 2005-Current & Excluded MEDLINE records

**Global Index Medicus – Results 1539.**

**Restricted to Western Pacific (WPRIM), Eastern Mediterranean (IMEMR), South-East Asian (IMSEAR) and Africa (AIM) Regions and the WHO library (WHOLIS) and 2005-2016.**

Search in title, abstract, subject

1. Maternal text
2. Sepsis text
3. Prevalence text
4. 1 and 2 and 3

**Maternal Text**

| matern* OR pregnan* OR childbirth OR intrapartum OR intra-partum OR postpartum OR post-partum OR postnatal OR puerperal OR puerperium OR parturition OR obstetric OR labo*r OR partum OR deliver* OR perineal OR perineum OR caesarean |
| --- |

**Sepsis Text**

| sepsis OR septic OR septicem* OR septicaem* OR endometritis OR metritis OR endomyometritis OR endoparametritis OR amnionitis OR placentitis OR membranitis OR infect* OR cervicitis OR vaginitis OR "organ failure" |
| --- |

**Prevalence Text**

| prevalence OR proportion OR percent* OR frequency OR incidence OR rate* OR cohort OR "longitudinal study" OR "follow-up study" OR "prospective study" OR "retrospective study" OR cross-sectional OR "intervention study" OR trial OR "community-based study" OR "population-based study" OR "observational study" OR evaluat* OR audit OR epidemiology |
| --- |

**POPLINE – Results 539**

**Restricted to 2005-2016**

| "matern* sepsis" ~5 OR "pregnancy sepsis" ~5 OR "childbirth sepsis" ~5 OR "intrapartum sepsis" ~5 OR "intra-partum sepsis" ~5 OR "puerperal sepsis" ~5 OR "postpartum sepsis" ~5 OR "post-partum sepsis" ~5 OR "postnatal sepsis" ~5 OR "puerperium sepsis" ~5 OR "parturition sepsis" ~5 OR "obstetric sepsis" ~5 OR "labor sepsis" ~5 OR "labour sepsis" ~5 OR "deliver* sepsis" ~5 OR "matern* infection*" ~5 OR "pregnancy infection*" ~5 OR "childbirth infection*" ~5 OR "intrapartum infection*" ~5 OR "intra-partum infection*" ~5 OR "puerperal infection*" ~5 OR "postpartum infection*" ~5 OR "post-partum infection*" ~5 OR "postnatal infection*" ~5 OR "puerperium infection*" ~5 OR "parturition infection*" ~5 OR "obstetric infection*" ~5 OR "labor infection*" ~5 OR "labour infection*" ~5 OR "deliver* infection*" ~5 OR "perineal infection*" ~5 OR "perineum infection*" ~5 OR "caesarean infection*" ~5 OR "puerperal fever" OR "childbed fever" OR "postpartum fever" OR "post-partum fever" OR "puerperal pyrexia" OR "postpartum pyrexia" OR "post-partum pyrexia" OR "puerperal peritonitis" OR chorioamnionitis OR endometritis |
| --- |

**Africa Wide Information – Results 3067**

**Restricted to 2005-Current**

| (matern* or pregnan* or childbirth or intrapartum or intra-partum or postpartum or post-partum or postnatal or puerperal or puerperium or parturition or obstetric or labo*r or partum or deliver* or perineal or perineum or caesarean) N5 (sepsis or septic or septic?em* or endometritis or metritis or endomyometritis or endoparametritis or amnionitis or placentitis or membranitis or infect* or pyrexi* or cervicitis or vaginitis or organ failure or chorioamnionitis or puerperal fever or childbed or puerperal peritonitis or Chorioamnionitis+ or puerperal infection+ ) |
| --- |

**LILACS – Results 1955**

| Matern? Or Embaraz? Or parto or alumbramiento or nacimiento or intraparto or postparto or postnatal or puerperal or puerperio or trabajo de parto or perineo or perineum or cesárea  AND  Sepsis or séptico or septicemia or endometritis or parametritis or amnionitis or infección or fiebre or cervicitis or vaginitis or falla sistémica or corioanmionitis or fiebre puerperal |
| --- |

#

# S2 Text: Data Extraction Form

| **#** | **Question** | **Response codes** | |
| --- | --- | --- | --- |
| 1 | Language of paper | | (1) English  (2) French  (3) German  (4) Spanish  (5) Portuguese  (6) Chinese  (7) Russian  (8) Other  Specify____________________________ |
|  | **STUDY POPULATION** | | |
| 2 | Study Period | | Month/Year Month/Year  \|____\|___\| \|____\|___\| to \|____\|___\| \|____\|___\| |
| 3 | Countries included | |  |
| 4 | Number of study sites included (within and across countries) | |  |
| 5 | Which category(ies) best describes the study population at the study sites? | | (1) Rural  (2) Urban  (3) Periurban/slum  (4) Population not well described |
| 6 | Where were women recruited from? | | (1) Community  (2) Health centre  (3) Hospital  (4) Other  Specify________________________________ |
| 7 | When were women recruited? | | 1. During pregnancy 2. After PROM   (3) During delivery  (4) Postpartum |
| 8 | If recruited at ANC, what percentage of women attend ANC in the study population? | |  |
| 9 | If recruited at delivery, what percentage of women attend for facility delivery in the study population? | |  |
| 10 | Place of delivery (select all that apply) | | (1) Home  (2) BEmONC centre  (3) CEmONC centre (Caesarean section provided)  (3) Unknown  (4) Other  Specify________________________________ |
| 11 | Was a particular subgroup of women studied | | 1. None 2. Caesarean section 3. Diabetes 4. Obesity 5. Pre-term PROM 6. PROM at term 7. Preterm labour/delivery 8. Induction of labour 9. HIV 10. Other   Specify__________________________   1. Other   Specify__________________________ |
| 12 | Was the whole study sample comprised of women from this subgroup? | | 1. Yes 2. No 3. N/A |
| 13 | What proportion of the total population of pregnant women are in this subgroup? | |  |
| 14 | Any other remarks on Study Population | |  |
|  | **STUDY DESIGN AND SAMPLING** | |  |
| 15 | Study design | | (1) Cross-sectional  (2) Cohort/Longitudinal  (3) Controlled Trial  (4) Incidence/Prevalence Survey  (5) Unknown/unclear  (6) Other  Specify____________________________ |
| 16 | Sampling | | (1) Random sample  Specify the method of randomization  ______________________________  (2) Non-random sample  Specify the method of sampling  ______________________________  (3) Total population (i.e. census or all admissions)  (4) Unknown/unclear  (5) Other  Specify_______________________ |
| 17 | Exclusion Criteria | |  |
| 18 | Of those sampled, how many women refused to take part or did not respond? | |  |
| 19 | Were refusers different to those taking part in the study? | | 1. Yes 2. No 3. Unknown |
| 20 | Total number enrolled in the study | |  |
| 22 | Number of the study subjects lost to follow-up (or those not included in the final analysis for cross-sectional designs and RCTs) | |  |
| 23 | Are the characteristics of the study subjects who refused or were lost to follow-up different from the rest of the population? | | (1) YES (2) NO (3) NK |
| 24 | Any other remarks on Design and Sampling | |  |
|  | **STUDY OUTCOME** | |  |
| 25 | What is the definition of sepsis/infection used in this study? | |  |
| 26 | What was the denominator | | 1. Pregnancies 2. Women delivered 3. Live births 4. Live and still births (combined) 5. Unknown/unclear 6. Other   Specify____________________________ |
| 27 | When did follow-up for infection start? | | 1. Antepartum 2. Rupture of membranes 3. Onset of labour 4. Postpartum (specify day)__________ 5. Unknown/unclear |
| 28 | When did follow-up end? | | 1. Antepartum 2. Intrapartum 3. Postpartum (specify day)__________ 4. Unknown/unclear |
| 29 | Is infection the primary outcome of the study? | | (1) YES (2) NO (3) NK |
| 30 | Were other outcomes studied? | | (1) YES (2) NO (3) NK |
| 31 | Was Maternal infection the exposure in the study? | | (1) YES (2) NO |
| 32 | If yes, what was the outcome? | |  |
| 33 | What data source was used to establish the outcome of infection for the study? | | (1) Medical Record  (2) Special Survey/Interview  (3) Clinical data collected for the study  (4) Unknown/unclear  (5) Other  Specify______________________________ |
| 34 | Where was the woman assessed to establish the outcome of infection? | | (1) Home  (2) Health centre  (3) Hospital  (4) Unknown/unclear  (5) Other  Specify______________________________ |
| 35 | Who diagnosed/identified the infection? | | (1) Doctor/clinician  (2) Nurse/midwife  (3) Other trained health provider  (4) Lay/community worker  (5) Unknown/unclear  (5) Other  Specify______________________________ |
| 36 | Was active surveillance used to identify women with infection postpartum | | (1) YES (2) NO |
| 37 | If **yes,** describe the method used | |  |
| 38 | Any other remarks on study outcome | |  |
| 39 | Any other comments | |  |

| **MATERNAL Infection** | | |  |
| --- | --- | --- | --- |
| Incidence | | |  |
| (i) | (ii) | (iii) | (iv) |
| Outcome studied | No of cases (numerator) | Total deliveries/live births (denominator) | Proportion of women with infection |
|  |  |  |  |
|  |  |  |  |
|  |  |  |  |
|  |  |  |  |

# S3: ICD Codes for infection outcomes

| **Outcome** | | **ICD-9** | **ICD-10** |
| --- | --- | --- | --- |
| Chorioamnionitis | | 658.4, 659.2, 762.7 | O41.12 |
| Endometritis | | 670.1 | O86.12 |
| Wound infection | | 674.3 – But no studies specified ICD codes | |
| Sepsis | SIRS* (including puerperal sepsis) | 670.2, 995.91 | O85 |
|  | Severe Sepsis | 995.92, 785.52 | R65.20, R65.21 |
|  | Bacteraemia/Septicaemia | 038, 659.3, 790.7 | R78.81, A40, A41 |
| Peripartum infection | | 670 | O86 |
|  |  | Plus a combination of the codes above | |

# *Systemic inflammatory response syndrome

# S4: Tables of Studies

**Table 1: Studies of Chorioamnionitis**

| **Author** | **Date** | **Country** | **Description** | **Total women** | **Chorioamnionitis**  **(%)** | **Quality** | **Not in meta-analysis** |
| --- | --- | --- | --- | --- | --- | --- | --- |
| Abramovici (2014)^1^ | 11/08-06/10 | US | Chorioamnionitis extracted from medical records of women in a single-hospital RCT of different oxytocin doses. Low-risk women with vaginal delivery and livebirth at one hospital | 1785 | 6.78 | 4 |  |
| Admaty (2012)^2^ | 03/09-12/10 | Switzerland | Signs of chorioamnionitis extracted from maternal medical records for a study of newborn outcomes at different gestational ages in 2 hospital. Term births only | 143 | 0.70 | 2 |  |
| Al-Ostad (2015)^3^ | 01/98-12/08 | US | Study of risk factors for sepsis using National Inpatient Sample (NIS) data representing all hospital deliveries in the US | 5338995 | 1.73 | 5 |  |
| Bear (2016)^4^ | 01/91-12/01 | US | Medical record discharge diagnosis at all non-federal hospitals in California for a study of cerebral palsy and maternal infection | 6018504 | 1.84 | 4 |  |
| Berg (2009)^5^ | 01/01-12/05 | US | Study of maternal morbidity during hospitalisation for labour using the National Hospital Discharge Survey representing all hospital deliveries in the US. | 19986000* | 1.50 | 4 | Overlapping data |
| Berg (2009)^5^ | 01/93-12/97 | US | As above | 19081000* | 1.90 | 4 |  |
| Bleich (2012)^6^ | 01/03-12/08 | US | Medical record data on chorioamnionitis from a study of duration of second stage of labour. Women with live births at 1 hospital | 21991 | 19.66 | 4 | Outlier |
| Borders (2012)^7^ | 2009 | US | Audit of number of vaginal examinations in labour and routine midwife diagnosis of chorioamnionitis. Term deliveries at one hospital | 205 | 6.34 | 3 |  |
| Braun (2016)^8^ | 01/10-12/10 | US | Study of perinatal sepsis in term infants at 13 hospitals in the Kaiser Permanent Medical Program (KPMP), California, and integrated managed care consortium. Medical record data on chorioamnionitis. | 31112 | 4.00 | 5 |  |
| Caughey (2007)^9^ | 01/95-12/99 | US | Study of maternal complications at 13 KPMP facilities. Medical record data of low-risk, term deliveries | 119254 | 3.49 | 5 |  |
| Cavazos-Rehg (2015)^10^ | 01/09-12/09 | US | Study of maternal age and delivery complications using NIS data | 4109295 | 1.67 | 5 | Overlapping data |
| Cheng (2007)^11^ | 01/91-12/02 | US | Medical record data on chorioamnionitis from a study of maternal and newborn outcomes by duration of second stage of labour. Multiparous women with livebirths at term in one hospital. | 5158 | 4.28 | 4 |  |
| Cheng (2010)^12^ | 01/90-07/08 | US | Signs of chorioamnionitis extracted from medical records from a study of perinatal outcomes by duration of first stage of labour. Nulliparous women with live, term births at 1 hospital. | 10661 | 12.56 | 5 |  |
| Danilack (2015)^13^ | 01/11-12/13 | US | Chorioamnionitis on birth certificates of all low-risk women delivering in the US | 10458616 | 1.29 | 2 |  |
| Dotters-Katz (2015)^14^ | 01/08-12/10 | US | Study of infection in multiple versus single gestation using NIS data | 12524118* | 2.58 | 5 |  |
| Edwards (2015)^15^ | 06/06-11/07 | US | Signs of chorioamnionitis extracted from maternal medical records for a study of an early warning system for severe sepsis at one hospital. | 15027 | 6.08 | 5 |  |
| Geller (2010)^16^ | 1995-2005 | US | Intrapartum fever extracted from medical records for study of maternal outcomes and planned mode of birth at one hospital. Low-risk, nulliparous women delivering at term | 4048 | 15.74 | 4 |  |
| Getahun (2010)^17^ | 01/91-12/07 | US | Medical record data for study of effect of chorioamnionitis on childhood asthma at KPMP hospitals. Only includes infants who became health plan members. | 397852 | 3.20 | 3 | Overlapping data |
| Getahun (2013)^18^ | 01/95-12/10 | US | Medical record data of temporal trends in chorioamnionitis in KPMP hospitals. | 471821 | 4.12 | 4 |  |
| Grotegut (2008)^19^ | 01/03-06/05 | US | Medical record data on obstetric outcomes with false-positive glucose challenge test (GCT) at 1 hospital. Normal GCT only | 165 | 0.61 | 4 |  |
| King (2012)^20^ | 08/95-02/04 | US | Maternal and Neonatal morbidity using the perinatal database at 1 hospital. Live births at term. | 14406 | 12.85 | 4 |  |
| Magann (2008)^21^ | 03/04-02/05 | US | Obstetric characteristics for prolonged third stage of labour. Source of data unclear. Vaginal deliveries at a naval medical centre. | 1607 | 2.18 | 4 |  |
| Malloy (2014)^22^ | 01/08-12/08 | US | Birth certificate data for study of chorioamnionitis and newborn outcomes. Live, term births across the US | 2224406 | 0.99 | 4 | Overlapping data |
| Matsuda (2011)^23^ | 2001-2005 | Japan | Data from perinatal registry network of 125 centres. | 242715 | 1.03 | 4 |  |
| Nelson (2014)^24^ | 01/05-12/11 | US | Study of obstetric risk factors for newborn complications. Source of data unclear. Live, term births at 1 hospital | 86371 | 6.61 | 4 |  |
| Osmundson (2011)^25^ | 07/06-06/08 | US | Medical record data on chorioamnionitis for a sample of low-risk women managed expectantly (not induced) at 39 weeks gestation in 1 hospital | 102 | 19.61 | 3 |  |
| Shah (2011)^26^ | 09/08-11/08 | Pakistan | Medical record data on obstetric outcomes of low-risk women at 3 hospitals. Convenience sample of women aged 20-35 | 916 | 0.76 | 2 |  |
| Suthee (2007)^27^ | 01/99-12/03 | Thailand | Signs of chorioamnionitis extracted from medical records in study of meconium-stained amniotic fluid and maternal infection. Low-risk women with live, term birth at 1 hospital | 1079 | 0.93 | 5 |  |

*Results presented are weighted percentage of US population. In meta-analysis we approximated the sample size at 20% for the NIS^28^ and 1% for the NHDS.^29^

**Table 2: Studies of Endometritis**

| **Author** | **Date** | **Country** | **Description** | **Total women** | **Endometritis (%)** | **Quality** | **Not in meta-analysis** |
| --- | --- | --- | --- | --- | --- | --- | --- |
| Ahnfeldt-Mollerup (2012)^30^ | 05/07-04/08 | Denmark | Questionnaire sent to women 28 days after delivering at 1 regional hospital. Report of infection validated with data from General Practice and hospital records. | 1616 | 1.86 | 2 |  |
| Ayzac (2008)^31^ | 01/97-12/03 | France | Clinical endometritis after vaginal delivery until 30 days postpartum at 66 hospitals in a surveillance network. | 161077 | 0.33 | 5 |  |
| Belfort (2010)^32^ | 01/07-12/07 | US | Women readmitted with clinical uterine infection up to 42 days postpartum. Medical record data from 114 hospitals representative of the US population. | 222751 | 0.15 | 4 |  |
| Benincasa (2012)^33^ | 01/04-12/10 | Brazil | Medical record data on clinical puerperal infection at 1 hospital | 26691 | 1.47 | 3 |  |
| Bianco (2013)^34^ | 09/07-09/08 | Italy | Telephone calls with women at 30 days after delivery at 1 hospital. Postpartum infections corroborated by hospital and physician visits, wound cultures and antibiotic prescriptions. | 1656 | 1.39 | 3 |  |
| Boccardo (2013)^35^ | 04/10-07/10 | Argentina | Medical record data on clinical endometritis in 1 public hospital. | 1472 | 2.51 | 5 |  |
| Caughey (2007)^9^ | 01/95-12/99 | US | Maternal complications by gestational age. Medical record data on endometritis at 13 Californian hospitals in an insurance programme (KPMP) | 119254 | 1.20 | 5 |  |
| Cavazos-Rehg (2015)^10^ | 01/09-12/09 | US | Maternal age and delivery complications using NIS data | 4109295 | 0.36 | 4 | Overlapping data |
| Cheng (2007)^11^ | 01/91-12/02 | US | Maternal and newborn outcomes by duration of 2nd stage of labour in multiparous women. Medical record data at 1 hospital | 5158 | 1.36 | 4 |  |
| Cheng (2010)^12^ | 01/90-07/08 | US | Perinatal outcomes by duration of 1st-stage of labour in nulliparous women. Medical record data at 1 hospital | 10661 | 2.37 | 5 |  |
| Chongsuvivatwong (2010)^36^ | 09/01-09/04 | 9 Asian countries | Clinical data on maternal and foetal complications collected by checklist until day 5 postpartum in 12 teaching hospitals in Asia. Vaginal deliveries only. | 12591 | 0.06 | 2 |  |
| Darmstadt (2009)^37^ | 06/01-07/01 | Egypt | Study of clean delivery-kit use in 1 urban and 2 rural areas. Infection diagnosed by nurse at week 1 postnatal home visit | 334 | 1.50 | 4 |  |
| Dimitriu (2010)^38^ | 1/1/06-1/9/09 | Kuwait | Medical record data of puerperal infection at 1 hospital | 7550 | 1.63 | 2 |  |
| Dotters-Katz (2015)^14^ | 01/08-12/10 | US | Endometritis in single and multiple gestation using NIS data | 12524118* | 1.36 | 5 |  |
| Dumas (2008)^39^ | 01/01-12/04 | France | Clinical endometritis after vaginal delivery until 30 days postpartum at 44 hospitals in a surveillance network. | 49786 | 0.23 | 4 | Overlapping data |
| Ezugwu (2011)^40^ | 09/08-12/08 | Nigeria | Medical record data on obstetric outcomes, including genital sepsis, at 1 hospital during the period of free maternal care. | 1152 | 1.74 | 1 |  |
| Fronczak (2005)^41^ | 11/93-05/95 | Bangladesh | Multi-stage probability sampling of women in slum areas of Dhaka. Pelvic infection identified at interviews conducted at home at 72 hours, 7 days and, with examination by a doctor, 14-22 days postpartum. | 1506 | 14.01 | 3 | Outlier |
| Geller (2010)^16^ | 1995 -2005 | US | Medical record data on maternal outcomes and planned mode of birth among nulliparous, low-risk women at 1 hospital. | 4048 | 1.31 | 4 |  |
| Ghani (2007)^42^ | 1/7/05-31/7/05 | Pakistan | Self-reported symptoms of vaginal infection during interview at home by trained nurse/midwife. Simple random sample of postpartum women in the Khyber Agency | 1000 | 16.20 | 3 | Outlier |
| Gozum (2005)^43^ | 05/00-06/00 | Turkey | Vaginal infection until 6 weeks postpartum, reported during interviews with mothers attending for 2 month infant immunisations at 1 primary care unit | 112 | 14.29 | 1 | Outlier |
| Grotegut (2008)^19^ | 01/03-06/05 | US | Medical record data on obstetric outcomes with false-positive glucose challenge test (GCT) at 1 hospital. Normal GCT only | 165 | 1.21 | 4 |  |
| Guimaraes (2007)^44^ | 12/00-07/03 | Brazil | Puerperal infection among women at 1 maternity hospital, followed until 30 days postpartum using the National Nosocomial Infection Surveillance System. | 5178 | 0.89 | 4 |  |
| Ivanov (2014)^45^ | 01/11-12/13 | Bulgaria | Medical record data on puerperal infection at 1 hospital. | 7181 | 9.89 | 3 |  |
| Iyengar (2012)^46^ | 01/07-12/10 | India | A field site in rural Rajasthan. Clinical uterine infection diagnosed during home visits by trained nurse-midwives at 2-3 days and 6-9 days postpartum. | 4975 | 1.29 | 4 |  |
| Jokhio (2005)^47^ | 05/98-10/98 | Pakistan | Cluster RCT of traditional birth attendant (TBA) training in Larkana District. Lady Health Workers were trained to recognise complications during their routine monthly visits. Women with trained TBA | 9838 | 0.79 | 3 |  |
| Jokhio (2005)^47^ | As above | As above | As above; women without trained TBA | 9119 | 4.39 | 3 |  |
| King (2012)^20^ | 08/95-02/04 | US | Maternal and Neonatal morbidity using the perinatal database at 1 hospital | 14335 | 2.53 | 5 |  |
| Kovavisarach (2005)^48^ | 11/01-02/02 | Thailand | RCT of perineal shaving vs hair cutting on maternal and neonatal outcomes among low-risk women with vaginal delivery at 1 hospital | 458 | 0.00 | 3 |  |
| Magann (2011)^49^ | 01/07-07/08 | US | Medical record data on obesity and peripartum complications at 2 hospitals | 4490 | 6.88 | 4 |  |
| Maric (2006)^50^ | 1/04-12/04 | Bosnia | Medical record data on puerperal complications until 42 days postpartum in nulliparous women at 1 hospital. Vaginal deliveries | 119 | 1.68 | 2 |  |
| Ngoga (2009)^51^ | Start 12/03 | South Africa | Medical record data on pregnancy outcomes in morbidly obese vs a matched sample of normal weight women at 1 hospital. Women with BMI 20-25 | 209 | 0.48 | 2 |  |
| Sanchez (2015)^52^ | 01/12-12/13 | Cuba | Maternal age and obstetric complications using medical record data at 1 hospital. Each month, first 30 women aged 25-30 enrolled. | 720 | 1.67 | 2 |  |
| Sanchez (2015)^52^ | As above | As above | As above. Each month, the first 15 women over 35 enrolled | 360 | 2.22 | 2 |  |
| Suthee (2007)^27^ | 01/99-12/03 | Thailand | Medical record data on meconium-stained amnionitic fluid and maternal infection among low-risk women at 1 hospital | 1079 | 0.93 | 4 |  |
| Peret (2007)^53^ | 07/01-09/03 | Brazil | Puerperal morbidity in HIV-infected vs pair-matched non-infected women at 1 hospital; diagnosed before discharge and at a scheduled visit with researchers at 7-15 days postpartum. HIV negative women | 123 | 0.00 | 3 |  |
| Ramírez-Villalobos (2009)^54^ | 04/03-12/03 | Mexico | Puerperal complications after hospital discharge among women with vaginal delivery at 1 hospital. Self-reported symptoms collected by trained interviewers at a clinic or home visit at day 7 postpartum | 302 | 2.65 | 3 |  |
| Saizonou (2014)^55^ | 07/09-02/10 | Benin | Peripartum infection up to 7 days postpartum at 1 hospital. Diagnosed by doctor or midwife supervised by public health doctor | 1875 | 1.60 | 4 |  |
| Sanabria (2011)^56^ | 01/07-12/09 | Cuba | Medical record data on puerperal complications at 1 hospital | 5645 | 0.48 | 1 |  |
| Tabcharoen (2009)^57^ | 01/97-12/06 | Thailand | Medical record data on pregnancy outcomes after age 40 at 1 hospital. Women aged 20-34 | 20852 | 0.10 | 4 |  |
| Tabcharoen (2009)^57^ | As above | As above | As above; women age 40+ | 792 | 0.38 | 4 |  |
| Winani (2007)^58^ | Start 01/2000 | Tanzania | Cord infection and puerperal sepsis with clean delivery kits in 2 rural districts. Home visit at day 5 by village health workers with suspected infection confirmed at health facility | 3262 | 2.12 | 4 |  |

*Results presented are weighted percentage of US population. In meta-analysis we approximated the sample size at 20% for the NIS.^28^

**Table 3: Studies of Wound infection**

| **Author** | **Date** | **Country** | **Description** | **Total women** | **Wound Infection**  **(%)** | **Quality** |
| --- | --- | --- | --- | --- | --- | --- |
| Ahnfeldt-Mollerup (2012)^30^ | 05/07-04/08 | Denmark | Questionnaire sent to women 28 days after delivering at 1 regional hospital. Report of infection validated with data from General Practice and hospital records. | 1616 | 3.16 | 2 |
| Awan (2015)^59^ | 10/10-09/11 | Pakistan | Feto-maternal outcomes in overweight versus normal weight in 1 hospital. Data source unclear. Results for normal weight (18.5-24.9) | 100 | 2.00 | 0 |
| Bailit (2006)^60^ | 01/01-12/01 | US | Study of quality of obstetric care. Birth certificate record data from California | 431125 | 0.20 | 4 |
| Bianco (2013)^34^ | 09/07-09/08 | Italy | Telephone calls with women at 30 days after delivery at 1 hospital. Postpartum infections corroborated by hospital and physician visits, wound cultures and antibiotic prescriptions. | 1656 | 3.08 | 3 |
| Bodner (2011)^61^ | 11/05-01/09 | Austria | Maternal and neonatal outcomes for elective caesarean and planned vaginal delivery. Data source unclear. Low-risk women at 1 hospital. Planned vaginal deliveries only | 178 | 1.12 | 2 |
| Charrier (2010)^62^ | 05/04-10/04 | Italy | Study of clean versus sterile vaginal delivery at 2 hospitals. Signs of perineal infection in hospital from direct observation and medical records. Telephone interview at 20-30 days postpartum for reported infection diagnosis, symptoms and antibiotic use. | 409 | 0.00 | 4 |
| Chongsuvivatwong (2010)^36^ | 09/01-09/04 | 9 Asian countries | Clinical data on maternal and foetal complications collected by checklist until day 5 postpartum in 12 teaching hospitals in Asia. Vaginal deliveries only. | 12591 | 2.57 | 1 |
| Danish (2010)^63^ | 05/98-11/99 | Pakistan | Pregnancy outcome in booked versus unbooked women at 1 hospital. Data collection poorly described. | 322 | 6.21 | 0 |
| Dasgupta (2014)^64^ | 10/10-09/11 | India | Pregnancy outcomes in obesity at 1 hospital. Data source unclear. Results for normal BMI (<25kg/m2) | 99 | 2.02 | 1 |
| Dimitriu (2010)^38^ | 01/06-09/09 | Kuwait | Medical record data of puerperal infection at 1 hospital | 7550 | 0.33 | 2 |
| Dong (2009)^65^ | 01/01-11/04 | China | Before-after study of infection prevention control intervention at 1 hospital. Medical record data of perineal and caesarean wound infections in the control group | 12850 | 1.32 | 4 |
| Dong (2010)^66^ | 07/08-08/08 | China | Controlled trial of hand washing method for vaginal deliveries at 1 hospital. Perineal infection data collected by the study doctor | 300 | 8.67 | 4 |
| Ezugwu (2011)^40^ | 09/08-12/08 | Nigeria | Medical record data on obstetric outcomes, including wound sepsis, at 1 hospital during the period of free maternal care. | 1152 | 8.33 | 1 |
| Geller (2010)^16^ | 1995-2005 | US | Medical record data on maternal outcomes and planned mode of birth among nulliparous, low-risk women at 1 hospital. | 4048 | 0.02 | 4 |
| Goff (2013)^67^ | 01/08-12/09 | US | Medical record data from the Perspective database; 355 hospitals accounting for approximately 20% of all hospital admission in the US | 1001189 | 0.35 | 4 |
| Guimaraes (2007)^44^ | 12/00-07/03 | Brazil | Surgical site and episiotomy infection among women at 1 maternity hospital, followed until 30 days postpartum using the National Nosocomial Infection Surveillance System. | 5178 | 1.95 | 4 |
| Ivanov (2014)^45^ | 01/11-12/13 | Bulgaria | Medical record data on puerperal infection at 1 hospital. Results for perineal wound infection after vaginal delivery | 3897 | 4.29 | 3 |
| Iyengar (2012)^46^ | 01/07-12/10 | India | A field site in rural Rajasthan. Perineal wound infection diagnosed during home visits by trained nurse-midwives at 2-3 days and 6-9 days postpartum. | 4975 | 0.42 | 4 |
| Jaleel (2009)^68^ | 01/06-04/08 | Pakistan | Pregnancy outcomes in obesity at 1 private maternity home. Data source unclear. Results for control group (BMI 18.5-22.9) | 118 | 0.00 | 1 |
| Janssen (2009)^69^ | 01/00-12/04 | Canada | Medical record data. Low risk women in British Colombia planning to delivery with a midwife at home or hospital | 7641 | 0.14 | 3 |
| Janssen (2009)^69^ | 01/00-12/05 | Canada | As above. Low risk women planning to delivery with a physician in hospital | 5331 | 0.30 | 3 |
| Kovavisarach (2005)^48^ | 11/01-02/02 | Thailand | RCT of perineal shaving versus hair cutting on maternal and neonatal outcomes in low-risk women with vaginal delivery at 1 hospital. Perineal wound infection. Unclear if up to day 4 or 42 | 458 | 8.73 | 3 |
| Latif (2013)^70^ | 01/00-06/00 | Bangladesh | Medical record data of outcomes in primigravidae at 1 hospital | 500 | 3.00 | 3 |
| Leth (2009)^71^ | 01/01-12/05 | Denmark | Wound infection up to 30 days postpartum identified through the laboratory system, regional prescription database and National Hospital Registry. All deliveries in County of Aarhus | 32468 | 1.78 | 4 |
| Liu (2010)^72^ | 01/05-12/06 | China | Clinical study data on abdominal and perineal wound infection and body mass index at 1 hospital. Results for BMI<25 | 327 | 8.87 | 3 |
| Ngoga (2009)^51^ | 12/03 | South Africa | Medical record data on pregnancy outcomes in morbidly obese vs a matched sample of normal weight women at 1 hospital. Women with BMI 20-25 | 209 | 0.00 | 2 |
| Oladapo (2007)^73^ | 01/90-12/05 | Nigeria | Medical record data on wound infection. Vaginal deliveries at 1 hospital | 656 | 5.18 | 3 |
| Petter (2013)^74^ | 01/09-12/10 | Brazil | Medical record data on episiotomy and caesarean wound infections among women at 1 hospital | 9528 | 1.24 | 5 |
| Ramírez-Villalobos (2009)^54^ | 04/03-12/03 | Mexico | Episiotomy infection after hospital discharge among women with vaginal delivery at 1 hospital. Self-reported symptoms collected by trained interviewers at a clinic or home visit at day 7 postpartum | 303 | 10.89 | 3 |
| Shriraam (2012)^75^ | 11/08-02/09 | India | Self-reported wound infection up to 42 days postpartum using pre-tested questionnaire at up to 6 months after delivery. All women delivered in previous 6 months in rural community of Tamil Nadu | 365 | 2.74 | 2 |

**Table 4: Studies of Sepsis**

| **Author** | **Date** | **Country** | **Description** | **Total women** | **Sepsis (%)** | **Quality** | **Not in meta-analysis** |
| --- | --- | --- | --- | --- | --- | --- | --- |
| Acosta (2013)^76^ | 01/05-12/07 | US | Medical record data for all admissions for delivery of a live birth in California. Sepsis coded as septicaemia or sepsis | 1622474 | 0.10 | 5 |  |
| Acosta (2013)^76^ | 01/05-12/07 | US | As above. Severe sepsis, also coded as septic shock or sepsis with prolonged length of stay, transfer to intensive care or death. | 1622474 | 0.05 | 5 |  |
| Bauer (2013)^77^ | 01/98-12/08 | US | Maternal sepsis during hospitalisation for delivery using NIS data. Sepsis coded as septicaemia or SIRS | 8999852* | 0.03 | 5 |  |
| Bauer (2013)^77^ | 01/98-12/08 | US | As above. Severe sepsis coded as sepsis plus organ dysfunction | 8999852* | 0.01 | 5 |  |
| Belfort (2010)^32^ | 01/07-12/07 | US | Medical record data on women readmitted with postpartum infection up to 42 days postpartum at 114 hospitals, representative of the US population. | 222751 | 0.01 | 3 | Overlapping data |
| Ben (2007)^78^ | 01/99-12/03 | Tunisia | Medical record data on all severe (near-miss) puerperal infection at one hospital using SIRS criteria. | 20071 | 0.08 | 4 |  |
| Callaghan (2008)^79^ | 01/91-12/03 | US | Septicaemia and hospital stay of 3+ days using data on delivery hospitalisations from the National Hospital Discharge Survey | 423480 | 0.02 | 5 |  |
| Cape (2013)^80^ | 01/00-12/08 | US | Bacteraemia from 7 days before until 30 days after delivery using the microbiology database at one hospital. Restricted to women with a diagnosis of chorioamnionitis, endometritis or wound infection | 78919 | 0.17 | 4 |  |
| Chongsuvivatwong (2010)^36^ | 09/01-09/04 | 9 Asian countries | Clinical data on maternal and foetal complications including peritonitis, collected by checklist until day 5 postpartum in 12 teaching hospitals in Asia. Vaginal deliveries only. | 12591 | 0.02 | 1 |  |
| David (2012)^81^ | 01/05-12/10 | India | Medical record data on puerperal sepsis during hospitalisation for delivery, in a midwife-run labour room at one urban health centre. | 1194 | 0 | 2 |  |
| Dotters-Katz (2015)^14^ | 01/08-12/10 | US | Study of infection in multiple versus single gestation using NIS data. Codes for septicaemia and bacteraemia | 12524118* | 0.07 | 5 |  |
| Goff (2013)^67^ | 01/08-12/09 | US | Medical record data from the Perspective database; 355 hospitals accounting for approximately 20% of all hospital admission in the US. Codes for septicaemia, septic shock, bacteraemia, SIRS | 1001189 | 0.13 | 5 | Overlapping data |
| Huda (2012)^82^ | 01/08-12/08 | Bangladesh | Medical record data from 30 hospitals on genital infection and signs of shock, from labour until 32 days postpartum | 1927 | 0.88 | 4 |  |
| Ivanov (2014)^45^ | 01/11-12/13 | Bulgaria | Medical record data on puerperal infection, including sepsis, at 1 hospital. | 7181 | 0.08 | 3 |  |
| Karolinski (2013)^83^ | 06/08-05/09 | Argentina | Medical record data from 25 hospitals in the Perinatal network of Buenos Aires on life-threatening puerperal sepsis until 42 days postpartum | 65033 | 0.04 | 3 |  |
| Knowles (2014)^84^ | 01/05-12/12 | Ireland | Medical and laboratory records at 2 maternity hospitals of blood stream infection secondary to genital tract infection until 42 days postpartum | 136897 | 0.11 | 5 |  |
| Kuklina (2008)^85^ | 01/98-12/04 | US | Sepsis coded as septicaemia, septic shock or SIRS with/without organ dysfunction during hospitalisation for delivery using NIS data | 28084407 | 0.03 | 5 | Overlapping data |
| Leth (2009)^71^ | 01/01-12/05 | Denmark | Blood stream infection up to 30 days postpartum identified through the laboratory system, regional prescription database and National Hospital Registry. All deliveries in County of Aarhus | 32468 | 0.06 | 5 |  |
| Luz (2008)^86^ | 10/05-07/06 | Brazil | Positive blood culture and SIRS or organ dysfunction, collected from medical records during admission for delivery at one hospital | 2207 | 0.05 | 5 |  |
| Lyndon (2012)^87^ | 01/05-12/07 | US | Medical record data of maternal sepsis from all live singleton births at hospitals in California. | 1572909 | 0.09 | 4 | Overlapping data |
| Maric (2006)^50^ | 01/04-12/04 | Bosnia | Medical record data on puerperal sepsis following vaginal delivery until 42 days postpartum in nulliparous women at 1 hospital | 119 | 0 | 2 |  |
| Mayi-Tsonga (2007)^88^ | 06/06-12/06 | Gabon | Audit of near-miss at one hospital. Medical record data on septic shock of pelvic origins | 4350 | 0 | 5 |  |
| Pallasmaa (2008)^89^ | 01/97-12/97 | Finland | Puerperal sepsis and peritonitis in all singleton births in Finland using the national hospital discharge registry | 57149 | 0.33 | 4 |  |
| Pallasmaa (2008)^89^ | 01/02-12/02 | Finland | Puerperal sepsis and peritonitis in all singleton births in Finland using the national hospital discharge registry | 53568 | 0.45 | 4 |  |
| Pallasmaa (2015)^90^ | 01/07-12/11 | Finland | Puerperal sepsis, peritonitis and re-operation in all singleton births in Finland using the national hospital discharge registry | 292553 | 0.81 | 4 |  |
| Sanabria (2011)^56^ | 01/07-12/09 | Cuba | Medical record data on puerperal complications including sepsis among women delivering at 1 hospital | 5645 | 0.18 | 1 |  |
| Shriraam (2012)^75^ | 11/08-02-09 | India | Self-reported puerperal sepsis up to 42 days postpartum using pre-tested questionnaire at up to 6 months after delivery. All women delivered in previous 6 months in rural community of Tamil Nadu | 365 | 3.84 | 2 | Outlier |
| Simoes (2005)^91^ | 01/98-12/98 | Germany | Postpartum septicaemia in the Perinatal database for all women delivering in hospitals in Baden-Wurttemberg State. | 103945 | 0.09 | 3 |  |
| Simoes (2005)^91^ | 01/01-12/01 | Germany | Postpartum septicaemia in the Perinatal database for all women delivering in hospitals in Baden-Wurttemberg State. | 88874 | 0.23 | 3 |  |
| Tippawan (2014)^92^ | 10/10-09/11 | Thailand | Medical record data on puerperal sepsis in all hospital deliveries in the country using the National Health Security Office data | 442818 | 0.11 | 5 |  |
| Zhang (2005)^93^ | 01/95-02/98 | 9 European countries | Data collected from medical records on sepsis (infection with SIRS) at the time of birth. Survey usually covered the hospitals in one region of each country for 12 months. | 211264 | 0.07 | 3 |  |

*Results presented are weighted percentage of US population. In meta-analysis we approximated the sample size at 20% for the NIS^28^

**Table 5: Studies of Maternal Peripartum Infection**

| **Author** | **Date** | **Country** | **Description** | **Total Women** | **Maternal Peripartum Infection**  **(%)** | **Quality** | **Not in meta-analysis** |
| --- | --- | --- | --- | --- | --- | --- | --- |
| Al-Ostad (2015)^3^ | 01/98-12/08 | US | Risk factors for sepsis mortality using NIS data. Unspecified codes for puerperal infection. | 5338995 | 0.44 | 4 |  |
| Andersson (2011)^94^ | 05/09-11/09 | Nigeria | Self-reported symptoms of infection up to 42 days postpartum. Stratified random sampling to provide state-level representation for 2 Nigerian states. | 14890 | 18.11 | 1 | Outlier |
| Avci (2015)^95^ | 03/12-03/13 | Turkey | Maternal obesity and perinatal outcomes at one hospital. Definition and data collection methods for postpartum infection not specified. | 931 | 2.36 | 2 |  |
| Bailit (2006)^60^ | 01/01-12/01 | US | Birth certificate record data from California. ICD-9 codes for major postpartum infection, postpartum fever, GU tract infection and wound complications | 431125 | 2.08 | 4 |  |
| Bailit (2013)^96^ | 03/08-02/11 | US | Medical record data from a stratified random selection of days at 25 hospitals in a network of Maternal-Fetal Medicine Units. Peripartum infection in low-risk women defined as Chorioamnionitis, postpartum endometritis or postpartum wound infection. | 110205 | 5.06 | 4 |  |
| Bakr (2005)^97^ | 01/02-06/02 | Egypt | Before-after study of vaginal chlorhexidine intervention and maternal morbidity at one hospital. Medical record data from the pre-intervention period. Postpartum infection defined as puerperal sepsis, or fever plus offensive vaginal discharge, infected wound, retained products of conception or secondary PPH | 2128 | 0.52 | 4 |  |
| Berg (2009)^5^ | 01/01-12/05 | US | Maternal morbidity during hospitalisation for labour using the National Hospital Discharge Survey representing all hospital deliveries in the US. ICD-9 codes for major puerperal infection | 19986000* | 0.50 | 4 | Overlapping data |
| Berg (2009)^5^ | 01/93-12/97 | US | As above | 19081000* | 0.80 | 4 |  |
| Chen (2014)^98^ | 2011 | China | Random sample of 250 medical records of low-risk deliveries at one hospital. Textbook definition of puerperal infection | 250 | 4.00 | 4 |  |
| Dong (2010)^66^ | 07/08-08/08 | China | Controlled trial of hand washing method for low-risk vaginal deliveries at 1 hospital. Data collected by study doctor on puerperal infection (undefined) | 300 | 5.67 | 4 |  |
| Galyean (2009)^99^ | 07/02-12/03 | US | Multiparous women with live singleton delivery at four hospitals in California. Serious post-partum infections requiring aminoglycosides from a perinatal outcomes database | 10654 | 2.87 | 3 |  |
| Gibson (2014)^100^ | 01/02-12/08 | US | Outcomes in elective induction of low-risk pregnancies at 12 clinical centres and 19 hospitals. Medical record data on infection; intrapartum fever, chorioamnionitis, endomyometritis and wound separation | 96266 | 9.06 | 3 |  |
| Goff (2013)^67^ | 01/08-12/09 | US | Medical record data from the Perspective database; 355 hospitals accounting for approximately 20% of all hospital admissions in the US. ICD-9 codes for chorioamnionitis and major puerperal infection | 1001189 | 2.05 | 5 |  |
| Guendelman (2006)^101^ | 01/96-12/98 | US | Database of birth certificate and hospital discharge records for 93% of deliveries in California. ICD-9 codes for major puerperal infection. | 1507275 | 0.90 | 5 |  |
| Harrison (2015)^102^ | 01/10-12/13 | 6 LMICs | 7 rural communities in Argentina, Guatemala, India, Kenya, Pakistan and Zambia, under the Global Network. Undefined postpartum maternal infection from medical records and a study visit at 42 days | 263648 | 0.67 | 3 |  |
| Jin (2011)^103^ | 03/05-03/10 | China | Study of gestational diabetes in one hospital. Undefined puerperal infection collected in a sample of women without diabetes for a single-facility study of gestational diabetes | 192 | 2.08 | 3 |  |
| Karlstrom (2013)^104^ | 01/97-12/06 | Sweden | Register of all facility births in the country. Postpartum infection (undefined) after spontaneous onset of labour at term | 13774 | 1.13 | 4 |  |
| Kovavisarach (2010)^105^ | 11/06-12/07 | Thailand | Women aged 20-34 delivering at one hospital. Puerperal infection with undefined definition or data collection methods. | 750 | 0.13 | 2 |  |
| Kuklina (2008)^85^ | 01/98-12/04 | US | NIS database. ICD-9 codes for puerperal infection and pyrexia of unknown origin. | 28084407* | 0.52 | 5 |  |
| Kyser (2012)^106^ | 01/06-12/06 | US | Medical record data from 1045 hospitals in 11 states. Undefined postpartum infection using ICD-9 codes | 1678809 | 0.72 | 4 | Overlapping data |
| Laws (2014)^107^ | 01/01-12/09 | Australia | Undefined postpartum infection from linked birth records and hospital admission records up to 1 year postpartum. Women intending to deliver at 8 birthing centres | 14707 | 1.04 | 4 |  |
| Laws (2014)^107^ | 01/01-12/09 | Australia | As above. Women intending to deliver at 8 co-located hospitals | 29414 | 1.43 | 3 |  |
| Liu (2007)^108^ | 04/91-03/05 | Canada | Low-risk planned vaginal deliveries at all acute-care hospitals in Canada, excluding Quebec and Manitoba. Medical record data of major puerperal infection from ICD-9 codes. | 2292420 | 0.21 | 5 |  |
| Lyndon (2012)^87^ | 01/05-12/07 | US | Medical record data of livebirths at hospitals in California. Unspecified ICD-9 codes for maternal infection | 1572909 | 2.75 | 4 | Overlapping data |
| Mandal (2010)^109^ | 01/06-12/08 | India | Maternal obesity and pregnancy outcome at one hospital. Combined endometrial and wound infection at 6 weeks postpartum visit in low-risk non-obese women | 422 | 3.79 | 1 |  |
| Ngoc (2005)^110^ | 01/01-07/01 | Vietnam | Clinical data collected at 6-week postpartum study visit after vaginal delivery at two hospitals. Serious postpartum infection defined as physician-diagnosed sepsis or clinical symptoms of endometritis, pelvic abscess, or chorioamnionitis | 978 | 4.81 | 3 |  |
| Okumura (2014)^111^ | 01/00-12/00 | Peru | Perinatal Information System database from one hospital. ICD-10 codes for puerperal infection | 67693 | 2.40 | 4 |  |
| Palmer (2015)^112^ | 04/10-03/12 | UK | Database of all NHS hospital deliveries. ICD-10 codes for puerperal infection or sepsis within 42 days of birth | 1332835 | 0.83 | 5 |  |
| Tippawan (2014)^92^ | 10/10-09/11 | Thailand | Medical record data on puerperal sepsis in all hospital deliveries in the country using the National Health Security Office data. ICD-10 code for other puerperal infection | 442818 | 0.25 | 5 |  |
| Wang (2010)^113^ | 01/07-12/08 | China | Medical record data from one hospital. Postpartum intrauterine infection defined as fever, headache, dizziness, abnormal lochia, genital tract or caesarean wound infection. | 2382 | 5.75 | 5 |  |

*Results presented are weighted percentage of US population. In meta-analysis we approximated the sample size at 20% for the NIS^28^ and 1% for the NHDS.^29^

**S5: Quality of 111 included studies**

| **Yes** |  |
| --- | --- |
| **Unclear** |  |
| **No** |  |

| **Author (date)** | **Sampling** | **Coverage** | **Definition** | **Data collection** | **Sufficient detail** |
| --- | --- | --- | --- | --- | --- |
| Abramovici (2014) | • Yes | • No | • Yes | • Yes | • Yes |
| Acosta (2013) | • Yes | • Yes | • Yes | • Yes | • Yes |
| Admaty (2012) | • No | • Unclear | • Yes | • Yes | • No |
| Ahnfeldt-Mollerup (2012) | • Yes | • Yes | • No | • No | • No |
| Al-Ostad (2015) | • Yes | • Yes | • Yes | • Yes | • Yes |
| Andersson (2011) | • No | • Yes | • Unclear | • No | • No |
| Avci (2015) | • Unclear | • Yes | • No | • Unclear | • Yes |
| Ayzac (2008) | • Yes | • Yes | • Yes | • Yes | • Yes |
| Bailit (2006) | • Yes | • Yes | • No | • Yes | • Yes |
| Bailit (2013) | • Yes | • Yes | • No | • Yes | • Yes |
| Bakr (2005) | • Yes | • Yes | • No | • Yes | • Yes |
| Balestena (2015) | • No | • Yes | • No | • Yes | • No |
| Bauer (2013) | • Yes | • Yes | • Yes | • Yes | • Yes |
| Bear (2016) | • Yes | • Yes | • Yes | • Yes | • No |
| Belfort (2010) | • Yes | • Yes | • No | • Yes | • No |
| Ben (2007) | • Yes | • Yes | • Yes | • Yes | • No |
| Benincasa (2012) | • Yes | • Yes | • No | • Yes | • No |
| Berg (2009) | • Yes | • Yes | • Yes | • Yes | • No |
| Bianco (2013) | • Unclear | • Yes | • Yes | • Unclear | • Yes |
| Bleich (2012) | • Yes | • Yes | • No | • Yes | • Yes |
| Boccardo (2013) | • Yes | • Yes | • Yes | • Yes | • Yes |
| Bodner (2011) | • Unclear | • Yes | • No | • Unclear | • Yes |
| Borders (2012) | • Unclear | • Yes | • No | • Yes | • Yes |
| Braun (2015) | • Yes | • Yes | • Yes | • Yes | • Yes |
| Butchon (2014) | • Yes | • Yes | • Yes | • Yes | • Yes |
| Callaghan (2008) | • Yes | • Yes | • Yes | • Yes | • Yes |
| Cape (2013) | • Yes | • Yes | • Yes | • Yes | • No |
| Caughey (2007) | • Yes | • Yes | • Yes | • Yes | • Yes |
| Cavazos-Rehg (2015) | • Yes | • Yes | • Unclear | • Yes | • Yes |
| Charrier (2010) | • Yes | • Yes | • Yes | • Yes | • No |
| Cheng (2007) | • Yes | • Yes | • Unclear | • Yes | • Yes |
| Cheng (2010) | • Yes | • Yes | • Yes | • Yes | • Yes |
| Chongsuvivatwong (2010) | • Unclear | • Unclear | • Unclear | • Yes | • No |
| Danilack (2015) | • Unclear | • Yes | • No | • Yes | • No |
| Danish (2010) | • Unclear | • Unclear | • No | • Unclear | • No |
| Darmstadt (2009) | • Yes | • Yes | • Yes | • Yes | • Unclear |
| Dasgupta (2014) | • Unclear | • No | • No | • Unclear | • Yes |
| David (2012) | • Yes | • Yes | • No | • Unclear | • No |
| Debasmita (2010) | • No | • Unclear | • No | • Yes | • No |
| Dimitriu (2010) | • Yes | • Yes | • No | • Unclear | • No |
| dong (2009) | • Yes | • Yes | • No | • Yes | • Yes |
| Dong (2010) | • Unclear | • Yes | • Yes | • Yes | • Yes |
| Dotters-Katz (2015) | • Yes | • Yes | • Yes | • Yes | • Yes |
| Dumas (2008) | • Yes | • Yes | • Yes | • Unclear | • Yes |
| Edwards (2015) | • Yes | • Yes | • Yes | • Yes | • Yes |
| Escosteguy (2013) | • Yes | • Yes | • Yes | • Yes | • Yes |
| Ezugwu (2011) | • Yes | • No | • No | • Unclear | • No |
| Fassett (2013) | • Yes | • Yes | • Yes | • Yes | • No |
| Fronczak (2005) | • Yes | • No | • Yes | • No | • Yes |
| Galyean (2009) | • Yes | • Yes | • No | • Yes | • No |
| Geller (2010) | • Yes | • Yes | • No | • Yes | • Yes |
| Getahun (2010) | • Unclear | • Yes | • Yes | • Yes | • No |
| Gibson (2014) | • Yes | • No | • No | • Yes | • Yes |
| Goff (2013) | • Yes | • Yes | • No | • Yes | • Yes |
| Gozum (2005) | • Unclear | • No | • No | • No | • Yes |
| Grotegut (2008) | • Unclear | • Yes | • Yes | • Yes | • Yes |
| Guendelman (2006) | • Yes | • Yes | • Yes | • Yes | • Yes |
| Guimaraes (2007) | • Yes | • Yes | • Yes | • Unclear | • Yes |
| Harrison (2015) | • Yes | • Yes | • No | • Unclear | • Yes |
| Huda (2012) | • Yes | • Yes | • Yes | • Yes | • No |
| Ivanov (2014) | • Yes | • Yes | • No | • Yes | • No |
| Iyengar (2012) | • Yes | • Yes | • No | • Yes | • Yes |
| Jaleel (2009) | • No | • Unclear | • No | • Unclear | • Yes |
| Janssen (2009) | • Yes | • Yes | • Unclear | • Unclear | • Yes |
| Jin (2011) | • Unclear | • Yes | • No | • Yes | • Yes |
| Jokhio (2005) | • Yes | • Yes | • No | • No | • Yes |
| Karlstrom (2013) | • Yes | • Yes | • No | • Yes | • Yes |
| Karolinski (2013) | • Unclear | • Yes | • Yes | • Yes | • Unclear |
| King (2012) | • Yes | • Yes | • Yes | • Yes | • No |
| Knowles (2014) | • Yes | • Yes | • Yes | • Yes | • Yes |
| Kovavisarach (2005) | • Unclear | • Yes | • Unclear | • Yes | • No |
| Kovavisarach (2010) | • Unclear | • Yes | • No | • Unclear | • Yes |
| Kuklina (2008) | • Yes | • Yes | • Yes | • Yes | • Yes |
| Kyser (2012) | • Yes | • Yes | • Unclear | • Yes | • Yes |
| Latif (2013) | • Yes | • Yes | • No | • Unclear | • Yes |
| Laws (2014) | • Unclear | • Yes | • Unclear | • Unclear | • Yes |
| Leth (2009) | • Yes | • Yes | • No | • Yes | • Yes |
| Liu (2007) | • Yes | • Yes | • Yes | • Yes | • Yes |
| Liu (2010) | • Unclear | • Yes | • Yes | • Yes | • No |
| Lulu (2014) | • Unclear | • Yes | • Yes | • Yes | • Yes |
| Luz (2008) | • Yes | • Yes | • Yes | • Yes | • Yes |
| Lyndon (2012) | • Yes | • Yes | • Unclear | • Yes | • Yes |
| Magann (2008) | • Yes | • Yes | • Unclear | • Yes | • Yes |
| Magann (2011) | • Yes | • Yes | • Unclear | • Yes | • Yes |
| Malloy (2014) | • Yes | • Yes | • Unclear | • Yes | • Yes |
| Maric (2006) | • No | • Yes | • No | • Yes | • No |
| Matsuda (2011) | • Yes | • Unclear | • Yes | • Yes | • Yes |
| Mayi-Tsonga (2007) | • Yes | • Yes | • Yes | • Yes | • Yes |
| Nasreen (2007) | • Yes | • Yes | • No | • No | • Yes |
| Nelson (2014) | • Yes | • Yes | • No | • Yes | • Yes |
| Ngoc (2005) | • Unclear | • Yes | • Unclear | • Yes | • Yes |
| Ngoga (2009) | • No | • Yes | • Unclear | • Unclear | • Yes |
| Okumura (2014) | • Yes | • Yes | • No | • Yes | • Yes |
| Oladapo (2007) | • No | • Yes | • No | • Yes | • Yes |
| Osmundson (2011) | • Yes | • Yes | • No | • Unclear | • Yes |
| Pallasmaa (2008) | • Yes | • Yes | • No | • Yes | • Yes |
| Pallasmaa (2015) | • Yes | • Yes | • No | • Yes | • Yes |
| Palmer (2015) | • Yes | • Yes | • Yes | • Yes | • Yes |
| Panichkul (2007) | • Yes | • Yes | • No | • Yes | • Yes |
| Peret (2007) | • No | • Yes | • No | • Yes | • Yes |
| Ramírez-Villalobos (2009) | • No | • No | • Yes | • Yes | • Yes |
| Saizonou (2014) | • Yes | • Yes | • No | • Yes | • Yes |
| Sanabria (2011) | • Unclear | • Yes | • No | • Unclear | • No |
| Shah (2011) | • Unclear | • Yes | • Unclear | • Yes | • No |
| Shazia (2015) | • No | • Unclear | • No | • No | • No |
| Shriraam (2012) | • Unclear | • Yes | • No | • No | • Yes |
| Simoes (2005) | • Yes | • Yes | • Unclear | • Yes | • No |
| Tabcharoen (2009) | • Yes | • Yes | • No | • Yes | • Yes |
| Wang (2010) | • Yes | • Yes | • Yes | • Yes | • Yes |
| Winani (2007) | • Unclear | • Yes | • Yes | • Yes | • Yes |
| Zhang (2005) | • Yes | • Yes | • Yes | • Unclear | • No |

**
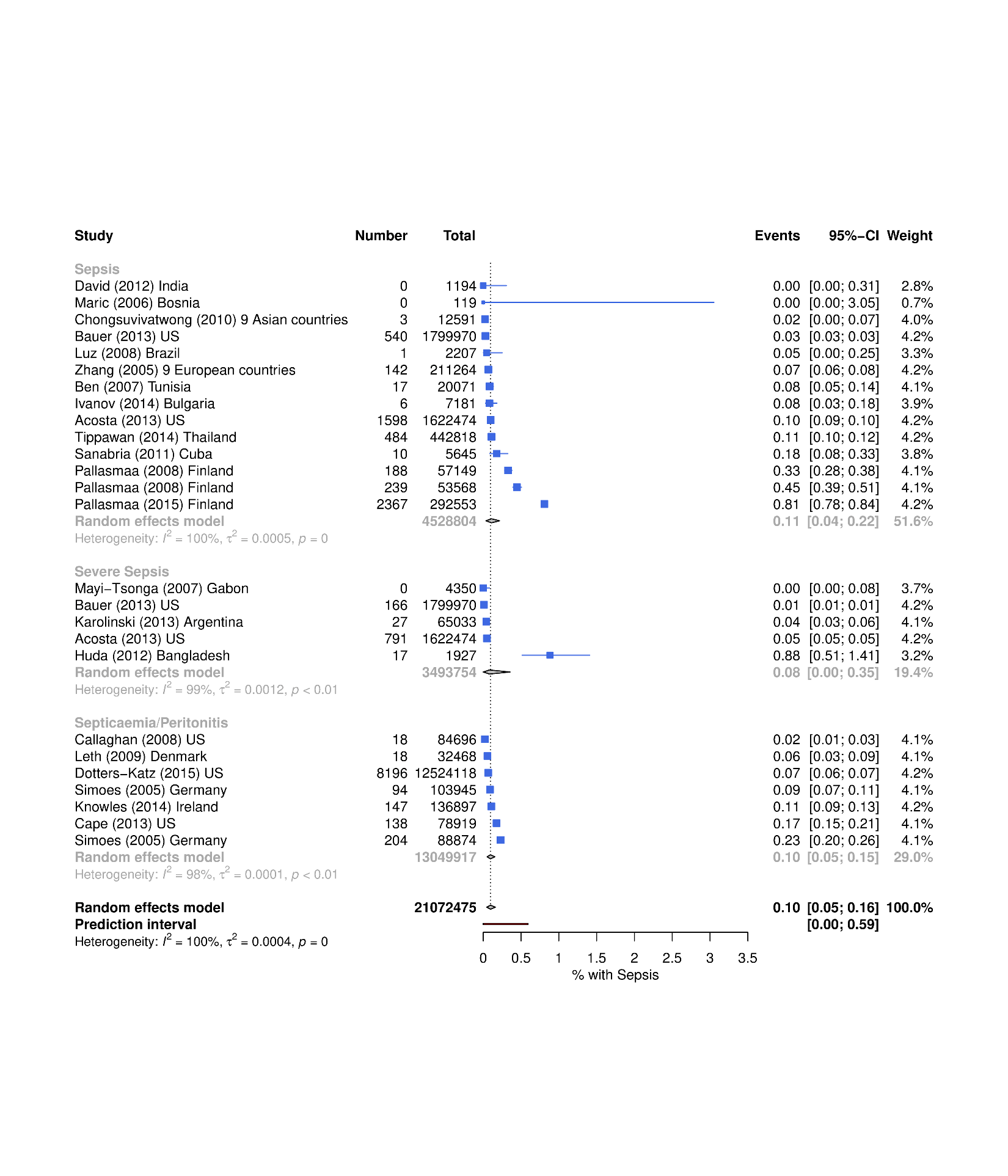
 S6: Forest Plot of sepsis incidence by severity**

# References

1. Abramovici A, Szychowski JM, Biggio JR, Sakawi Y, Andrews WW, Tita AT. Epidural use and clinical chorioamnionitis among women who delivered vaginally. American Journal of Perinatology. 2014;31(11):1009-14

2. Admaty D, Benzing J, Burkhardt T, Lapaire O, Hegi L, Szinnai G, et al. Plasma midregional proadrenomedullin in newborn infants: impact of prematurity and perinatal infection. Pediatric Research. 2012;72(1):70

3. Al‐Ostad G, Kezouh A, Spence AR, Abenhaim HA. Incidence and risk factors of sepsis mortality in labor, delivery and after birth: Population‐based study in the USA. Journal of Obstetrics and Gynaecology Research. 2015;41(8):1201-6

4. Bear JJ, Wu YW. Maternal infections during pregnancy and cerebral palsy in the child. Pediatric Neurology. 2016;57:74-9

5. Berg CJ, MacKay AP, Qin C, Callaghan WM. Overview of maternal morbidity during hospitalization for labor and delivery in the United States: 1993–1997 and 2001–2005. Obstetrics & Gynecology. 2009;113(5):1075-81

6. Bleich AT, Alexander JM, McIntire DD, Leveno KJ. An analysis of second-stage labor beyond 3 hours in nulliparous women. American Journal of Perinatology. 2012;29(09):717-22

7. Borders N, Lawton R, Martin SR. A clinical audit of the number of vaginal examinations in labor: A NOVEL Idea. Journal of Midwifery & Women’s Health. 2012;57(2):139-44

8. Braun D, Bromberger P, Ho NJ, Getahun D. Low rate of perinatal sepsis in term infants of mothers with chorioamnionitis. American Journal of Perinatology. 2016;33(02):143-50

9. Caughey AB, Stotland NE, Washington AE, Escobar GJ. Maternal and obstetric complications of pregnancy are associated with increasing gestational age at term. American Journal of Obstetrics and Gynecology. 2007;196(2):155. e1-. e6

10. Cavazos-Rehg PA, Krauss MJ, Spitznagel EL, Bommarito K, Madden T, Olsen MA, et al. Maternal age and risk of labor and delivery complications. Maternal and Child Health Journal. 2015;19(6):1202-11

11. Cheng YW, Hopkins LM, Laros Jr RK, Caughey AB. Duration of the second stage of labor in multiparous women: maternal and neonatal outcomes. American Journal of Obstetrics and Gynecology. 2007;196(6):585. e1-. e6

12. Cheng YW, Shaffer BL, Bryant AS, Caughey AB. Length of the first stage of labor and associated perinatal outcomes in nulliparous women. Obstetrics & Gynecology. 2010;116(5):1127-35

13. Danilack VA, Nunes AP, Phipps MG. Unexpected complications of low-risk pregnancies in the United States. American Journal of Obstetrics and Gynecology. 2015;212(6):809. e1-. e6

14. Dotters-Katz S, Patel E, Grotegut C, Heine R. Acute infectious morbidity in multiple gestation. Infectious Diseases in Obstetrics and Gynecology. 2015;2015:173261-

15. Edwards SE, Grobman WA, Lappen JR, Winter C, Fox R, Lenguerrand E, et al. Modified obstetric early warning scoring systems (MOEWS): validating the diagnostic performance for severe sepsis in women with chorioamnionitis. American Journal of Obstetrics and Gynecology. 2015;212(4):536. e1-. e8

16. Geller EJ, Wu JM, Jannelli ML, Nguyen TV, Visco AG. Maternal outcomes associated with planned vaginal versus planned primary cesarean delivery. American Journal of Perinatology. 2010;27(09):675-84

17. Getahun D, Strickland D, Zeiger RS, Fassett MJ, Chen W, Rhoads GG, et al. Effect of chorioamnionitis on early childhood asthma. Archives of Pediatrics & Adolescent Medicine. 2010;164(2):187-92

18. Getahun D. Temporal trends in chorioamnionitis by maternal race/ethnicity and gestational age (1995–2010). International Journal of Reproductive Medicine. 2013;2013(Article ID 906467):6 pages

19. Grotegut CA, Tatineni H, Dandolu V, Whiteman VE, Katari S, Geifman-Holtzman O. Obstetric outcomes with a false-positive one-hour glucose challenge test by the Carpenter-Coustan criteria. The Journal of Maternal-Fetal & Neonatal Medicine. 2008;21(5):315-20

20. King JR, Korst LM, Miller DA, Ouzounian JG. Increased composite maternal and neonatal morbidity associated with ultrasonographically suspected fetal macrosomia. The Journal of Maternal-Fetal & Neonatal Medicine. 2012;25(10):1953-9

21. Magann EF, Doherty DA, Briery CM, Niederhauser A, Chauhan SP, Morrison JC. Obstetric characteristics for a prolonged third stage of labor and risk for postpartum hemorrhage. Gynecologic and obstetric investigation. 2008;65(3):201-5

22. Malloy M. Chorioamnionitis: epidemiology of newborn management and outcome United States 2008. Journal of Perinatology. 2014;34(8):611

23. Matsuda Y, Kawamichi Y, Hayashi K, Shiozaki A, Satoh S, Saito S. Impact of maternal age on the incidence of obstetrical complications in Japan. Journal of Obstetrics and Gynaecology Research. 2011;37(10):1409-14

24. Nelson DB, Lucke AM, McIntire DD, Sánchez PJ, Leveno KJ, Chalak LF. Obstetric antecedents to body-cooling treatment of the newborn infant. American Journal of Obstetrics and Gynecology. 2014;211(2):155. e1-. e6

25. Osmundson S, Ou-Yang RJ, Grobman WA. Elective induction compared with expectant management in nulliparous women with an unfavorable cervix. Obstetrics & Gynecology. 2011;117(3):583-7

26. Shah N, Rohra DK, Shuja S, Liaqat NF, Solangi NA, Kumar K, et al. Comparision of obstetric outcome among teenage and non-teenage mothers from three tertiary care hospitals of Sindh, Pakistan. JPMA-Journal of the Pakistan Medical Association. 2011;61(10):963

27. Suthee Panichkul M, Boonprasert K, Komolpis S, Panichkul P. The association between meconium-stained amniotic fluid and chorioamnionitis or endometritis. J Med Assoc Thai. 2007;90(3):442-7

28. The Healthcare Cost and Utilization Project (HCUP). Overview of the National (Nationwide) Inpatient Sample (NIS) 2016. Available from: <https://unstats.un.org/sdgs/indicators/regional-groups/>.

29. Centers for Disease Control and Prevention. National Hospital Discharge Survey. Available from: <https://www.cdc.gov/nchs/nhds/index.htm>.

30. Ahnfeldt-Mollerup P, Petersen LK, Kragstrup J, Christensen RD, Sørensen B. Postpartum infections: occurrence, healthcare contacts and association with breastfeeding. Acta Obstetricia et Gynecologica Scandinavica. 2012;91(12):1440-4

31. Ayzac L, Caillat-Vallet E, Girard R, Chapuis C, Depaix F, Dumas A-M, et al. Decreased rates of nosocomial endometritis and urinary tract infection after vaginal delivery in a French surveillance network, 1997–2003. Infection Control & Hospital Epidemiology. 2008;29(6):487-95

32. Belfort MA, Clark SL, Saade GR, Kleja K, Dildy III GA, Van Veen TR, et al. Hospital readmission after delivery: evidence for an increased incidence of nonurogenital infection in the immediate postpartum period. American Journal of Obstetrics and Gynecology. 2010;202(1):35. e1-. e7

33. Benincasa BC, Walker C, Cioba C, Rosa CCdS, Martins DE, Oliveira EDAd, et al. [Rates of infection related to cesarean and vaginal delivery at HCPA]. Revista HCPA Porto Alegre. 2012 2012;32(1):5-9

34. Bianco A, Roccia S, Nobile CG, Pileggi C, Pavia M. Postdischarge surveillance following delivery: the incidence of infections and associated factors. American Journal of Infection Control. 2013;41(6):549-53

35. Boccardo J, Manzur A, Duarte N, Yanzon C, Mazzanti A, Paparotti L, et al. [Puerperal endometritis in our setting]. Actual SIDA Infectol. 2013;21(80):48-52

36. Chongsuvivatwong V, Bachtiar H, Chowdhury ME, Fernando S, Suwanrath C, Kor‐anantakul O, et al. Maternal and fetal mortality and complications associated with cesarean section deliveries in teaching hospitals in Asia. Journal of Obstetrics and Gynaecology Research. 2010;36(1):45-51

37. Darmstadt GL, Hassan M, Balsara ZP, Winch PJ, Gipson R, Santosham M. Impact of clean delivery-kit use on newborn umbilical cord and maternal puerperal infections in Egypt. Journal of Health, Population, and Nutrition. 2009;27(6):746

38. Dimitriu G. [Clinical statistical study on puerperal sepsis risk factors]. Revista Medico-chirurgicala a Societatii de Medici si Naturalisti din Iasi. 2010;114(1):195-8

39. Dumas A-M, Girard R, Ayzac L, Beaumont G, Caillat-Vallet E, Depaix F, et al. Effect of intrapartum antibiotic prophylaxis against group B streptococcal infection on comparisons of rates of endometritis and urinary tract infection in multicenter surveillance. Infection Control & Hospital Epidemiology. 2008;29(4):327-32

40. Ezugwu E, Onah H, Iyoke C, Ezugwu F. Obstetric outcome following free maternal care at Enugu State University Teaching Hospital (ESUTH), Parklane, Enugu, South-eastern Nigeria. Journal of Obstetrics and Gynaecology. 2011;31(5):409-12

41. Fronczak N, Antelman G, Moran A, Caulfield L, Baqui A. Delivery‐related complications and early postpartum morbidity in Dhaka, Bangladesh. International Journal of Gynecology & Obstetrics. 2005;91(3):271-8

42. Ghani N, Rukanuddin RJ, Ali TS. Prevalence and factors associated with postpartum vaginal infection in the Khyber agency federally administered tribal areas, Pakistan. Journal of Pakistan Medical Association. 2007;57(7):363

43. Gözüm S, Kiliç D. Health problems related to early discharge of Turkish women. Midwifery. 2005;21(4):371-8

44. Guimarães EER, Chianca TCM, Oliveira ACd. [Puerperal infection from the perspective of humanized delivery care at a public maternity hospital]. Revista Latino-Americana de Enfermagem. 2007;15(4):536-42

45. Ivanov S, Tzvetkov K, Kovachev E, Staneva D, Nikolov D. [Puerperal infections after Cesarean section and after a natural childbirth]. Akusherstvo i Ginekologiia. 2014;53:25-8

46. Iyengar K. Early postpartum maternal morbidity among rural women of Rajasthan, India: a community-based study. Journal of Health, Population, and Nutrition. 2012;30(2):213

47. Jokhio AH, Winter HR, Cheng KK. An intervention involving traditional birth attendants and perinatal and maternal mortality in Pakistan. New England Journal of Medicine. 2005;352(20):2091-9

48. Kovavisarach E, Jirasettasiri P. Randomised controlled trial of perineal shaving versus hair cutting in parturients on admission in labor. J Med Assoc Thai. 2005;88(9):1167

49. Magann EF, Doherty DA, Chauhan SP, Klimpel JM, Huff SD, Morrison JC. Pregnancy, obesity, gestational weight gain, and parity as predictors of peripartum complications. Archives of Gynecology and Obstetrics. 2011;284(4):827-36

50. Marić T, Tomić V, Darko K. [Puerperal complications in nulliparous women delivered by section caesarean: pair study]. Medicinski Arhiv. 2006;60(4):246-50

51. Ngoga E, Hall D, Mattheyse F, Grové D. Outcome of pregnancy in the morbidly obese woman. South African Family Practice. 2009;51(1)

52. Sánchez JMB, Serrano YP, Soler JRM. [Advanced maternal age as a conducive element in obstetric complications and birth]. Revista de Ciencias Médicas de Pinar del Río. 2015;19(5):789-802

53. Péret FJA, Melo VH, Paula LBd, Andrade BAMd, Pinto JA. [Puerperal morbidity in HIV-infected and non-infected women]. Revista Brasileira de Ginecologia e Obstetricia. 2007;29(5):260-6

54. Ramírez-Villalobos D, Hernández-Garduño A, Salinas A, González D, Walker D, Rojo-Herrera G, et al. [Early postpartum discharge and complications in the early puerperium]. Salud Pública de México. 2009;51(3):212-8

55. Saizonou J, Ouédraogo L, Paraiso MN, Ayélo P, Kpozèhouen A, Daraté R, et al. [Epidemiology and management of intrapartum infections in the maternity ward of Ouémé-Plateau county hospital in Benin]. The Pan African Medical Journal. 2014;17:89-

56. Sanabria Fromherz ZE, Fernández Arenas C. [Pathologic behavior of puerperium] Revista Cubana de Obstetrícia y Ginecologia. 2011;37(3):330-40

57. Tabcharoen C, Pinjaroen S, Suwanrath C, Krisanapan O. Pregnancy outcome after age 40 and risk of low birth weight. Journal of Obstetrics and Gynaecology. 2009;29(5):378-83

58. Winani S, Wood S, Coffey P, Chirwa T, Mosha F, Changalucha J. Use of a clean delivery kit and factors associated with cord infection and puerperal sepsis in Mwanza, Tanzania. Journal of Midwifery & Women's Health. 2007;52(1):37-43

59. Awan S, Bibi S, Makhdoom A, Farooq S, SM T, Qazi RA. Adverse fetomaternal outcome among pregnant overweight women. Pakistan Journal of Medical Sciences. 2015;31(2):383

60. Bailit JL, Love TE, Dawson NV. Quality of obstetric care and risk-adjusted primary cesarean delivery rates. American Journal of Obstetrics and Gynecology. 2006;194(2):402-7

61. Bodner K, Wierrani F, Grünberger W, Bodner-Adler B. Influence of the mode of delivery on maternal and neonatal outcomes: a comparison between elective cesarean section and planned vaginal delivery in a low-risk obstetric population. Archives of Gynecology and Obstetrics. 2011;283(6):1193-8

62. Charrier L, Serafini P, Chiono V, Rebora M, Rabacchi G, Zotti CM. Clean and sterile delivery: two different approaches to infection control. Journal of Evaluation in Clinical Practice. 2010;16(4):771-5

63. Danish N, Fawad A, Abbasi N. Assessment of pregnancy outcome in primigravida: comparison between booked and un-booked patients. Journal of Ayub Medical College Abbottabad. 2010;22(2):23-5

64. Dasgupta A, Harichandrakumar K, Habeebullah S. Pregnancy outcome among obese Indians-a prospective cohort study in a tertiary Care Centre in South India. International Journal of Scientific Study. 2014;2(2):13-8

65. Dong L. [Management and Monitoring Measure of Nosocomial Infection in Cesarean Section, Normal Delivery, Domiciliary Delivery Integral Delivery Room]. Chinese Journal of Nosocomiology. 2009 (16):39

66. Dong L, Wang G. [Effect of hand washing method to maternity and infant]. Chinese Journal of Nosocomiology. 2010;20(15):2257-9

67. Goff SL, Pekow PS, Avrunin J, Lagu T, Markenson G, Lindenauer PK. Patterns of obstetric infection rates in a large sample of US hospitals. American Journal of Obstetrics and Gynecology. 2013;208(6):456. e1-. e13

68. Jaleel R. Impact of maternal obesity on pregnancy outcome. Journal of Surgery Pakistan (International). 2009;14(1)

69. Janssen PA, Saxell L, Page LA, Klein MC, Liston RM, Lee SK. Outcomes of planned home birth with registered midwife versus planned hospital birth with midwife or physician. CMAJ. 2009;181(6-7):377-83

70. Latif T, Ali M, Majeed A, Nahar K, Noor Z. Labor outcome of primigravidae in Mymensingh Medical College Hospital. Mymensingh Medical Journal. 2013;22(3):432-7

71. Leth RA, Møller JK, Thomsen RW, Uldbjerg N, Nørgaard M. Risk of selected postpartum infections after cesarean section compared with vaginal birth: A five‐year cohort study of 32,468 women. Acta Obstetricia et Gynecologica Scandinavica. 2009;88(9):976-83

72. Liu H, Cui Y. [Relationship between body mass index of pregnant women and postpartum incision infection]. Maternal and Child Health Care of China. 2010;25(18):2485-6

73. Oladapo OT, Lamina MA, SULE‐ODU AO. Maternal morbidity and mortality associated with elective caesarean delivery at a university hospital in Nigeria. Australian and New Zealand journal of obstetrics and gynaecology. 2007;47(2):110-4

74. Petter CE, Farret TCF, de Souza Scherer J, Antonello VS. [Factors related to surgical site infections after obstetric procedures]. Scientia Medica. 2013;23(1):5

75. Shriraam V, Shah P, Rani M, Palani G, Sathiyasekaran B. Postpartum morbidity and health seeking pattern in a rural community in South India–population based study. Indian Journal of Maternal and Child Health. 2012;14(3):10

76. Acosta CD, Knight M, Lee HC, Kurinczuk JJ, Gould JB, Lyndon A. The continuum of maternal sepsis severity: incidence and risk factors in a population-based cohort study. PLOS One. 2013;8(7):e67175

77. Bauer ME, Bateman BT, Bauer ST, Shanks AM, Mhyre JM. Maternal sepsis mortality and morbidity during hospitalization for delivery: temporal trends and independent associations for severe sepsis. Anesthesia & Analgesia. 2013;117(4):944-50

78. Ben SH, Khoudayer H, Ben HZ, Masmoudi A, Bouguerra B, Sfar R. [Severe maternal morbidity]. Journal de Gynecologie, Obstetrique et Biologie de la Reproduction. 2007;36(7):694-8

79. Callaghan WM, MacKay AP, Berg CJ. Identification of severe maternal morbidity during delivery hospitalizations, United States, 1991-2003. American Journal of Obstetrics and Gynecology. 2008;199(2):133. e1-. e8

80. Cape A, Tuomala RE, Taylor C, Puopolo KM. Peripartum bacteremia in the era of group B streptococcus prophylaxis. Obstetrics & Gynecology. 2013;121(4):812-8

81. David K, Pricilla R, Venkatesan S, Rahman S, Sy G, Vijayaselvi R. Outcomes of deliveries in a midwife-run labour room located at an urban health centre: results of a 5-year retrospective study. The Natl Med J India. 2012;25:323-6

82. Huda FA, Ahmed A, Dasgupta SK, Jahan M, Ferdous J, Koblinsky M, et al. Profile of maternal and foetal complications during labour and delivery among women giving birth in hospitals in Matlab and Chandpur, Bangladesh. Journal of Health, Population, and Nutrition. 2012;30(2):131

83. Karolinski A, Mercer R, Micone P, Ocampo C, Mazzoni A, Fontana O, et al. The epidemiology of life‐threatening complications associated with reproductive process in public hospitals in Argentina. BJOG: An International Journal of Obstetrics & Gynaecology. 2013;120(13):1685-95

84. Knowles S, O'sullivan N, Meenan A, Hanniffy R, Robson M. Maternal sepsis incidence, aetiology and outcome for mother and fetus: a prospective study. BJOG: An International Journal of Obstetrics & Gynaecology. 2015;122(5):663-71

85. Kuklina EV, Whiteman MK, Hillis SD, Jamieson DJ, Meikle SF, Posner SF, et al. An enhanced method for identifying obstetric deliveries: implications for estimating maternal morbidity. Maternal and Child Health Journal. 2008;12(4):469-77

86. Luz AG, Tiago DB, Silva JCGd, Amaral E. [Severe maternal morbidity at a local reference university hospital in Campinas, São Paulo, Brazil]. Revista Brasileira de Ginecologia e Obstetrícia. 2008;30(6):281-6

87. Lyndon A, Lee HC, Gilbert WM, Gould JB, Lee KA. Maternal morbidity during childbirth hospitalization in California. The Journal of Maternal-Fetal & Neonatal Medicine. 2012;25(12):2529-35

88. Mayi-Tsonga S, Meyé J-F, Tagne A, Ndombi I, Diallo T, Oksana L, et al. [Audit of the severe obstetrical morbidity (near miss) in Gabon]. Cahiers d'Etudes et de Recherches Francophones/Santé. 2007;17(2):111-5

89. Pallasmaa N, Ekblad U, Gissler M. Severe maternal morbidity and the mode of delivery. Acta obstetricia et gynecologica Scandinavica. 2008;87(6):662-8

90. Pallasmaa N, Ekblad U, Gissler M, Alanen A. The impact of maternal obesity, age, pre-eclampsia and insulin dependent diabetes on severe maternal morbidity by mode of delivery—a register-based cohort study. Archives of Gynecology and Obstetrics. 2015;291(2):311-8

91. Simoes E, Kunz S, Bosing-Schwenkglenks M, Schmahl F. Association between method of delivery and puerperal infectious complications in the perinatal database of Baden-Württemberg 1998–2001. Gynecologic and Obstetric Investigation. 2005;60(4):213-7

92. Tippawan Liabsuetrakul M, Suchonwanich Y. Birth rates and pregnancy complications in adolescent pregnant women giving birth in the hospitals of Thailand. J Med Assoc Thai. 2014;97(8):785-90

93. Zhang WH, Alexander S, Bouvier‐Colle MH, Macfarlane A, Group MB. Incidence of severe pre‐eclampsia, postpartum haemorrhage and sepsis as a surrogate marker for severe maternal morbidity in a European population‐based study: the MOMS‐B survey. BJOG: An International Journal of Obstetrics & Gynaecology. 2005;112(1):89-96

94. Andersson N, Omer K, Caldwell D, Dambam MM, Maikudi AY, Effiong B, et al. Male responsibility and maternal morbidity: a cross-sectional study in two Nigerian states. BMC Health Services Research. 2011;11(2):S7

95. Avcı ME, Şanlıkan F, Celik M, Avcı A, Kocaer M, Göçmen A. Effects of maternal obesity on antenatal, perinatal and neonatal outcomes. The Journal of Maternal-Fetal & Neonatal Medicine. 2015;28(17):2080-3

96. Bailit JL, Grobman WA, Rice MM, Spong CY, Wapner RJ, Varner MW, et al. Risk-adjusted models for adverse obstetric outcomes and variation in risk-adjusted outcomes across hospitals. American Journal of Obstetrics and Gynecology. 2013;209(5):446. e1-. e30

97. Bakr AF, Karkour T. Effect of predelivery vaginal antisepsis on maternal and neonatal morbidity and mortality in Egypt. Journal of Women's Health. 2005;14(6):496-501

98. Chen L, Liu J, Kang Y, Liu J, Sufeng H. [Relationship between pre-pregnant body mass index and pregnancy growth with maternal and neonatal outcomes]. Chongqing Medicine. 2014 (10):1178-80

99. Galyean A, Lagrew D, Bush M, Kurtzman J. Previous cesarean section and the risk of postpartum maternal complications and adverse neonatal outcomes in future pregnancies. Journal of Perinatology. 2009;29(11):726

100. Gibson KS, Waters TP, Bailit JL. Maternal and neonatal outcomes in electively induced low-risk term pregnancies. American Journal of Obstetrics and Gynecology. 2014;211(3):249. e1-. e16

101. Guendelman S, Thornton D, Gould J, Hosang N. Obstetric complications during labor and delivery: assessing ethnic differences in California. Women's Health Issues. 2006;16(4):189-97

102. Harrison MS, Ali S, Pasha O, Saleem S, Althabe F, Berrueta M, et al. A prospective population-based study of maternal, fetal, and neonatal outcomes in the setting of prolonged labor, obstructed labor and failure to progress in low-and middle-income countries. Reproductive Health. 2015;12(2):S9

103. Jin Z, Chi X, Teng W, Wang X, Xu Q, Wang P, et al. [Sex hormone-binding globulin of gestational diabetes mellitus pregnant women with well-controlled glucose and pregnancy outcomes]. Zhonghua fu Chan ke za zhi. 2011;46(6):422-6

104. Karlström A, Lindgren H, Hildingsson I. Maternal and infant outcome after caesarean section without recorded medical indication: findings from a Swedish case–control study. BJOG: An International Journal of Obstetrics & Gynaecology. 2013;120(4):479-86

105. Kovavisarach E, Chairaj S, Tosang K, Asavapiriyanont S, Chotigeat U. Outcome of teenage pregnancy in Rajavithi Hospital. J Med Assoc Thai. 2010;93(1):1

106. Kyser KL, Lu X, Santillan DA, Santillan MK, Hunter SK, Cahill AG, et al. The association between hospital obstetrical volume and maternal postpartum complications. American Journal of Obstetrics and Gynecology. 2012;207(1):42. e1-. e17

107. Laws PJ, Xu F, Welsh A, Tracy SK, Sullivan EA. Maternal morbidity of women receiving birth center care in New South Wales: A matched‐pair analysis using linked health data. Birth. 2014;41(3):268-75

108. Liu S, Liston RM, Joseph K, Heaman M, Sauve R, Kramer MS. Maternal mortality and severe morbidity associated with low-risk planned cesarean delivery versus planned vaginal delivery at term. CMAJ. 2007;176(4):455-60

109. Mandal D, Manda S, Rakshi A, Dey R, Biswas S, Banerjee A. Maternal obesity and pregnancy outcome: a prospective analysis. The Journal of the Association of Physicians of India. 2011;59:486-9

110. Ngoc NT, Sloan NL, Thach TS, Liem LK, Winikoff B. Incidence of postpartum infection after vaginal delivery in Viet Nam. Journal of Health, Population and Nutrition. 2005:121-30

111. Okumura JA, Maticorena DA, Tejeda JE, Mayta-Tristán P. [Teenage pregnancy as a risk factor for obstetric and perinatal complications at a hospital in Lima, Peru]. Revista Brasileira de Saúde Materno Infantil. 2014;14(4):383-92

112. Palmer WL, Bottle A, Aylin P. Association between day of delivery and obstetric outcomes: observational study. BMJ. 2015;351:h5774

113. Wang X, Yu X, Qian X. [Postpartum intrauterine infection: clinical features and preventive measures]. Chinese Journal of Nosocomiology. 2010;20(14):2050-1
