# Supplementary material for: Incidence of maternal peripartum infection: A systematic review and meta-analysis
Source: PLoS Med. 2019 Dec 10;16(12):e1002984. doi: 10.1371/journal.pmed.1002984 (PMC6903710; doi:10.1371/journal.pmed.1002984)
Supplement: S1 Table — (DOCX) [file pmed.1002984.s004.docx]

# S1 Table: ICD Codes for infection outcomes

| **Outcome** | | **ICD-9** | **ICD-10** |
| --- | --- | --- | --- |
| Chorioamnionitis | | 658.4, 659.2, 762.7 | O41.12 |
| Endometritis | | 670.1 | O86.12 |
| Wound infection | | 674.3 – But no studies specified ICD codes | |
| Sepsis | SIRS* (including puerperal sepsis) | 670.2, 995.91 | O85 |
|  | Severe Sepsis | 995.92, 785.52 | R65.20, R65.21 |
|  | Bacteraemia/Septicaemia | 038, 659.3, 790.7 | R78.81, A40, A41 |
| Peripartum infection | | 670 | O86 |
|  |  | Plus a combination of the codes above | |

*Systemic inflammatory response syndrome
