# Supplementary material for: Incidence of maternal peripartum infection: A systematic review and meta-analysis
Source: PLoS Med. 2019 Dec 10;16(12):e1002984. doi: 10.1371/journal.pmed.1002984 (PMC6903710; doi:10.1371/journal.pmed.1002984)
Supplement: S2 Table — (DOCX) [file pmed.1002984.s005.docx]

# S2 Table: Studies of Chorioamnionitis

| **Author** | **Date** | **Country** | **Description** | **Total women** | **Chorioamnionitis**  **(%)** | **Quality** | **Not in meta-analysis** |
| --- | --- | --- | --- | --- | --- | --- | --- |
| Abramovici (2014)[1] | 11/08-06/10 | US | Chorioamnionitis extracted from medical records of women in a single-hospital RCT of different oxytocin doses. Low-risk women with vaginal delivery and livebirth at one hospital | 1785 | 6.78 | 4 |  |
| Admaty (2012)[2] | 03/09-12/10 | Switzerland | Signs of chorioamnionitis extracted from maternal medical records for a study of newborn outcomes at different gestational ages in 2 hospital. Term births only | 143 | 0.70 | 2 |  |
| Al-Ostad (2015)[3] | 01/98-12/08 | US | Study of risk factors for sepsis using National Inpatient Sample (NIS) data representing all hospital deliveries in the US | 5338995 | 1.73 | 5 |  |
| Bear (2016)[4] | 01/91-12/01 | US | Medical record discharge diagnosis at all non-federal hospitals in California for a study of cerebral palsy and maternal infection | 6018504 | 1.84 | 4 |  |
| Berg (2009)[5] | 01/01-12/05 | US | Study of maternal morbidity during hospitalisation for labour using the National Hospital Discharge Survey representing all hospital deliveries in the US. | 19986000* | 1.50 | 4 | Overlapping data |
| Berg (2009)[5] | 01/93-12/97 | US | As above | 19081000* | 1.90 | 4 |  |
| Bleich (2012)[6] | 01/03-12/08 | US | Medical record data on chorioamnionitis from a study of duration of second stage of labour. Women with live births at 1 hospital | 21991 | 19.66 | 4 | Outlier |
| Borders (2012)[7] | 2009 | US | Audit of number of vaginal examinations in labour and routine midwife diagnosis of chorioamnionitis. Term deliveries at one hospital | 205 | 6.34 | 3 |  |
| Braun (2016)[8] | 01/10-12/10 | US | Study of perinatal sepsis in term infants at 13 hospitals in the Kaiser Permanent Medical Program (KPMP), California, and integrated managed care consortium. Medical record data on chorioamnionitis. | 31112 | 4.00 | 5 |  |
| Caughey (2007)[9] | 01/95-12/99 | US | Study of maternal complications at 13 KPMP facilities. Medical record data of low-risk, term deliveries | 119254 | 3.49 | 5 |  |
| Cavazos-Rehg (2015)[10] | 01/09-12/09 | US | Study of maternal age and delivery complications using NIS data | 4109295 | 1.67 | 5 | Overlapping data |
| Cheng (2007)[11] | 01/91-12/02 | US | Medical record data on chorioamnionitis from a study of maternal and newborn outcomes by duration of second stage of labour. Multiparous women with livebirths at term in one hospital. | 5158 | 4.28 | 4 |  |
| Cheng (2010)[12] | 01/90-07/08 | US | Signs of chorioamnionitis extracted from medical records from a study of perinatal outcomes by duration of first stage of labour. Nulliparous women with live, term births at 1 hospital. | 10661 | 12.56 | 5 |  |
| Danilack (2015)[13] | 01/11-12/13 | US | Chorioamnionitis on birth certificates of all low-risk women delivering in the US | 10458616 | 1.29 | 2 |  |
| Dotters-Katz (2015)[14] | 01/08-12/10 | US | Study of infection in multiple versus single gestation using NIS data | 12524118* | 2.58 | 5 |  |
| Edwards (2015)[15] | 06/06-11/07 | US | Signs of chorioamnionitis extracted from maternal medical records for a study of an early warning system for severe sepsis at one hospital. | 15027 | 6.08 | 5 |  |
| Geller (2010)[16] | 1995-2005 | US | Intrapartum fever extracted from medical records for study of maternal outcomes and planned mode of birth at one hospital. Low-risk, nulliparous women delivering at term | 4048 | 15.74 | 4 |  |
| Getahun (2010)[17] | 01/91-12/07 | US | Medical record data for study of effect of chorioamnionitis on childhood asthma at KPMP hospitals. Only includes infants who became health plan members. | 397852 | 3.20 | 3 | Overlapping data |
| Getahun (2013)[18] | 01/95-12/10 | US | Medical record data of temporal trends in chorioamnionitis in KPMP hospitals. | 471821 | 4.12 | 4 |  |
| Grotegut (2008)[19] | 01/03-06/05 | US | Medical record data on obstetric outcomes with false-positive glucose challenge test (GCT) at 1 hospital. Normal GCT only | 165 | 0.61 | 4 |  |
| King (2012)[20] | 08/95-02/04 | US | Maternal and Neonatal morbidity using the perinatal database at 1 hospital. Live births at term. | 14406 | 12.85 | 4 |  |
| Magann (2008)[21] | 03/04-02/05 | US | Obstetric characteristics for prolonged third stage of labour. Source of data unclear. Vaginal deliveries at a naval medical centre. | 1607 | 2.18 | 4 |  |
| Malloy (2014)[22] | 01/08-12/08 | US | Birth certificate data for study of chorioamnionitis and newborn outcomes. Live, term births across the US | 2224406 | 0.99 | 4 | Overlapping data |
| Matsuda (2011)[23] | 2001-2005 | Japan | Data from perinatal registry network of 125 centres. | 242715 | 1.03 | 4 |  |
| Nelson (2014)[24] | 01/05-12/11 | US | Study of obstetric risk factors for newborn complications. Source of data unclear. Live, term births at 1 hospital | 86371 | 6.61 | 4 |  |
| Osmundson (2011)[25] | 07/06-06/08 | US | Medical record data on chorioamnionitis for a sample of low-risk women managed expectantly (not induced) at 39 weeks gestation in 1 hospital | 102 | 19.61 | 3 |  |
| Shah (2011)[26] | 09/08-11/08 | Pakistan | Medical record data on obstetric outcomes of low-risk women at 3 hospitals. Convenience sample of women aged 20-35 | 916 | 0.76 | 2 |  |
| Suthee (2007)[27] | 01/99-12/03 | Thailand | Signs of chorioamnionitis extracted from medical records in study of meconium-stained amniotic fluid and maternal infection. Low-risk women with live, term birth at 1 hospital | 1079 | 0.93 | 5 |  |

*Results presented are weighted percentage of US population. In meta-analysis we approximated the sample size at 20% for the NIS[28] and 1% for the NHDS.[29]

# References

1. Abramovici A, Szychowski JM, Biggio JR, Sakawi Y, Andrews WW, Tita AT. Epidural use and clinical chorioamnionitis among women who delivered vaginally. American Journal of Perinatology. 2014;31(11):1009-14.

2. Admaty D, Benzing J, Burkhardt T, Lapaire O, Hegi L, Szinnai G, et al. Plasma midregional proadrenomedullin in newborn infants: impact of prematurity and perinatal infection. Pediatric Research. 2012;72(1):70.

3. Al‐Ostad G, Kezouh A, Spence AR, Abenhaim HA. Incidence and risk factors of sepsis mortality in labor, delivery and after birth: Population‐based study in the USA. Journal of Obstetrics and Gynaecology Research. 2015;41(8):1201-6.

4. Bear JJ, Wu YW. Maternal infections during pregnancy and cerebral palsy in the child. Pediatric Neurology. 2016;57:74-9.

5. Berg CJ, MacKay AP, Qin C, Callaghan WM. Overview of maternal morbidity during hospitalization for labor and delivery in the United States: 1993–1997 and 2001–2005. Obstetrics & Gynecology. 2009;113(5):1075-81.

6. Bleich AT, Alexander JM, McIntire DD, Leveno KJ. An analysis of second-stage labor beyond 3 hours in nulliparous women. American Journal of Perinatology. 2012;29(09):717-22.

7. Borders N, Lawton R, Martin SR. A clinical audit of the number of vaginal examinations in labor: A NOVEL Idea. Journal of Midwifery & Women’s Health. 2012;57(2):139-44.

8. Braun D, Bromberger P, Ho NJ, Getahun D. Low rate of perinatal sepsis in term infants of mothers with chorioamnionitis. American Journal of Perinatology. 2016;33(02):143-50.

9. Caughey AB, Stotland NE, Washington AE, Escobar GJ. Maternal and obstetric complications of pregnancy are associated with increasing gestational age at term. American Journal of Obstetrics and Gynecology. 2007;196(2):155. e1-. e6.

10. Cavazos-Rehg PA, Krauss MJ, Spitznagel EL, Bommarito K, Madden T, Olsen MA, et al. Maternal age and risk of labor and delivery complications. Maternal and Child Health Journal. 2015;19(6):1202-11.

11. Cheng YW, Hopkins LM, Laros Jr RK, Caughey AB. Duration of the second stage of labor in multiparous women: maternal and neonatal outcomes. American Journal of Obstetrics and Gynecology. 2007;196(6):585. e1-. e6.

12. Cheng YW, Shaffer BL, Bryant AS, Caughey AB. Length of the first stage of labor and associated perinatal outcomes in nulliparous women. Obstetrics & Gynecology. 2010;116(5):1127-35.

13. Danilack VA, Nunes AP, Phipps MG. Unexpected complications of low-risk pregnancies in the United States. American Journal of Obstetrics and Gynecology. 2015;212(6):809. e1-. e6.

14. Dotters-Katz S, Patel E, Grotegut C, Heine R. Acute infectious morbidity in multiple gestation. Infectious Diseases in Obstetrics and Gynecology. 2015;2015:173261-.

15. Edwards SE, Grobman WA, Lappen JR, Winter C, Fox R, Lenguerrand E, et al. Modified obstetric early warning scoring systems (MOEWS): validating the diagnostic performance for severe sepsis in women with chorioamnionitis. American Journal of Obstetrics and Gynecology. 2015;212(4):536. e1-. e8.

16. Geller EJ, Wu JM, Jannelli ML, Nguyen TV, Visco AG. Maternal outcomes associated with planned vaginal versus planned primary cesarean delivery. American Journal of Perinatology. 2010;27(09):675-84.

17. Getahun D, Strickland D, Zeiger RS, Fassett MJ, Chen W, Rhoads GG, et al. Effect of chorioamnionitis on early childhood asthma. Archives of Pediatrics & Adolescent Medicine. 2010;164(2):187-92.

18. Getahun D. Temporal trends in chorioamnionitis by maternal race/ethnicity and gestational age (1995–2010). International Journal of Reproductive Medicine. 2013;2013(Article ID 906467):6 pages. doi: <https://doi.org/10.1155/2013/906467>.

19. Grotegut CA, Tatineni H, Dandolu V, Whiteman VE, Katari S, Geifman-Holtzman O. Obstetric outcomes with a false-positive one-hour glucose challenge test by the Carpenter-Coustan criteria. The Journal of Maternal-Fetal & Neonatal Medicine. 2008;21(5):315-20.

20. King JR, Korst LM, Miller DA, Ouzounian JG. Increased composite maternal and neonatal morbidity associated with ultrasonographically suspected fetal macrosomia. The Journal of Maternal-Fetal & Neonatal Medicine. 2012;25(10):1953-9.

21. Magann EF, Doherty DA, Briery CM, Niederhauser A, Chauhan SP, Morrison JC. Obstetric characteristics for a prolonged third stage of labor and risk for postpartum hemorrhage. Gynecologic and obstetric investigation. 2008;65(3):201-5.

22. Malloy M. Chorioamnionitis: epidemiology of newborn management and outcome United States 2008. Journal of Perinatology. 2014;34(8):611.

23. Matsuda Y, Kawamichi Y, Hayashi K, Shiozaki A, Satoh S, Saito S. Impact of maternal age on the incidence of obstetrical complications in Japan. Journal of Obstetrics and Gynaecology Research. 2011;37(10):1409-14.

24. Nelson DB, Lucke AM, McIntire DD, Sánchez PJ, Leveno KJ, Chalak LF. Obstetric antecedents to body-cooling treatment of the newborn infant. American Journal of Obstetrics and Gynecology. 2014;211(2):155. e1-. e6.

25. Osmundson S, Ou-Yang RJ, Grobman WA. Elective induction compared with expectant management in nulliparous women with an unfavorable cervix. Obstetrics & Gynecology. 2011;117(3):583-7.

26. Shah N, Rohra DK, Shuja S, Liaqat NF, Solangi NA, Kumar K, et al. Comparision of obstetric outcome among teenage and non-teenage mothers from three tertiary care hospitals of Sindh, Pakistan. JPMA-Journal of the Pakistan Medical Association. 2011;61(10):963.

27. Suthee Panichkul M, Boonprasert K, Komolpis S, Panichkul P. The association between meconium-stained amniotic fluid and chorioamnionitis or endometritis. J Med Assoc Thai. 2007;90(3):442-7.

28. The Healthcare Cost and Utilization Project (HCUP). Overview of the National (Nationwide) Inpatient Sample (NIS) 2016. Available from: <https://unstats.un.org/sdgs/indicators/regional-groups/>.

29. Centers for Disease Control and Prevention. National Hospital Discharge Survey. Available from: <https://www.cdc.gov/nchs/nhds/index.htm>.
