# Supplementary material for: Incidence of maternal peripartum infection: A systematic review and meta-analysis
Source: PLoS Med. 2019 Dec 10;16(12):e1002984. doi: 10.1371/journal.pmed.1002984 (PMC6903710; doi:10.1371/journal.pmed.1002984)
Supplement: S3 Table — (DOCX) [file pmed.1002984.s006.docx]

**S3 Table: Studies of Endometritis**

| **Author** | **Date** | **Country** | **Description** | **Total women** | **Endometritis (%)** | **Quality** | **Not in meta-analysis** |
| --- | --- | --- | --- | --- | --- | --- | --- |
| Ahnfeldt-Mollerup (2012)[1] | 05/07-04/08 | Denmark | Questionnaire sent to women 28 days after delivering at 1 regional hospital. Report of infection validated with data from General Practice and hospital records. | 1616 | 1.86 | 2 |  |
| Ayzac (2008)[2] | 01/97-12/03 | France | Clinical endometritis after vaginal delivery until 30 days postpartum at 66 hospitals in a surveillance network. | 161077 | 0.33 | 5 |  |
| Belfort (2010)[3] | 01/07-12/07 | US | Women readmitted with clinical uterine infection up to 42 days postpartum. Medical record data from 114 hospitals representative of the US population. | 222751 | 0.15 | 4 |  |
| Benincasa (2012)[4] | 01/04-12/10 | Brazil | Medical record data on clinical puerperal infection at 1 hospital | 26691 | 1.47 | 3 |  |
| Bianco (2013)[5] | 09/07-09/08 | Italy | Telephone calls with women at 30 days after delivery at 1 hospital. Postpartum infections corroborated by hospital and physician visits, wound cultures and antibiotic prescriptions. | 1656 | 1.39 | 3 |  |
| Boccardo (2013)[6] | 04/10-07/10 | Argentina | Medical record data on clinical endometritis in 1 public hospital. | 1472 | 2.51 | 5 |  |
| Caughey (2007)[7] | 01/95-12/99 | US | Maternal complications by gestational age. Medical record data on endometritis at 13 Californian hospitals in an insurance programme (KPMP) | 119254 | 1.20 | 5 |  |
| Cavazos-Rehg (2015)[8] | 01/09-12/09 | US | Maternal age and delivery complications using NIS data | 4109295 | 0.36 | 4 | Overlapping data |
| Cheng (2007)[9] | 01/91-12/02 | US | Maternal and newborn outcomes by duration of 2nd stage of labour in multiparous women. Medical record data at 1 hospital | 5158 | 1.36 | 4 |  |
| Cheng (2010)[10] | 01/90-07/08 | US | Perinatal outcomes by duration of 1st-stage of labour in nulliparous women. Medical record data at 1 hospital | 10661 | 2.37 | 5 |  |
| Chongsuvivatwong (2010)[11] | 09/01-09/04 | 9 Asian countries | Clinical data on maternal and foetal complications collected by checklist until day 5 postpartum in 12 teaching hospitals in Asia. Vaginal deliveries only. | 12591 | 0.06 | 2 |  |
| Darmstadt (2009)[12] | 06/01-07/01 | Egypt | Study of clean delivery-kit use in 1 urban and 2 rural areas. Infection diagnosed by nurse at week 1 postnatal home visit | 334 | 1.50 | 4 |  |
| Dimitriu (2010)[13] | 1/1/06-1/9/09 | Kuwait | Medical record data of puerperal infection at 1 hospital | 7550 | 1.63 | 2 |  |
| Dotters-Katz (2015)[14] | 01/08-12/10 | US | Endometritis in single and multiple gestation using NIS data | 12524118* | 1.36 | 5 |  |
| Dumas (2008)[15] | 01/01-12/04 | France | Clinical endometritis after vaginal delivery until 30 days postpartum at 44 hospitals in a surveillance network. | 49786 | 0.23 | 4 | Overlapping data |
| Ezugwu (2011)[16] | 09/08-12/08 | Nigeria | Medical record data on obstetric outcomes, including genital sepsis, at 1 hospital during the period of free maternal care. | 1152 | 1.74 | 1 |  |
| Fronczak (2005)[17] | 11/93-05/95 | Bangladesh | Multi-stage probability sampling of women in slum areas of Dhaka. Pelvic infection identified at interviews conducted at home at 72 hours, 7 days and, with examination by a doctor, 14-22 days postpartum. | 1506 | 14.01 | 3 | Outlier |
| Geller (2010)[18] | 1995 -2005 | US | Medical record data on maternal outcomes and planned mode of birth among nulliparous, low-risk women at 1 hospital. | 4048 | 1.31 | 4 |  |
| Ghani (2007)[19] | 1/7/05-31/7/05 | Pakistan | Self-reported symptoms of vaginal infection during interview at home by trained nurse/midwife. Simple random sample of postpartum women in the Khyber Agency | 1000 | 16.20 | 3 | Outlier |
| Gozum (2005)[20] | 05/00-06/00 | Turkey | Vaginal infection until 6 weeks postpartum, reported during interviews with mothers attending for 2 month infant immunisations at 1 primary care unit | 112 | 14.29 | 1 | Outlier |
| Grotegut (2008)[21] | 01/03-06/05 | US | Medical record data on obstetric outcomes with false-positive glucose challenge test (GCT) at 1 hospital. Normal GCT only | 165 | 1.21 | 4 |  |
| Guimaraes (2007)[22] | 12/00-07/03 | Brazil | Puerperal infection among women at 1 maternity hospital, followed until 30 days postpartum using the National Nosocomial Infection Surveillance System. | 5178 | 0.89 | 4 |  |
| Ivanov (2014)[23] | 01/11-12/13 | Bulgaria | Medical record data on puerperal infection at 1 hospital. | 7181 | 9.89 | 3 |  |
| Iyengar (2012)[24] | 01/07-12/10 | India | A field site in rural Rajasthan. Clinical uterine infection diagnosed during home visits by trained nurse-midwives at 2-3 days and 6-9 days postpartum. | 4975 | 1.29 | 4 |  |
| Jokhio (2005)[25] | 05/98-10/98 | Pakistan | Cluster RCT of traditional birth attendant (TBA) training in Larkana District. Lady Health Workers were trained to recognise complications during their routine monthly visits. Women with trained TBA | 9838 | 0.79 | 3 |  |
| Jokhio (2005)[25] | As above | As above | As above; women without trained TBA | 9119 | 4.39 | 3 |  |
| King (2012)[26] | 08/95-02/04 | US | Maternal and Neonatal morbidity using the perinatal database at 1 hospital | 14335 | 2.53 | 5 |  |
| Kovavisarach (2005)[27] | 11/01-02/02 | Thailand | RCT of perineal shaving vs hair cutting on maternal and neonatal outcomes among low-risk women with vaginal delivery at 1 hospital | 458 | 0.00 | 3 |  |
| Magann (2011)[28] | 01/07-07/08 | US | Medical record data on obesity and peripartum complications at 2 hospitals | 4490 | 6.88 | 4 |  |
| Maric (2006)[29] | 1/04-12/04 | Bosnia | Medical record data on puerperal complications until 42 days postpartum in nulliparous women at 1 hospital. Vaginal deliveries | 119 | 1.68 | 2 |  |
| Ngoga (2009)[30] | Start 12/03 | South Africa | Medical record data on pregnancy outcomes in morbidly obese vs a matched sample of normal weight women at 1 hospital. Women with BMI 20-25 | 209 | 0.48 | 2 |  |
| Sanchez (2015)[31] | 01/12-12/13 | Cuba | Maternal age and obstetric complications using medical record data at 1 hospital. Each month, first 30 women aged 25-30 enrolled. | 720 | 1.67 | 2 |  |
| Sanchez (2015)[31] | As above | As above | As above. Each month, the first 15 women over 35 enrolled | 360 | 2.22 | 2 |  |
| Suthee (2007)[32] | 01/99-12/03 | Thailand | Medical record data on meconium-stained amnionitic fluid and maternal infection among low-risk women at 1 hospital | 1079 | 0.93 | 4 |  |
| Peret (2007)[33] | 07/01-09/03 | Brazil | Puerperal morbidity in HIV-infected vs pair-matched non-infected women at 1 hospital; diagnosed before discharge and at a scheduled visit with researchers at 7-15 days postpartum. HIV negative women | 123 | 0.00 | 3 |  |
| Ramírez-Villalobos (2009)[34] | 04/03-12/03 | Mexico | Puerperal complications after hospital discharge among women with vaginal delivery at 1 hospital. Self-reported symptoms collected by trained interviewers at a clinic or home visit at day 7 postpartum | 302 | 2.65 | 3 |  |
| Saizonou (2014)[35] | 07/09-02/10 | Benin | Peripartum infection up to 7 days postpartum at 1 hospital. Diagnosed by doctor or midwife supervised by public health doctor | 1875 | 1.60 | 4 |  |
| Sanabria (2011)[36] | 01/07-12/09 | Cuba | Medical record data on puerperal complications at 1 hospital | 5645 | 0.48 | 1 |  |
| Tabcharoen (2009)[37] | 01/97-12/06 | Thailand | Medical record data on pregnancy outcomes after age 40 at 1 hospital. Women aged 20-34 | 20852 | 0.10 | 4 |  |
| Tabcharoen (2009)[37] | As above | As above | As above; women age 40+ | 792 | 0.38 | 4 |  |
| Winani (2007)[38] | Start 01/2000 | Tanzania | Cord infection and puerperal sepsis with clean delivery kits in 2 rural districts. Home visit at day 5 by village health workers with suspected infection confirmed at health facility | 3262 | 2.12 | 4 |  |

*Results presented are weighted percentage of US population. In meta-analysis we approximated the sample size at 20% for the NIS.[39]

# References

1. Ahnfeldt-Mollerup P, Petersen LK, Kragstrup J, Christensen RD, Sørensen B. Postpartum infections: occurrence, healthcare contacts and association with breastfeeding. Acta Obstetricia et Gynecologica Scandinavica. 2012;91(12):1440-4.

2. Ayzac L, Caillat-Vallet E, Girard R, Chapuis C, Depaix F, Dumas A-M, et al. Decreased rates of nosocomial endometritis and urinary tract infection after vaginal delivery in a French surveillance network, 1997–2003. Infection Control & Hospital Epidemiology. 2008;29(6):487-95.

3. Belfort MA, Clark SL, Saade GR, Kleja K, Dildy III GA, Van Veen TR, et al. Hospital readmission after delivery: evidence for an increased incidence of nonurogenital infection in the immediate postpartum period. American Journal of Obstetrics and Gynecology. 2010;202(1):35. e1-. e7.

4. Benincasa BC, Walker C, Cioba C, Rosa CCdS, Martins DE, Oliveira EDAd, et al. [Rates of infection related to cesarean and vaginal delivery at HCPA]. Revista HCPA Porto Alegre. 2012;32(1):5-9.

5. Bianco A, Roccia S, Nobile CG, Pileggi C, Pavia M. Postdischarge surveillance following delivery: the incidence of infections and associated factors. American Journal of Infection Control. 2013;41(6):549-53.

6. Boccardo J, Manzur A, Duarte N, Yanzon C, Mazzanti A, Paparotti L, et al. [Puerperal endometritis in our setting]. Actual SIDA Infectol. 2013;21(80):48-52.

7. Caughey AB, Stotland NE, Washington AE, Escobar GJ. Maternal and obstetric complications of pregnancy are associated with increasing gestational age at term. American Journal of Obstetrics and Gynecology. 2007;196(2):155. e1-. e6.

8. Cavazos-Rehg PA, Krauss MJ, Spitznagel EL, Bommarito K, Madden T, Olsen MA, et al. Maternal age and risk of labor and delivery complications. Maternal and Child Health Journal. 2015;19(6):1202-11.

9. Cheng YW, Hopkins LM, Laros Jr RK, Caughey AB. Duration of the second stage of labor in multiparous women: maternal and neonatal outcomes. American Journal of Obstetrics and Gynecology. 2007;196(6):585. e1-. e6.

10. Cheng YW, Shaffer BL, Bryant AS, Caughey AB. Length of the first stage of labor and associated perinatal outcomes in nulliparous women. Obstetrics & Gynecology. 2010;116(5):1127-35.

11. Chongsuvivatwong V, Bachtiar H, Chowdhury ME, Fernando S, Suwanrath C, Kor‐anantakul O, et al. Maternal and fetal mortality and complications associated with cesarean section deliveries in teaching hospitals in Asia. Journal of Obstetrics and Gynaecology Research. 2010;36(1):45-51.

12. Darmstadt GL, Hassan M, Balsara ZP, Winch PJ, Gipson R, Santosham M. Impact of clean delivery-kit use on newborn umbilical cord and maternal puerperal infections in Egypt. Journal of Health, Population, and Nutrition. 2009;27(6):746.

13. Dimitriu G. [Clinical statistical study on puerperal sepsis risk factors]. Revista Medico-chirurgicala a Societatii de Medici si Naturalisti din Iasi. 2010;114(1):195-8.

14. Dotters-Katz S, Patel E, Grotegut C, Heine R. Acute infectious morbidity in multiple gestation. Infectious Diseases in Obstetrics and Gynecology. 2015;2015:173261-.

15. Dumas A-M, Girard R, Ayzac L, Beaumont G, Caillat-Vallet E, Depaix F, et al. Effect of intrapartum antibiotic prophylaxis against group B streptococcal infection on comparisons of rates of endometritis and urinary tract infection in multicenter surveillance. Infection Control & Hospital Epidemiology. 2008;29(4):327-32.

16. Ezugwu E, Onah H, Iyoke C, Ezugwu F. Obstetric outcome following free maternal care at Enugu State University Teaching Hospital (ESUTH), Parklane, Enugu, South-eastern Nigeria. Journal of Obstetrics and Gynaecology. 2011;31(5):409-12.

17. Fronczak N, Antelman G, Moran A, Caulfield L, Baqui A. Delivery‐related complications and early postpartum morbidity in Dhaka, Bangladesh. International Journal of Gynecology & Obstetrics. 2005;91(3):271-8.

18. Geller EJ, Wu JM, Jannelli ML, Nguyen TV, Visco AG. Maternal outcomes associated with planned vaginal versus planned primary cesarean delivery. American Journal of Perinatology. 2010;27(09):675-84.

19. Ghani N, Rukanuddin RJ, Ali TS. Prevalence and factors associated with postpartum vaginal infection in the Khyber agency federally administered tribal areas, Pakistan. Journal of Pakistan Medical Association. 2007;57(7):363.

20. Gözüm S, Kiliç D. Health problems related to early discharge of Turkish women. Midwifery. 2005;21(4):371-8.

21. Grotegut CA, Tatineni H, Dandolu V, Whiteman VE, Katari S, Geifman-Holtzman O. Obstetric outcomes with a false-positive one-hour glucose challenge test by the Carpenter-Coustan criteria. The Journal of Maternal-Fetal & Neonatal Medicine. 2008;21(5):315-20.

22. Guimarães EER, Chianca TCM, Oliveira ACd. [Puerperal infection from the perspective of humanized delivery care at a public maternity hospital]. Revista Latino-Americana de Enfermagem. 2007;15(4):536-42.

23. Ivanov S, Tzvetkov K, Kovachev E, Staneva D, Nikolov D. [Puerperal infections after Cesarean section and after a natural childbirth]. Akusherstvo i Ginekologiia. 2014;53:25-8.

24. Iyengar K. Early postpartum maternal morbidity among rural women of Rajasthan, India: a community-based study. Journal of Health, Population, and Nutrition. 2012;30(2):213.

25. Jokhio AH, Winter HR, Cheng KK. An intervention involving traditional birth attendants and perinatal and maternal mortality in Pakistan. New England Journal of Medicine. 2005;352(20):2091-9.

26. King JR, Korst LM, Miller DA, Ouzounian JG. Increased composite maternal and neonatal morbidity associated with ultrasonographically suspected fetal macrosomia. The Journal of Maternal-Fetal & Neonatal Medicine. 2012;25(10):1953-9.

27. Kovavisarach E, Jirasettasiri P. Randomised controlled trial of perineal shaving versus hair cutting in parturients on admission in labor. J Med Assoc Thai. 2005;88(9):1167.

28. Magann EF, Doherty DA, Chauhan SP, Klimpel JM, Huff SD, Morrison JC. Pregnancy, obesity, gestational weight gain, and parity as predictors of peripartum complications. Archives of Gynecology and Obstetrics. 2011;284(4):827-36.

29. Marić T, Tomić V, Darko K. [Puerperal complications in nulliparous women delivered by section caesarean: pair study]. Medicinski Arhiv. 2006;60(4):246-50.

30. Ngoga E, Hall D, Mattheyse F, Grové D. Outcome of pregnancy in the morbidly obese woman. South African Family Practice. 2009;51(1).

31. Sánchez JMB, Serrano YP, Soler JRM. [Advanced maternal age as a conducive element in obstetric complications and birth]. Revista de Ciencias Médicas de Pinar del Río. 2015;19(5):789-802.

32. Suthee Panichkul M, Boonprasert K, Komolpis S, Panichkul P. The association between meconium-stained amniotic fluid and chorioamnionitis or endometritis. J Med Assoc Thai. 2007;90(3):442-7.

33. Péret FJA, Melo VH, Paula LBd, Andrade BAMd, Pinto JA. [Puerperal morbidity in HIV-infected and non-infected women]. Revista Brasileira de Ginecologia e Obstetricia. 2007;29(5):260-6.

34. Ramírez-Villalobos D, Hernández-Garduño A, Salinas A, González D, Walker D, Rojo-Herrera G, et al. [Early postpartum discharge and complications in the early puerperium]. Salud Pública de México. 2009;51(3):212-8.

35. Saizonou J, Ouédraogo L, Paraiso MN, Ayélo P, Kpozèhouen A, Daraté R, et al. [Epidemiology and management of intrapartum infections in the maternity ward of Ouémé-Plateau county hospital in Benin]. The Pan African Medical Journal. 2014;17:89-.

36. Sanabria Fromherz ZE, Fernández Arenas C. [Pathologic behavior of puerperium] Revista Cubana de Obstetrícia y Ginecologia. 2011;37(3):330-40.

37. Tabcharoen C, Pinjaroen S, Suwanrath C, Krisanapan O. Pregnancy outcome after age 40 and risk of low birth weight. Journal of Obstetrics and Gynaecology. 2009;29(5):378-83.

38. Winani S, Wood S, Coffey P, Chirwa T, Mosha F, Changalucha J. Use of a clean delivery kit and factors associated with cord infection and puerperal sepsis in Mwanza, Tanzania. Journal of Midwifery & Women's Health. 2007;52(1):37-43.

39. The Healthcare Cost and Utilization Project (HCUP). Overview of the National (Nationwide) Inpatient Sample (NIS) 2016. Available from: <https://unstats.un.org/sdgs/indicators/regional-groups/>.
