# Supplementary material for: Incidence of maternal peripartum infection: A systematic review and meta-analysis
Source: PLoS Med. 2019 Dec 10;16(12):e1002984. doi: 10.1371/journal.pmed.1002984 (PMC6903710; doi:10.1371/journal.pmed.1002984)
Supplement: S4 Table — (DOCX) [file pmed.1002984.s007.docx]

**S4 Table: Studies of Wound infection**

| **Author** | **Date** | **Country** | **Description** | **Total women** | **Wound Infection**  **(%)** | **Quality** |
| --- | --- | --- | --- | --- | --- | --- |
| Ahnfeldt-Mollerup (2012)[1] | 05/07-04/08 | Denmark | Questionnaire sent to women 28 days after delivering at 1 regional hospital. Report of infection validated with data from General Practice and hospital records. | 1616 | 3.16 | 2 |
| Awan (2015)[2] | 10/10-09/11 | Pakistan | Feto-maternal outcomes in overweight versus normal weight in 1 hospital. Data source unclear. Results for normal weight (18.5-24.9) | 100 | 2.00 | 0 |
| Bailit (2006)[3] | 01/01-12/01 | US | Study of quality of obstetric care. Birth certificate record data from California | 431125 | 0.20 | 4 |
| Bianco (2013)[4] | 09/07-09/08 | Italy | Telephone calls with women at 30 days after delivery at 1 hospital. Postpartum infections corroborated by hospital and physician visits, wound cultures and antibiotic prescriptions. | 1656 | 3.08 | 3 |
| Bodner (2011)[5] | 11/05-01/09 | Austria | Maternal and neonatal outcomes for elective caesarean and planned vaginal delivery. Data source unclear. Low-risk women at 1 hospital. Planned vaginal deliveries only | 178 | 1.12 | 2 |
| Charrier (2010)[6] | 05/04-10/04 | Italy | Study of clean versus sterile vaginal delivery at 2 hospitals. Signs of perineal infection in hospital from direct observation and medical records. Telephone interview at 20-30 days postpartum for reported infection diagnosis, symptoms and antibiotic use. | 409 | 0.00 | 4 |
| Chongsuvivatwong (2010)[7] | 09/01-09/04 | 9 Asian countries | Clinical data on maternal and foetal complications collected by checklist until day 5 postpartum in 12 teaching hospitals in Asia. Vaginal deliveries only. | 12591 | 2.57 | 1 |
| Danish (2010)[8] | 05/98-11/99 | Pakistan | Pregnancy outcome in booked versus unbooked women at 1 hospital. Data collection poorly described. | 322 | 6.21 | 0 |
| Dasgupta (2014)[9] | 10/10-09/11 | India | Pregnancy outcomes in obesity at 1 hospital. Data source unclear. Results for normal BMI (<25kg/m2) | 99 | 2.02 | 1 |
| Dimitriu (2010)[10] | 01/06-09/09 | Kuwait | Medical record data of puerperal infection at 1 hospital | 7550 | 0.33 | 2 |
| Dong (2009)[11] | 01/01-11/04 | China | Before-after study of infection prevention control intervention at 1 hospital. Medical record data of perineal and caesarean wound infections in the control group | 12850 | 1.32 | 4 |
| Dong (2010)[12] | 07/08-08/08 | China | Controlled trial of hand washing method for vaginal deliveries at 1 hospital. Perineal infection data collected by the study doctor | 300 | 8.67 | 4 |
| Ezugwu (2011)[13] | 09/08-12/08 | Nigeria | Medical record data on obstetric outcomes, including wound sepsis, at 1 hospital during the period of free maternal care. | 1152 | 8.33 | 1 |
| Geller (2010)[14] | 1995-2005 | US | Medical record data on maternal outcomes and planned mode of birth among nulliparous, low-risk women at 1 hospital. | 4048 | 0.02 | 4 |
| Goff (2013)[15] | 01/08-12/09 | US | Medical record data from the Perspective database; 355 hospitals accounting for approximately 20% of all hospital admission in the US | 1001189 | 0.35 | 4 |
| Guimaraes (2007)[16] | 12/00-07/03 | Brazil | Surgical site and episiotomy infection among women at 1 maternity hospital, followed until 30 days postpartum using the National Nosocomial Infection Surveillance System. | 5178 | 1.95 | 4 |
| Ivanov (2014)[17] | 01/11-12/13 | Bulgaria | Medical record data on puerperal infection at 1 hospital. Results for perineal wound infection after vaginal delivery | 3897 | 4.29 | 3 |
| Iyengar (2012)[18] | 01/07-12/10 | India | A field site in rural Rajasthan. Perineal wound infection diagnosed during home visits by trained nurse-midwives at 2-3 days and 6-9 days postpartum. | 4975 | 0.42 | 4 |
| Jaleel (2009)[19] | 01/06-04/08 | Pakistan | Pregnancy outcomes in obesity at 1 private maternity home. Data source unclear. Results for control group (BMI 18.5-22.9) | 118 | 0.00 | 1 |
| Janssen (2009)[20] | 01/00-12/04 | Canada | Medical record data. Low risk women in British Colombia planning to delivery with a midwife at home or hospital | 7641 | 0.14 | 3 |
| Janssen (2009)[20] | 01/00-12/05 | Canada | As above. Low risk women planning to delivery with a physician in hospital | 5331 | 0.30 | 3 |
| Kovavisarach (2005)[21] | 11/01-02/02 | Thailand | RCT of perineal shaving versus hair cutting on maternal and neonatal outcomes in low-risk women with vaginal delivery at 1 hospital. Perineal wound infection. Unclear if up to day 4 or 42 | 458 | 8.73 | 3 |
| Latif (2013)[22] | 01/00-06/00 | Bangladesh | Medical record data of outcomes in primigravidae at 1 hospital | 500 | 3.00 | 3 |
| Leth (2009)[23] | 01/01-12/05 | Denmark | Wound infection up to 30 days postpartum identified through the laboratory system, regional prescription database and National Hospital Registry. All deliveries in County of Aarhus | 32468 | 1.78 | 4 |
| Liu (2010)[24] | 01/05-12/06 | China | Clinical study data on abdominal and perineal wound infection and body mass index at 1 hospital. Results for BMI<25 | 327 | 8.87 | 3 |
| Ngoga (2009)[25] | 12/03 | South Africa | Medical record data on pregnancy outcomes in morbidly obese vs a matched sample of normal weight women at 1 hospital. Women with BMI 20-25 | 209 | 0.00 | 2 |
| Oladapo (2007)[26] | 01/90-12/05 | Nigeria | Medical record data on wound infection. Vaginal deliveries at 1 hospital | 656 | 5.18 | 3 |
| Petter (2013)[27] | 01/09-12/10 | Brazil | Medical record data on episiotomy and caesarean wound infections among women at 1 hospital | 9528 | 1.24 | 5 |
| Ramírez-Villalobos (2009)[28] | 04/03-12/03 | Mexico | Episiotomy infection after hospital discharge among women with vaginal delivery at 1 hospital. Self-reported symptoms collected by trained interviewers at a clinic or home visit at day 7 postpartum | 303 | 10.89 | 3 |
| Shriraam (2012)[29] | 11/08-02/09 | India | Self-reported wound infection up to 42 days postpartum using pre-tested questionnaire at up to 6 months after delivery. All women delivered in previous 6 months in rural community of Tamil Nadu | 365 | 2.74 | 2 |

# References

1. Ahnfeldt-Mollerup P, Petersen LK, Kragstrup J, Christensen RD, Sørensen B. Postpartum infections: occurrence, healthcare contacts and association with breastfeeding. Acta Obstetricia et Gynecologica Scandinavica. 2012;91(12):1440-4.

2. Awan S, Bibi S, Makhdoom A, Farooq S, SM T, Qazi RA. Adverse fetomaternal outcome among pregnant overweight women. Pakistan Journal of Medical Sciences. 2015;31(2):383.

3. Bailit JL, Love TE, Dawson NV. Quality of obstetric care and risk-adjusted primary cesarean delivery rates. American Journal of Obstetrics and Gynecology. 2006;194(2):402-7.

4. Bianco A, Roccia S, Nobile CG, Pileggi C, Pavia M. Postdischarge surveillance following delivery: the incidence of infections and associated factors. American Journal of Infection Control. 2013;41(6):549-53.

5. Bodner K, Wierrani F, Grünberger W, Bodner-Adler B. Influence of the mode of delivery on maternal and neonatal outcomes: a comparison between elective cesarean section and planned vaginal delivery in a low-risk obstetric population. Archives of Gynecology and Obstetrics. 2011;283(6):1193-8.

6. Charrier L, Serafini P, Chiono V, Rebora M, Rabacchi G, Zotti CM. Clean and sterile delivery: two different approaches to infection control. Journal of Evaluation in Clinical Practice. 2010;16(4):771-5.

7. Chongsuvivatwong V, Bachtiar H, Chowdhury ME, Fernando S, Suwanrath C, Kor‐anantakul O, et al. Maternal and fetal mortality and complications associated with cesarean section deliveries in teaching hospitals in Asia. Journal of Obstetrics and Gynaecology Research. 2010;36(1):45-51.

8. Danish N, Fawad A, Abbasi N. Assessment of pregnancy outcome in primigravida: comparison between booked and un-booked patients. Journal of Ayub Medical College Abbottabad. 2010;22(2):23-5.

9. Dasgupta A, Harichandrakumar K, Habeebullah S. Pregnancy outcome among obese Indians-a prospective cohort study in a tertiary Care Centre in South India. International Journal of Scientific Study. 2014;2(2):13-8.

10. Dimitriu G. [Clinical statistical study on puerperal sepsis risk factors]. Revista Medico-chirurgicala a Societatii de Medici si Naturalisti din Iasi. 2010;114(1):195-8.

11. Dong L. [Management and Monitoring Measure of Nosocomial Infection in Cesarean Section, Normal Delivery, Domiciliary Delivery Integral Delivery Room]. Chinese Journal of Nosocomiology. 2009;19(16):39.

12. Dong L, Wang G. [Effect of hand washing method to maternity and infant]. Chinese Journal of Nosocomiology. 2010;20(15):2257-9.

13. Ezugwu E, Onah H, Iyoke C, Ezugwu F. Obstetric outcome following free maternal care at Enugu State University Teaching Hospital (ESUTH), Parklane, Enugu, South-eastern Nigeria. Journal of Obstetrics and Gynaecology. 2011;31(5):409-12.

14. Geller EJ, Wu JM, Jannelli ML, Nguyen TV, Visco AG. Maternal outcomes associated with planned vaginal versus planned primary cesarean delivery. American Journal of Perinatology. 2010;27(09):675-84.

15. Goff SL, Pekow PS, Avrunin J, Lagu T, Markenson G, Lindenauer PK. Patterns of obstetric infection rates in a large sample of US hospitals. American Journal of Obstetrics and Gynecology. 2013;208(6):456. e1-. e13.

16. Guimarães EER, Chianca TCM, Oliveira ACd. [Puerperal infection from the perspective of humanized delivery care at a public maternity hospital]. Revista Latino-Americana de Enfermagem. 2007;15(4):536-42.

17. Ivanov S, Tzvetkov K, Kovachev E, Staneva D, Nikolov D. [Puerperal infections after Cesarean section and after a natural childbirth]. Akusherstvo i Ginekologiia. 2014;53:25-8.

18. Iyengar K. Early postpartum maternal morbidity among rural women of Rajasthan, India: a community-based study. Journal of Health, Population, and Nutrition. 2012;30(2):213.

19. Jaleel R. Impact of maternal obesity on pregnancy outcome. Journal of Surgery Pakistan (International). 2009;14(1).

20. Janssen PA, Saxell L, Page LA, Klein MC, Liston RM, Lee SK. Outcomes of planned home birth with registered midwife versus planned hospital birth with midwife or physician. CMAJ. 2009;181(6-7):377-83.

21. Kovavisarach E, Jirasettasiri P. Randomised controlled trial of perineal shaving versus hair cutting in parturients on admission in labor. J Med Assoc Thai. 2005;88(9):1167.

22. Latif T, Ali M, Majeed A, Nahar K, Noor Z. Labor outcome of primigravidae in Mymensingh Medical College Hospital. Mymensingh Medical Journal. 2013;22(3):432-7.

23. Leth RA, Møller JK, Thomsen RW, Uldbjerg N, Nørgaard M. Risk of selected postpartum infections after cesarean section compared with vaginal birth: A five‐year cohort study of 32,468 women. Acta Obstetricia et Gynecologica Scandinavica. 2009;88(9):976-83.

24. Liu H, Cui Y. [Relationship between body mass index of pregnant women and postpartum incision infection]. Maternal and Child Health Care of China. 2010;25(18):2485-6.

25. Ngoga E, Hall D, Mattheyse F, Grové D. Outcome of pregnancy in the morbidly obese woman. South African Family Practice. 2009;51(1).

26. Oladapo OT, Lamina MA, SULE‐ODU AO. Maternal morbidity and mortality associated with elective caesarean delivery at a university hospital in Nigeria. Australian and New Zealand journal of obstetrics and gynaecology. 2007;47(2):110-4.

27. Petter CE, Farret TCF, de Souza Scherer J, Antonello VS. [Factors related to surgical site infections after obstetric procedures]. Scientia Medica. 2013;23(1):5.

28. Ramírez-Villalobos D, Hernández-Garduño A, Salinas A, González D, Walker D, Rojo-Herrera G, et al. [Early postpartum discharge and complications in the early puerperium]. Salud Pública de México. 2009;51(3):212-8.

29. Shriraam V, Shah P, Rani M, Palani G, Sathiyasekaran B. Postpartum morbidity and health seeking pattern in a rural community in South India–population based study. Indian Journal of Maternal and Child Health. 2012;14(3):10.
