# Supplementary material for: Incidence of maternal peripartum infection: A systematic review and meta-analysis
Source: PLoS Med. 2019 Dec 10;16(12):e1002984. doi: 10.1371/journal.pmed.1002984 (PMC6903710; doi:10.1371/journal.pmed.1002984)
Supplement: S5 Table — (DOCX) [file pmed.1002984.s008.docx]

**S5 Table: Studies of Sepsis**

| **Author** | **Date** | **Country** | **Description** | **Total women** | **Sepsis (%)** | **Quality** | **Not in meta-analysis** |
| --- | --- | --- | --- | --- | --- | --- | --- |
| Acosta (2013)[1] | 01/05-12/07 | US | Medical record data for all admissions for delivery of a live birth in California. Sepsis coded as septicaemia or sepsis | 1622474 | 0.10 | 5 |  |
| Acosta (2013)[1] | 01/05-12/07 | US | As above. Severe sepsis, also coded as septic shock or sepsis with prolonged length of stay, transfer to intensive care or death. | 1622474 | 0.05 | 5 |  |
| Bauer (2013)[2] | 01/98-12/08 | US | Maternal sepsis during hospitalisation for delivery using NIS data. Sepsis coded as septicaemia or SIRS | 8999852* | 0.03 | 5 |  |
| Bauer (2013)[2] | 01/98-12/08 | US | As above. Severe sepsis coded as sepsis plus organ dysfunction | 8999852* | 0.01 | 5 |  |
| Belfort (2010)[3] | 01/07-12/07 | US | Medical record data on women readmitted with postpartum infection up to 42 days postpartum at 114 hospitals, representative of the US population. | 222751 | 0.01 | 3 | Overlapping data |
| Ben (2007)[4] | 01/99-12/03 | Tunisia | Medical record data on all severe (near-miss) puerperal infection at one hospital using SIRS criteria. | 20071 | 0.08 | 4 |  |
| Callaghan (2008)[5] | 01/91-12/03 | US | Septicaemia and hospital stay of 3+ days using data on delivery hospitalisations from the National Hospital Discharge Survey | 423480 | 0.02 | 5 |  |
| Cape (2013)[6] | 01/00-12/08 | US | Bacteraemia from 7 days before until 30 days after delivery using the microbiology database at one hospital. Restricted to women with a diagnosis of chorioamnionitis, endometritis or wound infection | 78919 | 0.17 | 4 |  |
| Chongsuvivatwong (2010)[7] | 09/01-09/04 | 9 Asian countries | Clinical data on maternal and foetal complications including peritonitis, collected by checklist until day 5 postpartum in 12 teaching hospitals in Asia. Vaginal deliveries only. | 12591 | 0.02 | 1 |  |
| David (2012)[8] | 01/05-12/10 | India | Medical record data on puerperal sepsis during hospitalisation for delivery, in a midwife-run labour room at one urban health centre. | 1194 | 0 | 2 |  |
| Dotters-Katz (2015)[9] | 01/08-12/10 | US | Study of infection in multiple versus single gestation using NIS data. Codes for septicaemia and bacteraemia | 12524118* | 0.07 | 5 |  |
| Goff (2013)[10] | 01/08-12/09 | US | Medical record data from the Perspective database; 355 hospitals accounting for approximately 20% of all hospital admission in the US. Codes for septicaemia, septic shock, bacteraemia, SIRS | 1001189 | 0.13 | 5 | Overlapping data |
| Huda (2012)[11] | 01/08-12/08 | Bangladesh | Medical record data from 30 hospitals on genital infection and signs of shock, from labour until 32 days postpartum | 1927 | 0.88 | 4 |  |
| Ivanov (2014)[12] | 01/11-12/13 | Bulgaria | Medical record data on puerperal infection, including sepsis, at 1 hospital. | 7181 | 0.08 | 3 |  |
| Karolinski (2013)[13] | 06/08-05/09 | Argentina | Medical record data from 25 hospitals in the Perinatal network of Buenos Aires on life-threatening puerperal sepsis until 42 days postpartum | 65033 | 0.04 | 3 |  |
| Knowles (2014)[14] | 01/05-12/12 | Ireland | Medical and laboratory records at 2 maternity hospitals of blood stream infection secondary to genital tract infection until 42 days postpartum | 136897 | 0.11 | 5 |  |
| Kuklina (2008)[15] | 01/98-12/04 | US | Sepsis coded as septicaemia, septic shock or SIRS with/without organ dysfunction during hospitalisation for delivery using NIS data | 28084407 | 0.03 | 5 | Overlapping data |
| Leth (2009)[16] | 01/01-12/05 | Denmark | Blood stream infection up to 30 days postpartum identified through the laboratory system, regional prescription database and National Hospital Registry. All deliveries in County of Aarhus | 32468 | 0.06 | 5 |  |
| Luz (2008)[17] | 10/05-07/06 | Brazil | Positive blood culture and SIRS or organ dysfunction, collected from medical records during admission for delivery at one hospital | 2207 | 0.05 | 5 |  |
| Lyndon (2012)[18] | 01/05-12/07 | US | Medical record data of maternal sepsis from all live singleton births at hospitals in California. | 1572909 | 0.09 | 4 | Overlapping data |
| Maric (2006)[19] | 01/04-12/04 | Bosnia | Medical record data on puerperal sepsis following vaginal delivery until 42 days postpartum in nulliparous women at 1 hospital | 119 | 0 | 2 |  |
| Mayi-Tsonga (2007)[20] | 06/06-12/06 | Gabon | Audit of near-miss at one hospital. Medical record data on septic shock of pelvic origins | 4350 | 0 | 5 |  |
| Pallasmaa (2008)[21] | 01/97-12/97 | Finland | Puerperal sepsis and peritonitis in all singleton births in Finland using the national hospital discharge registry | 57149 | 0.33 | 4 |  |
| Pallasmaa (2008)[21] | 01/02-12/02 | Finland | Puerperal sepsis and peritonitis in all singleton births in Finland using the national hospital discharge registry | 53568 | 0.45 | 4 |  |
| Pallasmaa (2015)[22] | 01/07-12/11 | Finland | Puerperal sepsis, peritonitis and re-operation in all singleton births in Finland using the national hospital discharge registry | 292553 | 0.81 | 4 |  |
| Sanabria (2011)[23] | 01/07-12/09 | Cuba | Medical record data on puerperal complications including sepsis among women delivering at 1 hospital | 5645 | 0.18 | 1 |  |
| Shriraam (2012)[24] | 11/08-02-09 | India | Self-reported puerperal sepsis up to 42 days postpartum using pre-tested questionnaire at up to 6 months after delivery. All women delivered in previous 6 months in rural community of Tamil Nadu | 365 | 3.84 | 2 | Outlier |
| Simoes (2005)[25] | 01/98-12/98 | Germany | Postpartum septicaemia in the Perinatal database for all women delivering in hospitals in Baden-Wurttemberg State. | 103945 | 0.09 | 3 |  |
| Simoes (2005)[25] | 01/01-12/01 | Germany | Postpartum septicaemia in the Perinatal database for all women delivering in hospitals in Baden-Wurttemberg State. | 88874 | 0.23 | 3 |  |
| Tippawan (2014)[26] | 10/10-09/11 | Thailand | Medical record data on puerperal sepsis in all hospital deliveries in the country using the National Health Security Office data | 442818 | 0.11 | 5 |  |
| Zhang (2005)[27] | 01/95-02/98 | 9 European countries | Data collected from medical records on sepsis (infection with SIRS) at the time of birth. Survey usually covered the hospitals in one region of each country for 12 months. | 211264 | 0.07 | 3 |  |

*Results presented are weighted percentage of US population. In meta-analysis we approximated the sample size at 20% for the NIS[28]

# References

1. Acosta CD, Knight M, Lee HC, Kurinczuk JJ, Gould JB, Lyndon A. The continuum of maternal sepsis severity: incidence and risk factors in a population-based cohort study. PLOS One. 2013;8(7):e67175.

2. Bauer ME, Bateman BT, Bauer ST, Shanks AM, Mhyre JM. Maternal sepsis mortality and morbidity during hospitalization for delivery: temporal trends and independent associations for severe sepsis. Anesthesia & Analgesia. 2013;117(4):944-50.

3. Belfort MA, Clark SL, Saade GR, Kleja K, Dildy III GA, Van Veen TR, et al. Hospital readmission after delivery: evidence for an increased incidence of nonurogenital infection in the immediate postpartum period. American Journal of Obstetrics and Gynecology. 2010;202(1):35. e1-. e7.

4. Ben SH, Khoudayer H, Ben HZ, Masmoudi A, Bouguerra B, Sfar R. [Severe maternal morbidity]. Journal de Gynecologie, Obstetrique et Biologie de la Reproduction. 2007;36(7):694-8.

5. Callaghan WM, MacKay AP, Berg CJ. Identification of severe maternal morbidity during delivery hospitalizations, United States, 1991-2003. American Journal of Obstetrics and Gynecology. 2008;199(2):133. e1-. e8.

6. Cape A, Tuomala RE, Taylor C, Puopolo KM. Peripartum bacteremia in the era of group B streptococcus prophylaxis. Obstetrics & Gynecology. 2013;121(4):812-8.

7. Chongsuvivatwong V, Bachtiar H, Chowdhury ME, Fernando S, Suwanrath C, Kor‐anantakul O, et al. Maternal and fetal mortality and complications associated with cesarean section deliveries in teaching hospitals in Asia. Journal of Obstetrics and Gynaecology Research. 2010;36(1):45-51.

8. David K, Pricilla R, Venkatesan S, Rahman S, Sy G, Vijayaselvi R. Outcomes of deliveries in a midwife-run labour room located at an urban health centre: results of a 5-year retrospective study. The Natl Med J India. 2012;25:323-6.

9. Dotters-Katz S, Patel E, Grotegut C, Heine R. Acute infectious morbidity in multiple gestation. Infectious Diseases in Obstetrics and Gynecology. 2015;2015:173261-.

10. Goff SL, Pekow PS, Avrunin J, Lagu T, Markenson G, Lindenauer PK. Patterns of obstetric infection rates in a large sample of US hospitals. American Journal of Obstetrics and Gynecology. 2013;208(6):456. e1-. e13.

11. Huda FA, Ahmed A, Dasgupta SK, Jahan M, Ferdous J, Koblinsky M, et al. Profile of maternal and foetal complications during labour and delivery among women giving birth in hospitals in Matlab and Chandpur, Bangladesh. Journal of Health, Population, and Nutrition. 2012;30(2):131.

12. Ivanov S, Tzvetkov K, Kovachev E, Staneva D, Nikolov D. [Puerperal infections after Cesarean section and after a natural childbirth]. Akusherstvo i Ginekologiia. 2014;53:25-8.

13. Karolinski A, Mercer R, Micone P, Ocampo C, Mazzoni A, Fontana O, et al. The epidemiology of life‐threatening complications associated with reproductive process in public hospitals in Argentina. BJOG: An International Journal of Obstetrics & Gynaecology. 2013;120(13):1685-95.

14. Knowles S, O'sullivan N, Meenan A, Hanniffy R, Robson M. Maternal sepsis incidence, aetiology and outcome for mother and fetus: a prospective study. BJOG: An International Journal of Obstetrics & Gynaecology. 2015;122(5):663-71.

15. Kuklina EV, Whiteman MK, Hillis SD, Jamieson DJ, Meikle SF, Posner SF, et al. An enhanced method for identifying obstetric deliveries: implications for estimating maternal morbidity. Maternal and Child Health Journal. 2008;12(4):469-77.

16. Leth RA, Møller JK, Thomsen RW, Uldbjerg N, Nørgaard M. Risk of selected postpartum infections after cesarean section compared with vaginal birth: A five‐year cohort study of 32,468 women. Acta Obstetricia et Gynecologica Scandinavica. 2009;88(9):976-83.

17. Luz AG, Tiago DB, Silva JCGd, Amaral E. [Severe maternal morbidity at a local reference university hospital in Campinas, São Paulo, Brazil]. Revista Brasileira de Ginecologia e Obstetrícia. 2008;30(6):281-6.

18. Lyndon A, Lee HC, Gilbert WM, Gould JB, Lee KA. Maternal morbidity during childbirth hospitalization in California. The Journal of Maternal-Fetal & Neonatal Medicine. 2012;25(12):2529-35.

19. Marić T, Tomić V, Darko K. [Puerperal complications in nulliparous women delivered by section caesarean: pair study]. Medicinski Arhiv. 2006;60(4):246-50.

20. Mayi-Tsonga S, Meyé J-F, Tagne A, Ndombi I, Diallo T, Oksana L, et al. [Audit of the severe obstetrical morbidity (near miss) in Gabon]. Cahiers d'Etudes et de Recherches Francophones/Santé. 2007;17(2):111-5.

21. Pallasmaa N, Ekblad U, Gissler M. Severe maternal morbidity and the mode of delivery. Acta obstetricia et gynecologica Scandinavica. 2008;87(6):662-8.

22. Pallasmaa N, Ekblad U, Gissler M, Alanen A. The impact of maternal obesity, age, pre-eclampsia and insulin dependent diabetes on severe maternal morbidity by mode of delivery—a register-based cohort study. Archives of Gynecology and Obstetrics. 2015;291(2):311-8.

23. Sanabria Fromherz ZE, Fernández Arenas C. [Pathologic behavior of puerperium] Revista Cubana de Obstetrícia y Ginecologia. 2011;37(3):330-40.

24. Shriraam V, Shah P, Rani M, Palani G, Sathiyasekaran B. Postpartum morbidity and health seeking pattern in a rural community in South India–population based study. Indian Journal of Maternal and Child Health. 2012;14(3):10.

25. Simoes E, Kunz S, Bosing-Schwenkglenks M, Schmahl F. Association between method of delivery and puerperal infectious complications in the perinatal database of Baden-Württemberg 1998–2001. Gynecologic and Obstetric Investigation. 2005;60(4):213-7.

26. Tippawan Liabsuetrakul M, Suchonwanich Y. Birth rates and pregnancy complications in adolescent pregnant women giving birth in the hospitals of Thailand. J Med Assoc Thai. 2014;97(8):785-90.

27. Zhang WH, Alexander S, Bouvier‐Colle MH, Macfarlane A, Group MB. Incidence of severe pre‐eclampsia, postpartum haemorrhage and sepsis as a surrogate marker for severe maternal morbidity in a European population‐based study: the MOMS‐B survey. BJOG: An International Journal of Obstetrics & Gynaecology. 2005;112(1):89-96.

28. The Healthcare Cost and Utilization Project (HCUP). Overview of the National (Nationwide) Inpatient Sample (NIS) 2016. Available from: <https://unstats.un.org/sdgs/indicators/regional-groups/>.
