# Supplementary material for: Incidence of maternal peripartum infection: A systematic review and meta-analysis
Source: PLoS Med. 2019 Dec 10;16(12):e1002984. doi: 10.1371/journal.pmed.1002984 (PMC6903710; doi:10.1371/journal.pmed.1002984)
Supplement: S6 Table — (DOCX) [file pmed.1002984.s009.docx]

# S6 Table: Studies of Maternal Peripartum Infection

| **Author** | **Date** | **Country** | **Description** | **Total Women** | **Maternal Peripartum Infection**  **(%)** | **Quality** | **Not in meta-analysis** |
| --- | --- | --- | --- | --- | --- | --- | --- |
| Al-Ostad (2015)[1] | 01/98-12/08 | US | Risk factors for sepsis mortality using NIS data. Unspecified codes for puerperal infection. | 5338995 | 0.44 | 4 |  |
| Andersson (2011)[2] | 05/09-11/09 | Nigeria | Self-reported symptoms of infection up to 42 days postpartum. Stratified random sampling to provide state-level representation for 2 Nigerian states. | 14890 | 18.11 | 1 | Outlier |
| Avci (2015)[3] | 03/12-03/13 | Turkey | Maternal obesity and perinatal outcomes at one hospital. Definition and data collection methods for postpartum infection not specified. | 931 | 2.36 | 2 |  |
| Bailit (2006)[4] | 01/01-12/01 | US | Birth certificate record data from California. ICD-9 codes for major postpartum infection, postpartum fever, GU tract infection and wound complications | 431125 | 2.08 | 4 |  |
| Bailit (2013)[5] | 03/08-02/11 | US | Medical record data from a stratified random selection of days at 25 hospitals in a network of Maternal-Fetal Medicine Units. Peripartum infection in low-risk women defined as Chorioamnionitis, postpartum endometritis or postpartum wound infection. | 110205 | 5.06 | 4 |  |
| Bakr (2005)[6] | 01/02-06/02 | Egypt | Before-after study of vaginal chlorhexidine intervention and maternal morbidity at one hospital. Medical record data from the pre-intervention period. Postpartum infection defined as puerperal sepsis, or fever plus offensive vaginal discharge, infected wound, retained products of conception or secondary PPH | 2128 | 0.52 | 4 |  |
| Berg (2009)[7] | 01/01-12/05 | US | Maternal morbidity during hospitalisation for labour using the National Hospital Discharge Survey representing all hospital deliveries in the US. ICD-9 codes for major puerperal infection | 19986000* | 0.50 | 4 | Overlapping data |
| Berg (2009)[7] | 01/93-12/97 | US | As above | 19081000* | 0.80 | 4 |  |
| Chen (2014)[8] | 2011 | China | Random sample of 250 medical records of low-risk deliveries at one hospital. Textbook definition of puerperal infection | 250 | 4.00 | 4 |  |
| Dong (2010)[9] | 07/08-08/08 | China | Controlled trial of hand washing method for low-risk vaginal deliveries at 1 hospital. Data collected by study doctor on puerperal infection (undefined) | 300 | 5.67 | 4 |  |
| Galyean (2009)[10] | 07/02-12/03 | US | Multiparous women with live singleton delivery at four hospitals in California. Serious post-partum infections requiring aminoglycosides from a perinatal outcomes database | 10654 | 2.87 | 3 |  |
| Gibson (2014)[11] | 01/02-12/08 | US | Outcomes in elective induction of low-risk pregnancies at 12 clinical centres and 19 hospitals. Medical record data on infection; intrapartum fever, chorioamnionitis, endomyometritis and wound separation | 96266 | 9.06 | 3 |  |
| Goff (2013)[12] | 01/08-12/09 | US | Medical record data from the Perspective database; 355 hospitals accounting for approximately 20% of all hospital admissions in the US. ICD-9 codes for chorioamnionitis and major puerperal infection | 1001189 | 2.05 | 5 |  |
| Guendelman (2006)[13] | 01/96-12/98 | US | Database of birth certificate and hospital discharge records for 93% of deliveries in California. ICD-9 codes for major puerperal infection. | 1507275 | 0.90 | 5 |  |
| Harrison (2015)[14] | 01/10-12/13 | 6 LMICs | 7 rural communities in Argentina, Guatemala, India, Kenya, Pakistan and Zambia, under the Global Network. Undefined postpartum maternal infection from medical records and a study visit at 42 days | 263648 | 0.67 | 3 |  |
| Jin (2011)[15] | 03/05-03/10 | China | Study of gestational diabetes in one hospital. Undefined puerperal infection collected in a sample of women without diabetes for a single-facility study of gestational diabetes | 192 | 2.08 | 3 |  |
| Karlstrom (2013)[16] | 01/97-12/06 | Sweden | Register of all facility births in the country. Postpartum infection (undefined) after spontaneous onset of labour at term | 13774 | 1.13 | 4 |  |
| Kovavisarach (2010)[17] | 11/06-12/07 | Thailand | Women aged 20-34 delivering at one hospital. Puerperal infection with undefined definition or data collection methods. | 750 | 0.13 | 2 |  |
| Kuklina (2008)[18] | 01/98-12/04 | US | NIS database. ICD-9 codes for puerperal infection and pyrexia of unknown origin. | 28084407* | 0.52 | 5 |  |
| Kyser (2012)[19] | 01/06-12/06 | US | Medical record data from 1045 hospitals in 11 states. Undefined postpartum infection using ICD-9 codes | 1678809 | 0.72 | 4 | Overlapping data |
| Laws (2014)[20] | 01/01-12/09 | Australia | Undefined postpartum infection from linked birth records and hospital admission records up to 1 year postpartum. Women intending to deliver at 8 birthing centres | 14707 | 1.04 | 4 |  |
| Laws (2014)[20] | 01/01-12/09 | Australia | As above. Women intending to deliver at 8 co-located hospitals | 29414 | 1.43 | 3 |  |
| Liu (2007)[21] | 04/91-03/05 | Canada | Low-risk planned vaginal deliveries at all acute-care hospitals in Canada, excluding Quebec and Manitoba. Medical record data of major puerperal infection from ICD-9 codes. | 2292420 | 0.21 | 5 |  |
| Lyndon (2012)[22] | 01/05-12/07 | US | Medical record data of livebirths at hospitals in California. Unspecified ICD-9 codes for maternal infection | 1572909 | 2.75 | 4 | Overlapping data |
| Mandal (2010)[23] | 01/06-12/08 | India | Maternal obesity and pregnancy outcome at one hospital. Combined endometrial and wound infection at 6 weeks postpartum visit in low-risk non-obese women | 422 | 3.79 | 1 |  |
| Ngoc (2005)[24] | 01/01-07/01 | Vietnam | Clinical data collected at 6-week postpartum study visit after vaginal delivery at two hospitals. Serious postpartum infection defined as physician-diagnosed sepsis or clinical symptoms of endometritis, pelvic abscess, or chorioamnionitis | 978 | 4.81 | 3 |  |
| Okumura (2014)[25] | 01/00-12/00 | Peru | Perinatal Information System database from one hospital. ICD-10 codes for puerperal infection | 67693 | 2.40 | 4 |  |
| Palmer (2015)[26] | 04/10-03/12 | UK | Database of all NHS hospital deliveries. ICD-10 codes for puerperal infection or sepsis within 42 days of birth | 1332835 | 0.83 | 5 |  |
| Tippawan (2014)[27] | 10/10-09/11 | Thailand | Medical record data on puerperal sepsis in all hospital deliveries in the country using the National Health Security Office data. ICD-10 code for other puerperal infection | 442818 | 0.25 | 5 |  |
| Wang (2010)[28] | 01/07-12/08 | China | Medical record data from one hospital. Postpartum intrauterine infection defined as fever, headache, dizziness, abnormal lochia, genital tract or caesarean wound infection. | 2382 | 5.75 | 5 |  |

*Results presented are weighted percentage of US population. In meta-analysis we approximated the sample size at 20% for the NIS[29] and 1% for the NHDS.[30]

# References

1. Al‐Ostad G, Kezouh A, Spence AR, Abenhaim HA. Incidence and risk factors of sepsis mortality in labor, delivery and after birth: Population‐based study in the USA. Journal of Obstetrics and Gynaecology Research. 2015;41(8):1201-6.

2. Andersson N, Omer K, Caldwell D, Dambam MM, Maikudi AY, Effiong B, et al. Male responsibility and maternal morbidity: a cross-sectional study in two Nigerian states. BMC Health Services Research. 2011;11(2):S7.

3. Avcı ME, Şanlıkan F, Celik M, Avcı A, Kocaer M, Göçmen A. Effects of maternal obesity on antenatal, perinatal and neonatal outcomes. The Journal of Maternal-Fetal & Neonatal Medicine. 2015;28(17):2080-3.

4. Bailit JL, Love TE, Dawson NV. Quality of obstetric care and risk-adjusted primary cesarean delivery rates. American Journal of Obstetrics and Gynecology. 2006;194(2):402-7.

5. Bailit JL, Grobman WA, Rice MM, Spong CY, Wapner RJ, Varner MW, et al. Risk-adjusted models for adverse obstetric outcomes and variation in risk-adjusted outcomes across hospitals. American Journal of Obstetrics and Gynecology. 2013;209(5):446. e1-. e30.

6. Bakr AF, Karkour T. Effect of predelivery vaginal antisepsis on maternal and neonatal morbidity and mortality in Egypt. Journal of Women's Health. 2005;14(6):496-501.

7. Berg CJ, MacKay AP, Qin C, Callaghan WM. Overview of maternal morbidity during hospitalization for labor and delivery in the United States: 1993–1997 and 2001–2005. Obstetrics & Gynecology. 2009;113(5):1075-81.

8. Chen L, Liu J, Kang Y, Liu J, Sufeng H. [Relationship between pre-pregnant body mass index and pregnancy growth with maternal and neonatal outcomes]. Chongqing Medicine. 2014;43(10):1178-80.

9. Dong L, Wang G. [Effect of hand washing method to maternity and infant]. Chinese Journal of Nosocomiology. 2010;20(15):2257-9.

10. Galyean A, Lagrew D, Bush M, Kurtzman J. Previous cesarean section and the risk of postpartum maternal complications and adverse neonatal outcomes in future pregnancies. Journal of Perinatology. 2009;29(11):726.

11. Gibson KS, Waters TP, Bailit JL. Maternal and neonatal outcomes in electively induced low-risk term pregnancies. American Journal of Obstetrics and Gynecology. 2014;211(3):249. e1-. e16.

12. Goff SL, Pekow PS, Avrunin J, Lagu T, Markenson G, Lindenauer PK. Patterns of obstetric infection rates in a large sample of US hospitals. American Journal of Obstetrics and Gynecology. 2013;208(6):456. e1-. e13.

13. Guendelman S, Thornton D, Gould J, Hosang N. Obstetric complications during labor and delivery: assessing ethnic differences in California. Women's Health Issues. 2006;16(4):189-97.

14. Harrison MS, Ali S, Pasha O, Saleem S, Althabe F, Berrueta M, et al. A prospective population-based study of maternal, fetal, and neonatal outcomes in the setting of prolonged labor, obstructed labor and failure to progress in low-and middle-income countries. Reproductive Health. 2015;12(2):S9.

15. Jin Z, Chi X, Teng W, Wang X, Xu Q, Wang P, et al. [Sex hormone-binding globulin of gestational diabetes mellitus pregnant women with well-controlled glucose and pregnancy outcomes]. Zhonghua fu Chan ke za zhi. 2011;46(6):422-6.

16. Karlström A, Lindgren H, Hildingsson I. Maternal and infant outcome after caesarean section without recorded medical indication: findings from a Swedish case–control study. BJOG: An International Journal of Obstetrics & Gynaecology. 2013;120(4):479-86.

17. Kovavisarach E, Chairaj S, Tosang K, Asavapiriyanont S, Chotigeat U. Outcome of teenage pregnancy in Rajavithi Hospital. J Med Assoc Thai. 2010;93(1):1.

18. Kuklina EV, Whiteman MK, Hillis SD, Jamieson DJ, Meikle SF, Posner SF, et al. An enhanced method for identifying obstetric deliveries: implications for estimating maternal morbidity. Maternal and Child Health Journal. 2008;12(4):469-77.

19. Kyser KL, Lu X, Santillan DA, Santillan MK, Hunter SK, Cahill AG, et al. The association between hospital obstetrical volume and maternal postpartum complications. American Journal of Obstetrics and Gynecology. 2012;207(1):42. e1-. e17.

20. Laws PJ, Xu F, Welsh A, Tracy SK, Sullivan EA. Maternal morbidity of women receiving birth center care in New South Wales: A matched‐pair analysis using linked health data. Birth. 2014;41(3):268-75.

21. Liu S, Liston RM, Joseph K, Heaman M, Sauve R, Kramer MS. Maternal mortality and severe morbidity associated with low-risk planned cesarean delivery versus planned vaginal delivery at term. CMAJ. 2007;176(4):455-60.

22. Lyndon A, Lee HC, Gilbert WM, Gould JB, Lee KA. Maternal morbidity during childbirth hospitalization in California. The Journal of Maternal-Fetal & Neonatal Medicine. 2012;25(12):2529-35.

23. Mandal D, Manda S, Rakshi A, Dey R, Biswas S, Banerjee A. Maternal obesity and pregnancy outcome: a prospective analysis. The Journal of the Association of Physicians of India. 2011;59:486-9.

24. Ngoc NT, Sloan NL, Thach TS, Liem LK, Winikoff B. Incidence of postpartum infection after vaginal delivery in Viet Nam. Journal of Health, Population and Nutrition. 2005:121-30.

25. Okumura JA, Maticorena DA, Tejeda JE, Mayta-Tristán P. [Teenage pregnancy as a risk factor for obstetric and perinatal complications at a hospital in Lima, Peru]. Revista Brasileira de Saúde Materno Infantil. 2014;14(4):383-92.

26. Palmer WL, Bottle A, Aylin P. Association between day of delivery and obstetric outcomes: observational study. BMJ. 2015;351:h5774.

27. Tippawan Liabsuetrakul M, Suchonwanich Y. Birth rates and pregnancy complications in adolescent pregnant women giving birth in the hospitals of Thailand. J Med Assoc Thai. 2014;97(8):785-90.

28. Wang X, Yu X, Qian X. [Postpartum intrauterine infection: clinical features and preventive measures]. Chinese Journal of Nosocomiology. 2010;20(14):2050-1.

29. The Healthcare Cost and Utilization Project (HCUP). Overview of the National (Nationwide) Inpatient Sample (NIS) 2016. Available from: <https://unstats.un.org/sdgs/indicators/regional-groups/>.

30. Centers for Disease Control and Prevention. National Hospital Discharge Survey. Available from: <https://www.cdc.gov/nchs/nhds/index.htm>.
