# Supplementary material for: Incidence of maternal peripartum infection: A systematic review and meta-analysis
Source: PLoS Med. 2019 Dec 10;16(12):e1002984. doi: 10.1371/journal.pmed.1002984 (PMC6903710; doi:10.1371/journal.pmed.1002984)
Supplement: S7 Table — (DOCX) [file pmed.1002984.s010.docx]

**S7 Table: Quality of 111 included studies**

| **Yes** |  |
| --- | --- |
| **Unclear** |  |
| **No** |  |

| **Author (date)** | **Sampling** | **Coverage** | **Definition** | **Data collection** | **Sufficient detail** |
| --- | --- | --- | --- | --- | --- |
| Abramovici (2014) | • Yes | • No | • Yes | • Yes | • Yes |
| Acosta (2013) | • Yes | • Yes | • Yes | • Yes | • Yes |
| Admaty (2012) | • No | • Unclear | • Yes | • Yes | • No |
| Ahnfeldt-Mollerup (2012) | • Yes | • Yes | • No | • No | • No |
| Al-Ostad (2015) | • Yes | • Yes | • Yes | • Yes | • Yes |
| Andersson (2011) | • No | • Yes | • Unclear | • No | • No |
| Avci (2015) | • Unclear | • Yes | • No | • Unclear | • Yes |
| Ayzac (2008) | • Yes | • Yes | • Yes | • Yes | • Yes |
| Bailit (2006) | • Yes | • Yes | • No | • Yes | • Yes |
| Bailit (2013) | • Yes | • Yes | • No | • Yes | • Yes |
| Bakr (2005) | • Yes | • Yes | • No | • Yes | • Yes |
| Balestena (2015) | • No | • Yes | • No | • Yes | • No |
| Bauer (2013) | • Yes | • Yes | • Yes | • Yes | • Yes |
| Bear (2016) | • Yes | • Yes | • Yes | • Yes | • No |
| Belfort (2010) | • Yes | • Yes | • No | • Yes | • No |
| Ben (2007) | • Yes | • Yes | • Yes | • Yes | • No |
| Benincasa (2012) | • Yes | • Yes | • No | • Yes | • No |
| Berg (2009) | • Yes | • Yes | • Yes | • Yes | • No |
| Bianco (2013) | • Unclear | • Yes | • Yes | • Unclear | • Yes |
| Bleich (2012) | • Yes | • Yes | • No | • Yes | • Yes |
| Boccardo (2013) | • Yes | • Yes | • Yes | • Yes | • Yes |
| Bodner (2011) | • Unclear | • Yes | • No | • Unclear | • Yes |
| Borders (2012) | • Unclear | • Yes | • No | • Yes | • Yes |
| Braun (2015) | • Yes | • Yes | • Yes | • Yes | • Yes |
| Butchon (2014) | • Yes | • Yes | • Yes | • Yes | • Yes |
| Callaghan (2008) | • Yes | • Yes | • Yes | • Yes | • Yes |
| Cape (2013) | • Yes | • Yes | • Yes | • Yes | • No |
| Caughey (2007) | • Yes | • Yes | • Yes | • Yes | • Yes |
| Cavazos-Rehg (2015) | • Yes | • Yes | • Unclear | • Yes | • Yes |
| Charrier (2010) | • Yes | • Yes | • Yes | • Yes | • No |
| Cheng (2007) | • Yes | • Yes | • Unclear | • Yes | • Yes |
| Cheng (2010) | • Yes | • Yes | • Yes | • Yes | • Yes |
| Chongsuvivatwong (2010) | • Unclear | • Unclear | • Unclear | • Yes | • No |
| Danilack (2015) | • Unclear | • Yes | • No | • Yes | • No |
| Danish (2010) | • Unclear | • Unclear | • No | • Unclear | • No |
| Darmstadt (2009) | • Yes | • Yes | • Yes | • Yes | • Unclear |
| Dasgupta (2014) | • Unclear | • No | • No | • Unclear | • Yes |
| David (2012) | • Yes | • Yes | • No | • Unclear | • No |
| Debasmita (2010) | • No | • Unclear | • No | • Yes | • No |
| Dimitriu (2010) | • Yes | • Yes | • No | • Unclear | • No |
| dong (2009) | • Yes | • Yes | • No | • Yes | • Yes |
| Dong (2010) | • Unclear | • Yes | • Yes | • Yes | • Yes |
| Dotters-Katz (2015) | • Yes | • Yes | • Yes | • Yes | • Yes |
| Dumas (2008) | • Yes | • Yes | • Yes | • Unclear | • Yes |
| Edwards (2015) | • Yes | • Yes | • Yes | • Yes | • Yes |
| Escosteguy (2013) | • Yes | • Yes | • Yes | • Yes | • Yes |
| Ezugwu (2011) | • Yes | • No | • No | • Unclear | • No |
| Fassett (2013) | • Yes | • Yes | • Yes | • Yes | • No |
| Fronczak (2005) | • Yes | • No | • Yes | • No | • Yes |
| Galyean (2009) | • Yes | • Yes | • No | • Yes | • No |
| Geller (2010) | • Yes | • Yes | • No | • Yes | • Yes |
| Getahun (2010) | • Unclear | • Yes | • Yes | • Yes | • No |
| Gibson (2014) | • Yes | • No | • No | • Yes | • Yes |
| Goff (2013) | • Yes | • Yes | • No | • Yes | • Yes |
| Gozum (2005) | • Unclear | • No | • No | • No | • Yes |
| Grotegut (2008) | • Unclear | • Yes | • Yes | • Yes | • Yes |
| Guendelman (2006) | • Yes | • Yes | • Yes | • Yes | • Yes |
| Guimaraes (2007) | • Yes | • Yes | • Yes | • Unclear | • Yes |
| Harrison (2015) | • Yes | • Yes | • No | • Unclear | • Yes |
| Huda (2012) | • Yes | • Yes | • Yes | • Yes | • No |
| Ivanov (2014) | • Yes | • Yes | • No | • Yes | • No |
| Iyengar (2012) | • Yes | • Yes | • No | • Yes | • Yes |
| Jaleel (2009) | • No | • Unclear | • No | • Unclear | • Yes |
| Janssen (2009) | • Yes | • Yes | • Unclear | • Unclear | • Yes |
| Jin (2011) | • Unclear | • Yes | • No | • Yes | • Yes |
| Jokhio (2005) | • Yes | • Yes | • No | • No | • Yes |
| Karlstrom (2013) | • Yes | • Yes | • No | • Yes | • Yes |
| Karolinski (2013) | • Unclear | • Yes | • Yes | • Yes | • Unclear |
| King (2012) | • Yes | • Yes | • Yes | • Yes | • No |
| Knowles (2014) | • Yes | • Yes | • Yes | • Yes | • Yes |
| Kovavisarach (2005) | • Unclear | • Yes | • Unclear | • Yes | • No |
| Kovavisarach (2010) | • Unclear | • Yes | • No | • Unclear | • Yes |
| Kuklina (2008) | • Yes | • Yes | • Yes | • Yes | • Yes |
| Kyser (2012) | • Yes | • Yes | • Unclear | • Yes | • Yes |
| Latif (2013) | • Yes | • Yes | • No | • Unclear | • Yes |
| Laws (2014) | • Unclear | • Yes | • Unclear | • Unclear | • Yes |
| Leth (2009) | • Yes | • Yes | • No | • Yes | • Yes |
| Liu (2007) | • Yes | • Yes | • Yes | • Yes | • Yes |
| Liu (2010) | • Unclear | • Yes | • Yes | • Yes | • No |
| Lulu (2014) | • Unclear | • Yes | • Yes | • Yes | • Yes |
| Luz (2008) | • Yes | • Yes | • Yes | • Yes | • Yes |
| Lyndon (2012) | • Yes | • Yes | • Unclear | • Yes | • Yes |
| Magann (2008) | • Yes | • Yes | • Unclear | • Yes | • Yes |
| Magann (2011) | • Yes | • Yes | • Unclear | • Yes | • Yes |
| Malloy (2014) | • Yes | • Yes | • Unclear | • Yes | • Yes |
| Maric (2006) | • No | • Yes | • No | • Yes | • No |
| Matsuda (2011) | • Yes | • Unclear | • Yes | • Yes | • Yes |
| Mayi-Tsonga (2007) | • Yes | • Yes | • Yes | • Yes | • Yes |
| Nasreen (2007) | • Yes | • Yes | • No | • No | • Yes |
| Nelson (2014) | • Yes | • Yes | • No | • Yes | • Yes |
| Ngoc (2005) | • Unclear | • Yes | • Unclear | • Yes | • Yes |
| Ngoga (2009) | • No | • Yes | • Unclear | • Unclear | • Yes |
| Okumura (2014) | • Yes | • Yes | • No | • Yes | • Yes |
| Oladapo (2007) | • No | • Yes | • No | • Yes | • Yes |
| Osmundson (2011) | • Yes | • Yes | • No | • Unclear | • Yes |
| Pallasmaa (2008) | • Yes | • Yes | • No | • Yes | • Yes |
| Pallasmaa (2015) | • Yes | • Yes | • No | • Yes | • Yes |
| Palmer (2015) | • Yes | • Yes | • Yes | • Yes | • Yes |
| Panichkul (2007) | • Yes | • Yes | • No | • Yes | • Yes |
| Peret (2007) | • No | • Yes | • No | • Yes | • Yes |
| Ramírez-Villalobos (2009) | • No | • No | • Yes | • Yes | • Yes |
| Saizonou (2014) | • Yes | • Yes | • No | • Yes | • Yes |
| Sanabria (2011) | • Unclear | • Yes | • No | • Unclear | • No |
| Shah (2011) | • Unclear | • Yes | • Unclear | • Yes | • No |
| Shazia (2015) | • No | • Unclear | • No | • No | • No |
| Shriraam (2012) | • Unclear | • Yes | • No | • No | • Yes |
| Simoes (2005) | • Yes | • Yes | • Unclear | • Yes | • No |
| Tabcharoen (2009) | • Yes | • Yes | • No | • Yes | • Yes |
| Wang (2010) | • Yes | • Yes | • Yes | • Yes | • Yes |
| Winani (2007) | • Unclear | • Yes | • Yes | • Yes | • Yes |
| Zhang (2005) | • Yes | • Yes | • Yes | • Unclear | • No |
