# Supplementary figures and images for: Incidence of maternal peripartum infection: A systematic review and meta-analysis
Source: PLoS Med. 2019 Dec 10;16(12):e1002984. doi: 10.1371/journal.pmed.1002984 (PMC6903710; doi:10.1371/journal.pmed.1002984)

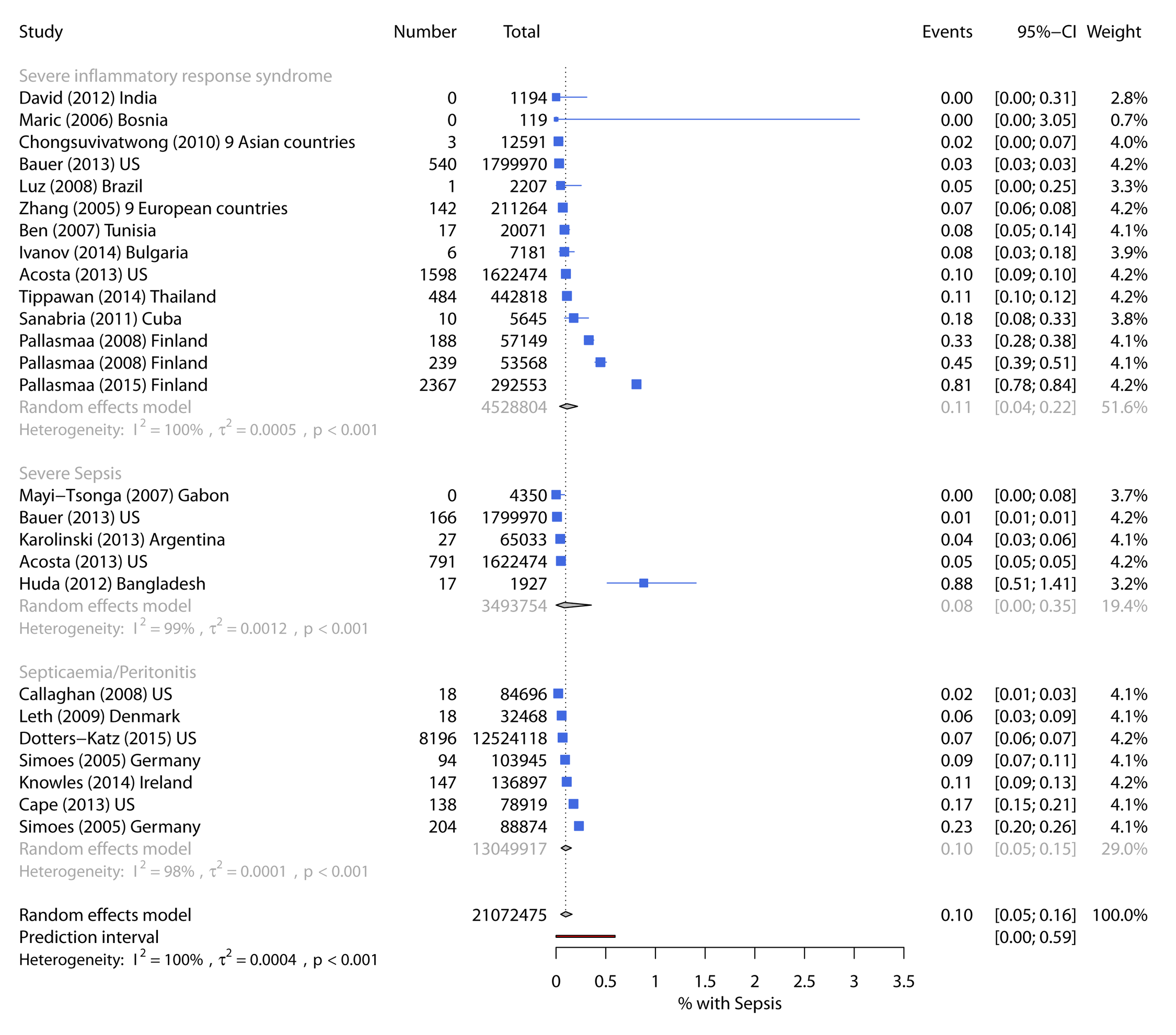

Supplement: S1 Fig — (TIF) [file pmed.1002984.s011.tif]
