# Supplementary material for: Incidence of maternal peripartum infection: A systematic review and meta-analysis
Source: PLoS Med. 2019 Dec 10;16(12):e1002984. doi: 10.1371/journal.pmed.1002984 (PMC6903710; doi:10.1371/journal.pmed.1002984)
Supplement: Attachment — Submitted filename: woodd.pdf [file pmed.1002984.s012.pdf]

This paper presents a systematic review with meta–analysis of studies of maternal peripartum infection.

I was asked for a statistical report and I interpret that to include all aspects of the design and conduct of the study.

Points of detail

Page 6 It might be good to clarify why pre–1990 studies are seen as no longer relevant.

Page 7 Does PL want to be LP to be consistent with the author list? I know Chinese naming causes problems for Anglophones like me but consistency is good.

Page 11 It is good to see such a wide range of languages covered. Can I suggest that these are also marked in the bibliography section? At the moment some titles are in the original, some not. Perhaps put the English titles in brackets or put the language in parentheses after the title?

Pages 14 to 16 I wonder whether this table might be better in the supplement? The results are summarised in the main text.

Page 16 Has study extent been defined previously? I could not find it in the methods and I did not grasp its meaning first time through.

It would be good to cite the particular package used in R. You, or more strictly LSHTM, are paying Stata in cash, but citation is the only payment R authors get. Disclaimer: I am an R package author although not of the one the authors are using.

Points of more substance

Meta–regression

It is not clear to me (Page 31) how collinearity comes in to the analyses as a limitation. Does this mean that the authors would have done meta–regression with multiple moderators but were thwarted? As far as I can see all the meta–regressions presented are using a single moderator. I am not saying an analysis using multiple moderators should have been, even with

this number of studies there are limits to ambition. Can te authors clarify what was done and where the collinearity comes in?

More importantly why do the Table from Table 5 onwards show different moderators? If a moderator analysis was attempted then I think it should be presented. A footnote would do if the analysis could not even be contemplated because, for instance, all the studies fell into the level of a categorical moderator.

Heterogeneity

Usually I would have suggested giving confidence intervals for I^2 but given the very high values this seems unnecessary. It is correct as the authors state on page 11 to base the decision to use fixed or random effects on scientific and clinical theory not on the observed level of heterogeneity.

The high levels of heterogeneity do raise questions though. One thing which the authors have not, as far as I can see, taken into account here is the precision of the individual primary studies. As Rücker et al. (2008) point out if the primary studies are very large, or very precise for other reasons, then I^2 is almost bound to be large. It might be better to supplement I^2 with values of τ^2.

A further issue here is that the high levels of heterogeneity mean that the studies have effectively equal weight. Looking at Figure 2 for example this means that the 102 women in Osmundson get almost as much weight as the 10458616 in Danilack. This may not matter but if there is any evidence of small study effects it would be troubling. In the presence of this much heterogeneity it is hard to detect small study effects either by funnel plots or by regression tests which is unfortunate. Do the authors have any feeling from their knowledge of the primary studies that this might be an issue?

I would also consider whether to supplement the presentation with prediction intervals as suggested by Riley et al. (2011). There is nothing wrong with confidence intervals about the summary estimate but prediction intervals add something extra. Since the authors have used R they can obtain these with metafor and probably other packages too. In this case one might argue that what is needed is prediction about what would be seen in a new setting rather than having an estimate about the mean of the global distribution. The former is what a prediction interval would provide.

Summary

Points for clarification and suggestion about some additional analysis.

Michael Dewey

References

R D Riley, J P T Higgins, and J J Deeks. Interpretation of random effects meta–analyses. *British Medical Journal*, 342:964–967, 2011.

G Rücker, G Schwarzer, J R Carpenter, and M Schumacher. Undue reliance on I^2 in assessing heterogeneity may mislead. *BMC Medical Research Methodology*, 8(79), 2008.
