# Supplementary material for: Incidence of maternal peripartum infection: A systematic review and meta-analysis
Source: PLoS Med. 2019 Dec 10;16(12):e1002984. doi: 10.1371/journal.pmed.1002984 (PMC6903710; doi:10.1371/journal.pmed.1002984)
Supplement: Attachment — Submitted filename: Response to reviewers.docx [file pmed.1002984.s013.docx]

Dear Editors,

Thank you for the very helpful and productive comments from yourselves and the reviewers.

We have attempted to address all of these in the responses below. Original questions are on the left and our response is on the right. Text copied from the updated article is highlighted.

| General comments |  |
| --- | --- |
| Please add a space before reference brackets. | DONE |
| Please add full stops after the reference brackets at the end of the sentences. | DONE |
|  |  |
| Title |  |
| Please omit the word ‘literature’ | DONE |
|  |  |
| Abstract |  |
| Please report your abstract according to PRISMA for abstracts, following the PLOS Medicine abstract structure (Background, Methods and Findings, Conclusions) http://www.plosmedicine.org/article/info:doi/10.1371/journal.pmed.1001419 | See below. We have also added a sentence on assessing risk of bias/quality |
|  |  |
| Abstract - Background |  |
| Provide the context of why the study is important. The final sentence should clearly state the study question. | Important of the study and study question added  Infection is an important, preventable cause of maternal morbidity and pregnancy-related sepsis accounts for 11% of maternal deaths. However, frequency of maternal infection is poorly described and to our knowledge it remains the one major cause of maternal mortality without a systematic review of incidence. Our objective was to estimate the global incidence of maternal peripartum infection. |
| Please avoid assertions of primacy. Please add ‘to our knowledge’ to your final sentence. | We have added the statement “to our knowledge” |
|  |  |
| Abstract - Methods & Findings |  |
| Please provide the data sources and types of study designs included (including brief demographic details). | We provide a summary of the main data sources as per PRISMA guidance  We searched Medline, EMBASE, Global Health and five other databases from January 2005 to June 2016  We have added data on study designs and brief demographics  we included 111 studies of infection in women in labour or postpartum from 46 countries. Four studies were randomised controlled trials, two were before-after intervention studies and the remainder were observational cohort or cross-sectional studies. |
| Please ensure that all numbers presented in the abstract are present and identical to numbers presented in the main manuscript text. | We have checked and ensured these are consistent |
| In the last sentence of the Abstract Methods and Findings section, please describe the main limitation(s) of the study's methodology. | We have added a sentence with the main methodological limitations. Interpretation is further limited by the inclusion of studies that were not conducted at population-level and those restricted to low-risk groups of women |
| Please capitalise PROSPERO | DONE |
| Please specify that numbers in brackets are 95% CI | DONE |
|  |  |
| Abstract - Conclusions |  |
| Please begin your conclusion with ‘In this study, we observed …’ or similar | DONE  In this study we observed pooled infection estimates of almost 4% in labour and between 1-2% of each infection outcome postpartum. This indicates maternal peripartum infection is an important complication of childbirth |
|  |  |
| Author Summary |  |
| At this stage, we ask that you include a short, non-technical Author Summary of your research to make findings accessible to a wide audience that includes both scientists and non-scientists. The Author Summary should immediately follow the Abstract in your revised manuscript. This text is subject to editorial change and should be distinct from the scientific abstract. Please see our author guidelines for more information: https://journals.plos.org/plosmedicine/s/revising-your-manuscript#loc-author-summary | We have written and added an author summary. |
|  |  |
| Introduction |  |
| Please specify that numbers in brackets are 95% CI | These are now specified as 95% uncertainty intervals |
| Please define puerperium | We have removed the term puerperium as the definition is not always consistent and relates to involution of the uterus. Instead we specified the intended time period used by WHO in their definition |
| Please explain the need for and potential importance of your study. | The introduction already describes the importance of infection as a cause of maternal morbidity and mortality. As noted above, we have clarified the lack of an existing review and the reliance on modelled estimates of incidence.  We have now expanded the last paragraph of the introduction to explain the importance of studying this particular group of infections  At a time of increased global attention on maternal sepsis this group of infections was chosen as being notable for causing over half the cases of severe maternal sepsis in the UK, for example. In addition, the direct association of maternal peripartum infection with the process of giving birth presents key opportunities for prevention, while protecting the efficacy of antibiotics, of importance amidst growing concerns about antimicrobial resistance [11]. To aid prioritization by decision makers and guide future research we set out to estimate the global incidence of maternal peripartum infection. |
| Indicate whether your study is novel and how you determined that. | Thank you for requesting clarity about this. We have updated paragraph 1 to indicate how novelty was determined:  a review of maternal morbidity identified no published systematic literature review of infection incidence making it the one major direct cause of maternal morbidity without such a review |
| Please conclude the Introduction with a clear description of the study question or hypothesis. | We have added the following to the end of the introduction  we set out to estimate the global incidence of maternal peripartum infection. |
|  |  |
| Methods |  |
| Please update your search to the present time. We require that SRs are updated to within roughly 6 months of the expected publication date. |  |
| Please capitalise PROSPERO | DONE |
| Please report your SR/MA according to the appropriate study design provided at the EQUATOR site. http://www.equator-network.org/?post_type=eq_guidelines&eq_guidelines_study_design=systematic-reviews-and-meta-analyses&eq_guidelines_clinical_specialty=0&eq_guidelines_report_section=0&s=+ | According to the EQUATOR site we should be following PRISMA guidelines which we have done. |
| Please include sufficient text excerpted from the manuscript to explain how you accomplished all applicable checklist items. When completing the checklist, please use section and paragraph numbers from within each section, rather than page numbers. | Done |
| Please refer to the attached PRISMA checklist early in your methods section. | Done |
| Please provide names of all nine databases (as mentioned in your abstract) | We have corrected the abstract to eight databases |
| Please avoid terms like ‘general’ | We have removed the word general |
| Please define JHPIEGO | JHPIEGO was originally Johns Hopkins Program for International Education in Gynecology and Obstetrics, but is now referred to simply as **Jhpiego**. |
|  |  |
| Results |  |
| When a p value is given, please specify the statistical test used to determine it. | We have requested this information from the R author but have not yet heard back. We will update the paper once we hear. |
| Line 225 - please clarify what is meant by ‘Attrition or missing data of >15% was described in 13%‘ | We have changed this to read  In 13% of studies there were attrition or missing data in >15% of observations |
| Line 252 - please define NIS | Now defined in the text as the US National Inpatient Sample |
| Line 313 - please correct ‘severe sepsis with or organ dysfunction’ | Thank you for spotting this. The word ‘or’ has been deleted |
| Line 315 - please define NHDS | Now defined in the text as the US National Hospital Discharge Survey |
|  |  |
| Discussion |  |
| Please present and organize the Discussion as follows: a short, clear summary of the article's findings; what the study adds to existing research and where and why the results may differ from previous research; strengths and limitations of the study; implications and next steps for research, clinical practice, and/or public policy; one-paragraph conclusion. | As requested the discussion starts with a summary of findings, paragraph 2 compares this to existing research, we then discuss limitations of the included studies and risk of bias, strengths and limitations of the review, implications and next steps are included in the conclusion |
| At line 416, please substitute "very" for "extremely". | Done |
|  |  |
| Conclusion |  |
| Please avoid assertions of primacy. Please add ‘to our knowledge’. | Done |
|  |  |
| References |  |
| Please ensure that journal titles are capitalised properly. | Done |
| To references 24, 45 & 66, please full access details (journal number, page numbers or URL, as available). | Page numbers have been added |
|  |  |
|  |  |
|  |  |
| Comments from the reviewers: |  |
|  |  |
| Reviewer #1: This is a worthy paper and, although i'm not an epidemiologist, I have no scientific concersn with the methodology. | Thank you for valuing the worth of this paper |
|  |  |
| My only worry is whether it is of sufficient general interest to justify publication is such a high impact general journal as PLOS medicine. As the authors note, previous estimates of perinatal infection have been around 3%. This review confirms that figure to be broadly correct, although the estimate is very uncertain because of the different ways in which infection has been identified and classified. And there you have it.  IMHO worthy of publication somehwere but not suffciently high priority for PLOS Medicine. | We believe this paper is of importance and interest. Maternal health is a key area of the SDGs and has recently shown to be off target. Maternal infection and sepsis is an area of particular global interest, particularly in light of increased concerns around AMR. It is very important to know the extent of this problem. Previous estimates are based on models. While our estimates are fairly similar they are still very important to support future research and highlight the issue to policy makers and clinicians |
|  |  |
|  |  |
|  |  |
|  |  |
| Reviewer #2: |  |
|  |  |
| This paper presents a systematic review with meta{analysis of studies of maternal peripartum infection. I was asked for a statistical report and I interpret that to include all aspects of the design and conduct of the study. |  |
| Points of detail |  |
| Page 6 It might be good to clarify why pre{1990 studies are seen as no longer relevant. | Infection incidence is likely to have decreased over time. Pre-1990 studies were considered too old and no longer relevant. This clarification has been added to the exclusion criteria.  Data collected before 1990 due to potential decrease in incidence over time. |
| Page 7 Does PL want to be LP to be consistent with the author list? I know Chinese naming causes problems for Anglophones like me but consistency is good. | Thank you, this has been corrected |
| Page 11 It is good to see such a wide range of languages covered. Can I suggest that these are also marked in the bibliography section? At the moment some titles are in the original, some not. Perhaps put the English titles in brackets or put the language in parentheses after the title? | Thank you, we have now put the English translation in brackets for all foreign language papers. In two cases, we translated the titles ourselves |
| Pages 14 to 16 I wonder whether this table might be better in the supplement? The results are summarised in the main text. | We have considered this and decided to go with the reviews suggestion; happy for editors to advise otherwise |
| Page 16 Has study extent been defined previously? I could not find it in the methods and I did not grasp its meaning first time through. | We have defined this in the methods, Data management and analysis, paragraph 2  We created a variable named “study extent” to reflect how nationally representative the study population might be: national level (total population or representative sample), state/regional level, health facility network (e.g. surveillance network or insurance scheme), two or more facilities or field sites, single facility or field site. |
| It would be good to cite the particular package used in R. You, or more strictly LSHTM, are paying Stata in cash, but citation is the only payment R authors get.  Disclaimer: I am an R package author although not of the one the authors are using. | Many thanks for letting us know. We are happy to add the citation |
|  |  |
| Points of more substance |  |
| Meta{regression It is not clear to me (Page 31) how collinearity comes in to the analyses as a limitation. Does this mean that the authors would have done meta{ regression with multiple moderators but were thwarted? As far as I can see all the meta{regressions presented are using a single moderator. I am not saying an analysis using multiple moderators should have been, even with this number of studies there are limits to ambition. Can the authors clarify what was done and where the collinearity comes in? | Thank you for drawing our attention to this. We had planned to conduct meta-regression with multiple moderators but were limited by the number of studies and close correlation of some moderators. We did not formally assess for collinearity but explored correlations between variables. We have altered the text to reflect this. Final sentence in the methods now reads:  World region and variables with evidence of association (p<0.1) are included in multivariable models unless data were sparse or closely correlated. |
| More importantly why do the Table from Table 5 onwards show different moderators? If a moderator analysis was attempted then I think it should be presented. A footnote would do if the analysis could not even be contemplated because, for instance, all the studies fell into the level of a categorical moderator. | In an attempt to simplify the results we only presented moderators with some evidence of association. However, we fully appreciate the importance of presenting all results and have now updated the tables to include all moderators assessed. |
|  |  |
| Heterogeneity Usually I would have suggested giving confidence intervals for I2 but given the very high values this seems unnecessary. It is correct as the authors state on page 11 to base the decision to use fixed or random effects on scientific and clinical theory not on the observed level of heterogeneity. | Thank you |
| The high levels of heterogeneity do raise questions though. One thing which the authors have not, as far as I can see, taken into account here is the precision of the individual primary studies. As Rucker et al. (2008) point out if the primary studies are very large, or very precise for other reasons, then I2 is almost bound to be large. It might be better to supplement I2 with values of tau2. | Thank you for this helpful comment. It is very likely that the high I^2^ reflects the number of large, precise studies more than the extent of between-study heterogeneity. We now mention tau^2^ in both the results and discussion. It is already presented in the forest plots and is usually small. |
| A further issue here is that the high levels of heterogeneity mean that the studies have effectively equal weight. Looking at Figure 2 for example this means that the 102 women in Osmundson get almost as much weight as the 10458616 in Danilack. This may not matter but if there is any evidence of small study effects it would be troubling. In the presence of this much heterogeneity it is hard to detect small study effects either by funnel plots or by regression tests which is unfortunate. Do the authors have any feeling from their knowledge of the primary studies that this might be an issue? | Thank you for this comment. From our knowledge of the studies there does not appear to be a consistent effect of small study size. These studies are more likely to be based at single-facilities, which have their limitations as already discussed. However, they differ in regards to other study characteristics and the very large studies are primarily from US hospital databases which also have their drawbacks.  All studies included in the high-quality estimates had over 1000 women.  As the main issues with study size relate to design characteristics already discussed we have not added to the discussion, but are happy to do so if desired |
| I would also consider whether to supplement the presentation with prediction intervals as suggested by Riley et al. (2011). There is nothing wrong with confidence intervals about the summary estimate but prediction intervals add something extra. Since the authors have used R they can obtain these with metafor and probably other packages too. In this case one might argue that what is needed is prediction about what would be seen in a new setting rather than having an estimate about the mean of the global distribution. The former is what a prediction interval would provide. | Many thanks for this suggestion. We are now including prediction intervals in the results. We have also re-explored the effect of removing outliers on the pooled estimates, tau2 and prediction intervals and as a result have decided that meta-analysis results should be presented without these outliers. The results and text have all been updated accordingly. |
| Summary Points for clarification and suggestion about some additional analysis. Michael Dewey |  |
| References |  |
| R D Riley, J P T Higgins, and J J Deeks. Interpretation of random effects meta{analyses. British Medical Journal, 342:964{967, 2011.  G Rucker, G Schwarzer, J R Carpenter, and M Schumacher. Undue reliance on I2 in assessing heterogeneity may mislead. BMC Medical Research Methodology, 8(79), 2008. |  |
|  |  |
|  |  |
|  |  |
|  |  |
| Reviewer #3: ¬¬¬Review of manuscript P-Medicine 19-02904 |  |
| Thank you for the opportunity to review this manuscript. This is a systematic literature review and meta-analysis on incidence of maternal peripartum infection. The study used random-effects models to obtain pooled estimates for each outcome. Incidence of peripartum infection risk appear to be lower than global estimates. The study used the WHO definition for peripartum infection of the genital tract or surrounding tissues from onset of membrane ruptures to 42 days postpartum. In general, the study is of general interest and of high quality. | Thank you for this excellent summary and for recognising the importance of this work |
| General comments |  |
| 1. Not clear how the outcome "Maternal peripartum infection" is defined. Is it any of the infections studied? An overall measure? Then why is the incidence lower as compared to chorioamnionitis? | Thank you for requesting this clarification. This outcome was used when studies presented a composite of two or more of the other infections, or a broader/less well-defined infection outcome. We have tried to clarify this in the methods:  We included a fifth category, “maternal peripartum infection” for studies with a composite outcome of two or more of the above infection types, and those that used a broader or un-specified definition of infection within the peripartum period.  Many composite outcomes only combined endometritis and wound infection and did not include chorioamnionitis, hence the lower incidence. |
| 2. Infection incidences varies with mode of delivery and the rate of caesarean delivery differ between countries, regions and clinics. Hence, wound infection would benefit from being reported for caesarean deliveries separately. | The aim was to provide population level estimates of all infections, and appreciate that for various reasons, including mode of delivery, we might expect a different estimate in different populations. Wound infection also includes perineal wounds following vaginal delivery. We plan future work to look at studies that separate caesarean section infection but of note, there is a recently published review of infection following caesarean section in Africa by Sway et al which we refer to in the discussion |
| 3. Why were infections in the urinary tract excluded? One could argue that they would be included in the genital tract. | While they could be included in the genitourinary tact, genital tract specifically excludes the urinary tract and therefore they are not part of the WHO definition. They may be related to healthcare, specifically the use of urinary catheters, but are less directly related to childbirth than the other infections. |
| Specific comments |  |
| 1. Would it be possible to provide a table on the ICD-codes used in various studies for assessment of the subgroups of peripartum infections? | Yes, we have added this to the supplementary material. |
| 2. Table 3 appear to be truncated (ends with Liu). | Apologies, this was an error that occurred when pasting the table into the main text. As suggested by another reviewer, we will now include this table in the supplementary material. We have attached it separately to avoid further error. |
| 3. Could the study also assess instrumental deliveries (vacuum and forceps)? | Our objective was to assess infection at population level. We therefore excluded studies that only included a group of women at higher-risk of infection such as those with instrumental deliveries or caesarean section. We also did not attempt to analyse the effect of risk factors on infection. However, we did include studies that involved only low-risk women and attempted to assess the effect of these in the meta-regression. A further review analysing the effect of instrumental deliveries would certainly be of interest. |
|  |  |
| Reviewer #4: Review of PMEDICINE-D-19-0290 |  |
| The authors report a meta-analysis on the incidence of peripartum infection. The subject is important clinically.  The reported incidence of chorioamnionitis, endometritis, wound infection and sepsis are consistent with contemporary literature and useful for counseling and quality assessment/ improvement among centers.  The high heterogeneity in definition of infections is concerning and questions the utility of pooling the data together. I agree with the conclusion that "Better quality research, using standard definitions, is required ….". | Thank you for noting the importance of this subject and agreeing with the need for better quality research. The issue of pooling data is discussed further below |
| Specific points: |  |
| 1. Please justify mixing clinical trials with observational studies. | We have now specified that there are only four trials and two before-after intervention studies. These provide data from Pakistan, Thailand, Egypt, China and the US. Given the paucity of LMIC data we believe it is important to include these in the results and meta-analysis. When there were differences in the outcome, we used the control/comparison arm or the pre-intervention study period. We make the study type clear in the tables of studies so others can reanalyse if they wish. |
| 2. I agree with reporting the random effects models when definitions of infection varied widely, but can the authors justify pooling when the heterogeneity is > 99%? | We appreciate the concern about pooling studies that may not be measuring the same thing. We would argue that as the studies we pool all aim to measure the same outcome e.g. chorioamnionitis or endometritis, within a very similar time period, then it is reasonable to pool estimates. We have added more to the study limitations to reflect this. We have also responded to comments from the statistical reviewer and now present Tau^2^ as a more useful measure of heterogeneity and the prediction intervals to indicate the expected range of a true proportion in a future study. |
| 3. The authors make a good argument in the discussion for not limiting the analyses to studies meeting the WHO criteria for infection, but a sensitivity analyses doing this and reporting the findings could be helpful. | As reported in the discussion, no study met the full WHO definition for maternal peripartum infection. Rather than conducting sensitivity analyses we analysed distinct infection outcomes and explored follow-up time in the meta-regression. |
| Any attachments provided with reviews can be seen via the following link: |  |
| https://www.editorialmanager.com/pmedicine/l.asp?i=489314&l=I0I2WCZ4 |  |
|  |  |
| __________________________________________________ |  |
| In compliance with data protection regulations, you may request that we remove your personal registration details at any time. (Use the following URL: https://www.editorialmanager.com/pmedicine/login.asp?a=r). Please contact the publication office if you have any questions. |  |
