# Supplementary material for: Incidence of maternal peripartum infection: A systematic review and meta-analysis
Source: PLoS Med. 2019 Dec 10;16(12):e1002984. doi: 10.1371/journal.pmed.1002984 (PMC6903710; doi:10.1371/journal.pmed.1002984)
Supplement: Attachment — Submitted filename: response to editors 311019.docx [file pmed.1002984.s014.docx]

EDITORIAL REQUESTS:

1. The Forest plots contain p values associated with heterogeneity of "p=0" and "p<0.01". We generally ask for p values to be quoted as "p<0.001" or exact values if appropriate. Please consider amending your Forest plots in this way.

We have managed to edit the forest plots to display very small p-values as p<0.001.

FIGURES and GRAPHICS:

1. Please note that your figures do not currently comply with our publication requirements. For further instructions to assist with your Figure revisions, please refer to our Figure Guidelines at: <http://journals.plos.org/plosmedicine/s/figures>.

If you need assistance, please contact us directly at [graphics@jjeditorial.com](mailto:graphics@jjeditorial.com) so we can further help with this matter. Please send any relevant source files and tell us what application(s) and what operating system you are using.

- Figure 1 looks like it was originally at a lower resolution and will appear blurry when published. This error cannot be fixed by simply increasing the resolution of your figures with image editing software. Instead, please carefully review the PLOS Figure Guidelines and provide figure files that were originally created at a minimum of 300 pixels/inch (ppi) and that are sized between 6.68-19.05 cm wide and no more than 22.225 cm tall (excluding any white space around your artwork). Additionally, please ensure your figures are in RGB colour mode.

Figure 1 was originally created in powerpoint and saved as a tif file. It has now been saved with higher resolution

- Figures 2-6 have been submitted in a layered TIF file format, which we don’t accept for figures. To ensure that your figures publish properly, please flatten the layers in each figure file (Layer→Flatten Image in Photoshop/GIMP) and re-upload. Make sure to save your TIF files with LZW Compression turned on.

Figures 2-6 and S1 Fig have had layers flattened in Photoshop and are saved with LZW compression

TABLES and BOXES:

1. Please ensure that all main tables in their entirety (including titles and footnotes), are placed after the paragraph containing their first in-text citation.

Tables 1 and 2 have been moved so they are placed after the paragraph where they are first cited.

2. Table 2 uses returns to control text wrapping. This is indicated by a [¶ or ↵] symbol when "Show Paragraph Marks" is turned on in Word. Please either split those cells into separate cells to achieve alignment or remove the returns.

The return has been removed from Table 2

SUPPORTING INFORMATION:

1. Please ensure that each supporting information file has a legend at the end of the manuscript file, after the Reference list. Currently, you have listed a legend for "S1 Fig," though this file is not uploaded to the File Inventory. Please also include a legend for your "S1 PRISMA Checklist".

The legend for S1 PRISMA Checklist has been added to the end of the manuscript and S1 Fig has been uploaded.
